# Quorum Sensing Primes the Oxidative Stress Response in the Insect Endosymbiont, *Sodalis glossinidius*

**DOI:** 10.1371/journal.pone.0003541

**Published:** 2008-10-28

**Authors:** Mauricio H. Pontes, Markus Babst, Robert Lochhead, Kelly Oakeson, Kari Smith, Colin Dale

**Affiliations:** Department of Biology, University of Utah, Salt Lake City, Utah, United States of America; Duke University Medical Center, United States of America

## Abstract

**Background:**

*Sodalis glossinidius*, a maternally transmitted bacterial endosymbiont of tsetse flies (Glossina spp.), uses an acylated homoserine lactone (AHL)-based quorum sensing system to modulate gene expression in accordance with bacterial cell density. The *S. glossinidius* quorum sensing system relies on the function of two regulatory proteins; SogI (a LuxI homolog) synthesizes a signaling molecule, characterized as N-(3-oxohexanoyl) homoserine lactone (OHHL), and SogR1 (a LuxR homolog) interacts with OHHL to modulate transcription of specific target genes.

**Methodology/Principal Findings:**

We used a tiling microarray to analyze the *S. glossinidius* transcriptome in the presence and absence of exogenous OHHL. The major finding is that OHHL increases transcription of a large number of genes that are known to be involved in the oxidative stress response. We also show that the obligate symbiont of the rice weevil, *Sitophilus oryzae* (SOPE), maintains copies of the quorum sensing regulatory genes that are found in *S. glossinidius*. Molecular evolutionary analyses indicate that these sequences are evolving under stabilizing selection, consistent with the maintenance of their functions in the SOPE symbiosis. Finally, the expression studies in *S. glossinidius* also reveal that quorum sensing regulates the expression of a cryptic, degenerate gene (*carA*) that arose from an ancient deletion in the last common ancestor of *S. glossinidius* and SOPE.

**Conclusions/Significance:**

This oxidative stress response is likely mandated under conditions of dense intracellular symbiont infection, when intense metabolic activity is expected to generate a heavy oxidative burden. Such conditions are known to arise in the bacteriocytes of grain weevils, which harbor dense intracellular infections of symbiotic bacteria that are closely related to *S. glossinidius*. The presence of a degenerate *carA* sequence in *S. glossinidius* and SOPE indicates the potential for neofunctionalization to occur during the process of genome degeneration.

## Introduction

All living organisms are dependent on their ability to sense the physical properties of their environment and respond accordingly through behavioral changes. In the context of symbiosis, the establishment and maintenance of a symbiotic relationship is dependent on the ability of both parties to perceive one another and coordinate their activities. In several recent studies it has been shown that secreted chemicals provide a means for communication between symbiotic partners. For example, chemical communication between legumes and rhizobia is known to mediate many important symbiotic interactions. These include initiation of nodulation [1], evasion of legume defenses [2]–[5], elongation of infection threads [6], [7] and development of nitrogen fixing bacteroids [4], [5], [8]. The marine symbiosis between *Vibrio fischeri* and the squid *Euprymna scolopes* is also dependent on chemical communication. In this system, chemical signaling mediates the induction of mucus secretion by the squid [9], bacterial attachment and aggregation to the host mucus [10], [11], bacterial migration to the squid light organ [12], bacterial light emission [13], and the induction of physiological [14] and developmental [15] changes in the infected squid. Models systems, such as the plant-rhizobia and squid-*Vibrio* symbioses have provided a high level of insight into the molecular processes underlying interactions between symbiotic partners. However, this knowledge is lacking for the majority of symbiotic systems, where sophisticated experimental approaches are not applicable.

Insects from many different taxonomic groups are known to maintain beneficial associations with maternally transmitted bacterial symbionts [16]. Many of these associations are ancient in origin and obligate in nature; in these cases bacterial symbionts often provide their insect hosts with essential nutrients that are lacking in the host's specialized diet [17]. More recently derived associations are typically facultative in nature; in these cases bacterial symbionts often provide ancillary benefits such as protection from natural enemies or enhanced tolerance towards conditional environmental stresses [18]. Regardless of the nature of the association, success in the symbiosis depends upon a complex interplay between bacterial symbionts and their insect hosts throughout the course of insect development and reproduction. For example, many symbiotic bacteria preferentially colonize specialized insect tissues or cells, where they reach extremely high infection densities. This feature is most pronounced in obligate symbionts, which are often found exclusively in specialized organs called bacteriomes [19]. However, facultative symbionts also tend to colonize specific host cell and tissue types at high infection densities [20], [21], [22]. In both cases, it seems likely that specific colonization patterns provide physiological benefits for both partners in the association.

In this study we describe the function of a quorum sensing system in the maternally transmitted symbiont of tsetse flies, *Sodalis glossinidius*. Quorum sensing is a chemical signaling process that allows bacteria to monitor their local population density and induce changes in behavior through the modulation of gene expression [23]. Our experiments demonstrate that *S. glossinidius* utilizes an acylated homoserine lactone (AHL)-based quorum sensing system to prime the expression of genes that ameliorate the deleterious effects of oxidative stress that are encountered at high densities of symbiont infection in host tissues.

## Results

### Characterization of the Quorum Sensing Machinery of *S. glossinidius*

**Figure 1:**
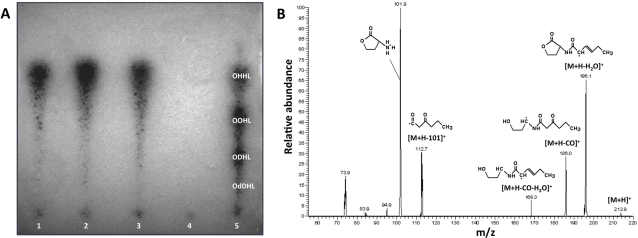
Characterization of *S. glossinidius* AHL. A. AHL samples were separated by TLC and overlaid with a live culture of an *A. tumefaciens* AHL reporter strain. Dark spots indicate the presence of AHL molecules. Lane 1: synthetic N-(3-oxohexanoyl)-homoserine lactone (OHHL); lane 2: *S. glossinidius* culture supernatant extract; lane 3: culture supernatant extract from recombinant *E. coli* expressing *S. glossinidius* SogI; lane 4: culture supernatant extract from an isogenic *E. coli* strain lacking SogI; lane 5: culture supernatant extract from *P. aeruginosa* PAO1 known to produce OHHL, N-(3-oxooctanoyl)-AHL (OOHL), N-3-(oxodecanoyl)-AHL (ODHL) and N-3-(oxododecanoyl)-AHL (OdDHL). B. Mass spectrum of the *S. glossinidius* candidate AHL ion following collision induced dissociation. The fragmentation pattern of this molecule is consistent with the structure of OHHL.

The whole genome sequence of *S. glossinidius* was found to contain a single candidate AHL synthase gene (GenBank locus tag SG0284; designated *sogI* in this report) and two candidate AHL-binding regulatory genes (SG0285 and SG1740, designated *sogR1* and *sogR2* respectively). Whereas *sogI* and *sogR1* are located adjacent to one another, *sogR2* is found at a distant location in the *S. glossinidius* chromosome. To assess the functionality of the *S. glossinidius* quorum sensing system we performed thin layer chromatography (TLC) overlay bioassays with culture supernatant extracts from *S. glossinidius* and a recombinant *E. coli* strain expressing the *S. glossinidius* SogI protein. The bioassay, performed using both *Agrobacterium tumefaciens* [24] and *Chromobacterium violaceum* (data not shown) [25] reporter strains, indicated the presence of a single AHL molecule in the supernatant extracts from both *S. glossinidius* and the recombinant *E. coli* strain (Figure 1A).

To avoid complications associated with the purification of small molecules from the complex *S. glossinidius* culture medium, we elected to perform mass spectrometric characterization of the *S. glossinidius* AHL on the culture supernatant extracted from the recombinant *E. coli* strain expressing the *S. glossinidius* SogI protein. An ethyl acetate extract of culture supernatant was fractionated on a C_18_ column by reverse-phase high performance liquid chromatography. The fractions containing the putative AHL molecule were then identified using the *A. tumefaciens* reporter strain [24] and analyzed by electrospray ionization and collision induced dissociation mass spectrometry (CID-MS). The fragmentation pattern of the candidate AHL ion obtained following CID-MS was consistent with the structure of N-(3-oxohexanoyl) homoserine lactone (OHHL; Figure 1B) [26]. This result is supported by the fact that the AHL molecules produced by *S. glossinidius* and the recombinant *E. coli* strain co-migrated with synthetic and natural forms of OHHL produced by *Pseudomonas aeruginosa* strain PAO1 in our TLC assays (Figure 1A) [27].

### Interactions Between OHHL and the SogR1 and SogR2 Transcriptional Regulators

**Figure 2:**
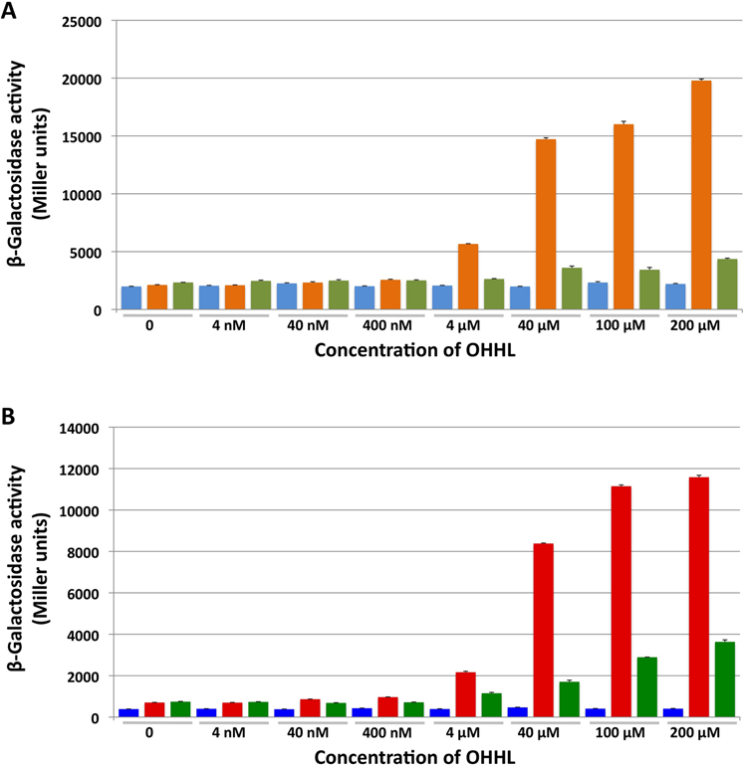
Interactions of *S. glossinidius* SogR-OHHL Complexes with *sogI* and *carA* Promoters. A. β-galactosidase activity of isogenic strains of *E. coli* harboring pMP2 (light blue), pMP3 (orange), pMP4 (light green) following exposure to different concentrations of exogenous OHHL. B. β-galactosidase activity of strains of *E. coli* harboring pMP5 (dark blue), pMP6 (red) or pMP7 (dark green) following exposure to different concentrations of OHHL. Bars indicate standard deviation.

In many bacterial species that utilize AHL quorum sensing systems, the transcription of the *luxI* homolog is autoregulated by quorum sensing [13], [28], [29], [30]. In addition, many quorum-sensing regulated genes contain an 18 to 20-bp long imperfect palindromic sequence upstream of their initiator codon. This inverted repeat sequence, known as *lux* box, serves as the binding site for LuxR transcriptional response regulators [31]. The presence of a canonical *lux* box (ACCTGAACTTTAGTACAGGT) 86 bp upstream from the *S. glossinidius sogI* start codon suggested that the transcription of this gene is autoregulated by quorum sensing. To test this hypothesis and characterize interactions between OHHL and the SogR1 and SogR2 transcriptional regulators, three recombinant plasmids were constructed (pMP2-4; Supplementary Table S1 and Figure 2A). Each of these plasmids harbors an active *sogI*-*lacZ* reporter fusion, and in addition pMP3 and pMP4 have *sogR1* and *sogR2* expressed from their native promoters. When these plasmids were transformed into isogenic strains of *E. coli*, only cells harboring pMP3 (expressing SogR1) demonstrated increased β-galactosidase activity when synthetic OHHL was added to the culture medium (Figure 2A). This shows that the interaction between OHHL and the *S. glossinidius* SogR1 protein facilitates the binding of the SogR1-OHHL complex to the *sogI* promoter, resulting in an increase in the rate of transcription of *sogI* (Figure 2A). It should also be noted that cells harboring pMP4 (expressing SogR2) demonstrated no significant increase in β-galactosidase activity when synthetic OHHL was added to the culture medium. This is likely due to a lack of interaction between SogR2 and OHHL and/or the *sogI* promoter.

### Identification of Quorum Sensing Regulated Genes in *S. glossinidius*

The *S. glossinidius* tiling microarray was used to identify genes (including putative pseudogenes) that were differentially expressed in the presence and absence of exogenous OHHL. Data was obtained from four replicate microarray experiments and analyzed using a Bayesian analysis of posterior probability. Quantitative PCR (qPCR) assays were then performed to validate the results obtained from the microarray experiments. The data obtained from the qPCR assays closely matched the data obtained from the microarray experiment. The complete dataset obtained from the microarray and qPCR experiments is presented in Supplementary Table S2.

The microarray and qPCR experiments demonstrated that expression of the *S. glossinidius sogI* gene (SG0284) was increased (4.6-fold and 7.5-fold, respectively) in response to the addition of exogenous OHHL. These results are in agreement with those results obtained from the promoter-probe experiment (Figure 2B), validating the experimental approaches used in the microarray and qPCR experiments. Only two other candidate genes (SG0585 and SG0586) displayed a >5-fold increase or decrease in expression in response to OHHL.

**Figure 3:**
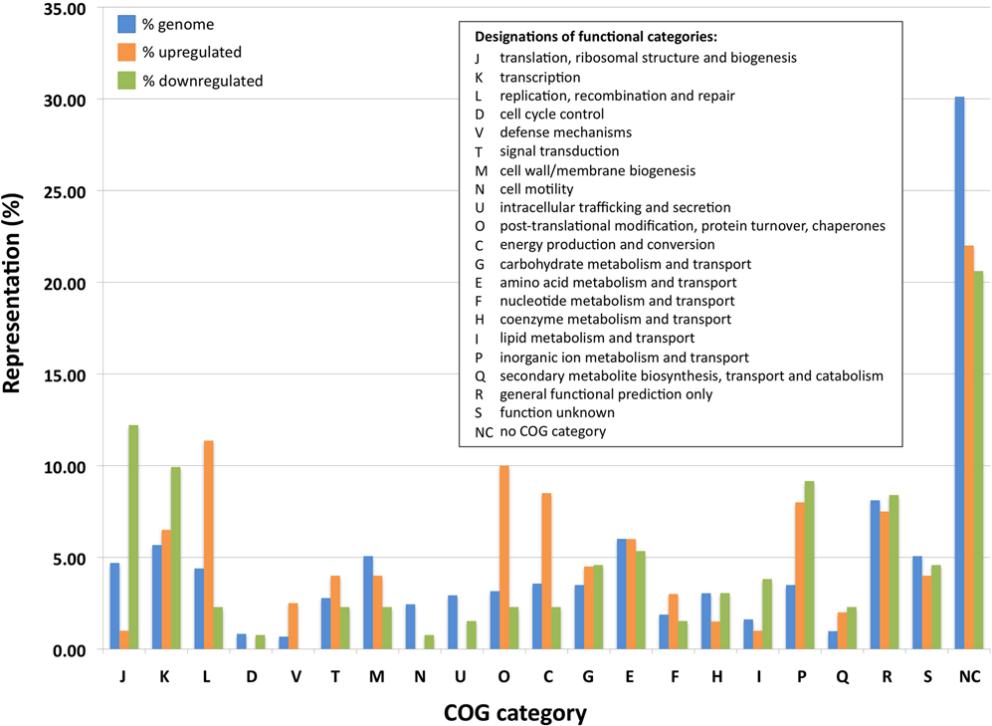
COG-Based Analysis of Microarray Expression Data. Distribution of *S. glossinidius* genes among different COG categories. Blue bars correspond to all protein coding genes in the *S. glossinidius* genome, whereas orange and green bars correspond to genes whose expression increased or decreased (respectively) by at least 1.2-fold in response to addition of OHHL. The COG classifications are described in the inset.

According to the microarray data, a substantial number of candidate genes show a statistically significant increase or decrease in expression in the range of 1.2 to 5-fold. In order to understand the biological significance of these changes in gene expression, the data was analyzed according to the clusters of orthologous groups (COG) classification (Figure 3). This revealed a bias in the representation of genes showing >1.2-fold increase in expression in response to the addition of OHHL within COG categories C (energy production and conversion), L (DNA replication, recombination and repair), O (posttranslational modification, protein turnover, chaperones) and P (inorganic ion transport and metabolism). Furthermore, a bias was also detected among those genes showing >1.2-fold decrease in expression in response to the addition of OHHL. The downregulated genes were overrepresented in COG categories J (translation), K (transcription), P and I (lipid transport and metabolism).

Many of the genes that were upregulated in response to OHHL are predicted to play a role in the oxidative stress response. These include genes predicted to encode proteins involved in the breakdown of reactive oxygen species (ROS; e.g., SG0017, SG0642, SG1609 and SG2101), repair of oxidatively damaged cellular components (SG1106 and SG1348), transport of iron and manganese (SG1516, SG1517, SG1518 and SG1519) and protein folding (e.g., SG0409, SG0584, SG0692 and SG2325; Supplementary Table S2). The addition of OHHL also increased the expression of bacterioferritin (SG2280) and genes involved in iron siderophore biosynthesis (SGP1_0041–46) [32]. Based on the fact that the siderophore biosynthetic genes of *Erwinia chrysanthemi* and *S. glossinidius* share high levels of sequence identity, it seems likely that *S. glossinidius* synthesizes an achromobactin-like, citrate-based siderophore [32]. With this in mind, it should be noted that the addition of OHHL stimulated an increase in transcription of a number of citric acid cycle enzymes, including citrate synthase (SG0871), aconitase (SG0477), isocitrate dehydrogenase (SG0700), α-ketoglutarate dehydrogenase (SG0876, SG0877), succinyl-CoA synthase (SG0878, SG0879) and succinate dehydrogenase (SG0872–75; Supplementary Table S2). It is possible that the resulting increase in TCA cycle activity might be required to provide sufficient citric acid for siderophore biosynthesis at high cell density.

Among those genes showing decreased expression in the presence of OHHL, the largest representational bias was found in COG category J (Figure 3). This includes several genes encoding subunits of the 30S (SG0380, SG0412 and SG2269) and 50S ribosomal proteins (SG0133, SG1420, SG1421, SG1572, SG2207, SG2252, SG2270, SG2271 and SG2273). In addition, genes encoding a 16S rRNA pseudouridylate synthase A (SG1570), a tRNA/rRNA methyltransferase (SG1908) and a putative ribosome modulation factor (SG1025) also displayed reduced expression in the presence of OHHL. This leads to an interesting hypothesis – perhaps quorum sensing restricts the growth rate of *S. glossinidius* at high infection density by reducing the activities of enzymes involved in translation. However, we were unable to detect any difference in the growth rate of laboratory cultures of *S. glossinidius* in the presence and absence of exogenous OHHL (data not shown).

The *S. glossinidius* gene showing the largest reduction in expression in response to OHHL is annotated as a dethiobiotin synthase (*bioD*; SG1466). *Sodalis glossinidius* is unusual because it maintains two phylogenetically distinct ORFs (SG0906 and SG1466) encoding putative *bioD* homologs. SG0906, whose expression is not affected by OHHL, is located within an operon alongside other genes known to be involved in biotin biosynthesis (*bioABFC*; SG0902–SG0905). Thus, SG0906 appears to be a component of the canonical biotin biosynthesis gene cluster found in a wide range of bacteria [33]. SG1466 is also unusual because the C-terminal end of the predicted translation product maintains a sugar transporter domain that is not found in any of the other dethiobiotin synthetases in the GenBank database. Furthermore, SG1466 is not located within a cluster of genes involved in biotin biosynthesis. Thus, SG1466 may have evolved to provide a novel function in *S. glossinidius*.

### Biochemical Detection of Siderophores

**Figure 4:**
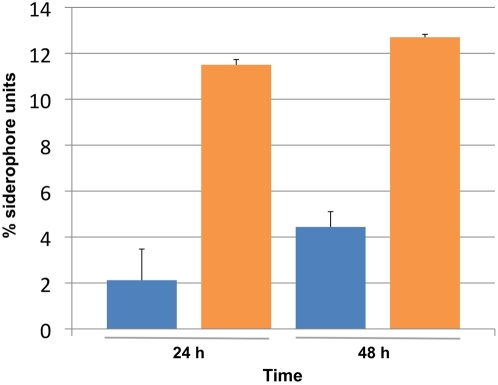
Influence of OHHL on Iron Siderophore Production in *S. glossinidius*. Siderophore units were estimated from samples of *S. glossinidius* culture supernatant in the presence (orange) and absence (blue) of exogenous OHHL.

Iron siderophore assays were performed to quantitate siderophore production in *S. glossinidius* cultures maintained at low cell density in the presence and absence of exogenous OHHL. The addition of OHHL provides an “artificial” quorum that stimulates a 5-fold increase in siderophore production in culture media over the course of 24 h (Figure 4). After 48 h, this difference is reduced to 3-fold as the uninduced cultures reach a cell density sufficient to provide a “natural” quorum. These results demonstrate that *S. glossinidius* increases production of iron siderophores in a cell density-dependent manner in response to the quorum sensing signaling molecule, OHHL. This is in agreement with the microarray and qPCR data showing that *S. glossinidius* increases transcription of genes involved in siderophore biosynthesis in response to the addition of OHHL.

### Degeneration of Carbapenem Biosynthesis Genes

**Figure 5:**
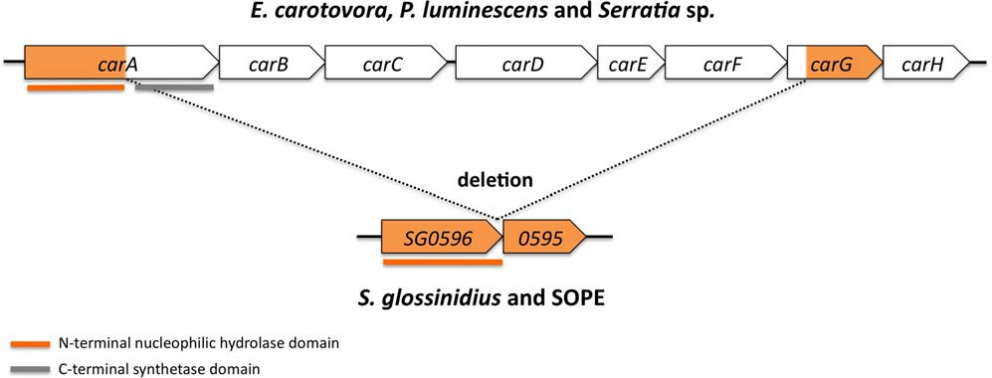
Degeneration of a Carbapenem Biosynthesis Gene Cluster in *S. glossinidius* and SOPE. Carbapenem production (*carABCDE*) and resistance (*carFG*) genes are clustered in the genomes of *E. carotovora*, *P. luminescens* and *Serratia* sp. *Sodalis glossinidius* and SOPE maintain truncated copies of *carA* and *carG* that likely arose through the deletion of intervening DNA.

According to the microarray data, two candidate genes (SG585 and SG586) demonstrated a >5-fold increase in transcription in response to the addition of OHHL. The GenBank annotation of the *S. glossinidius* genome sequence indicates that SG585 and SG586 are genic components of a biosynthetic pathway that leads to the production of a β-lactam antibiotic called carbapenem. Carbapenem is produced by a number of Gram-negative bacteria including *Erwinia* spp., which is one of the closest free-living relatives of *S. glossinidius* [34]–[37]. Furthermore, carbapenem production is often controlled by quorum sensing, and in *Erwinia carotovora* the signaling molecule is known to be OHHL [38]. The genes involved in the biosynthesis of carbepenem (*carABCDE*) are normally localized in a cluster alongside genes that are known to confer a carbapenem resistance phenotype (*carFG*; Figure 5) [36]. In *S. glossinidius*, most of the genes required for carbapenem biosynthesis and resistance have been lost as a result of a deletion between the C-terminal domain of CarA (SG0586) and the N-terminal domain of CarG (SG0585). Therefore, only the N-terminal domain of CarA and the C-terminal domain of CarG remain. However, several lines of evidence indicate that the genic remnant of *carA* retains functionality. First, it should be noted that the weevil symbiont, SOPE, also maintains truncated copies of *carA* and *carG* that share an almost identical intergenic deletion (Figure 5). Although it is possible that such deletions occurred independently in the lineages leading to *S. glossinidius* and SOPE, it seems more likely (based on parsimony) that a single deletion took place in the common ancestor of these symbionts. Furthermore, if the *carA* genes of *S. glossinidius* and SOPE were inactive, we would anticipate a high ratio of nonsynonymous (nonsilent) to synonymous (silent) substitutions (*d*N/*d*S). However, our molecular evolutionary analysis (detailed below) shows that *carA* has a low *d*N/*d*S ratio, compatible with the preservation of gene function under stabilizing selection. Second, the truncation in the *S. glossinidius* and SOPE *carA* sequences occurs at a point in the conceptual CarA protein that links an N-terminal nucleophilic hydrolase domain and a C-terminal synthase domain (Figure 5). Thus, the *carA* sequences in *S. glossinidius* and SOPE retain only the nucleophilic hydrolase domain of the ancestral *carA*. Third, the conceptual nucleophilic hydrolase domain in the *S. glossinidius* and SOPE *carA* sequences retain many of the key residues known to be important for amino acid amidohydrolysis (data not shown). Thus, we postulate that the truncated *carA* sequences in *S. glossinidius* and SOPE have acquired a novel functionality, unrelated to antibiotic biosynthesis, as a result of deletion.

Interestingly, *E. carotovora* is known to maintain at least two homologs of LuxR, one of which (CarR) is dedicated to the regulation of the carbapenem gene cluster [39], [40]. Since *S. glossinidius* is also known to maintain two LuxR homologs, we decided to investigate interactions between SogR1, SogR2 and the *carA* promoter. Plasmids pMP5–7 were constructed by replacing the *sogI*-*lacZ* fusions from pMP2–4, respectively, with a *carA*-*lacZ* fusion. *Escherichia coli* strains harboring these plasmids were then tested for β-galactosidase activity in the presence of increasing concentrations of OHHL (Figure 2B). Interestingly, only cells harboring pMP6 (expressing SogR1) demonstrated increased β-galactosidase activity in response to OHHL. This shows that the canonical SogR1 protein is involved in the cell density dependent regulation of *carA*, and that SogR2 is therefore not a functional homolog of CarR.

### Molecular Evolutionary Analyses

In order to understand the molecular evolutionary genetics of the quorum sensing system in *S. glossinidius*, we obtained homologs of *sogI*, *sogR1*, *sogR2* and the truncated *carA* ORF from an unfinished (6–8× coverage) genome sequence of the *Sitophilus oryzae* primary endosymbiont (SOPE), which is known to be closely related to *S. glossinidius* [41], [42]. The putative coding sequences of *sogI*, *sogR1* and *sogR2* were found to be intact in SOPE, indicating their potential to encode proteins that serve as regulators of quorum sensing in the weevil symbiosis.

**Figure 6:**
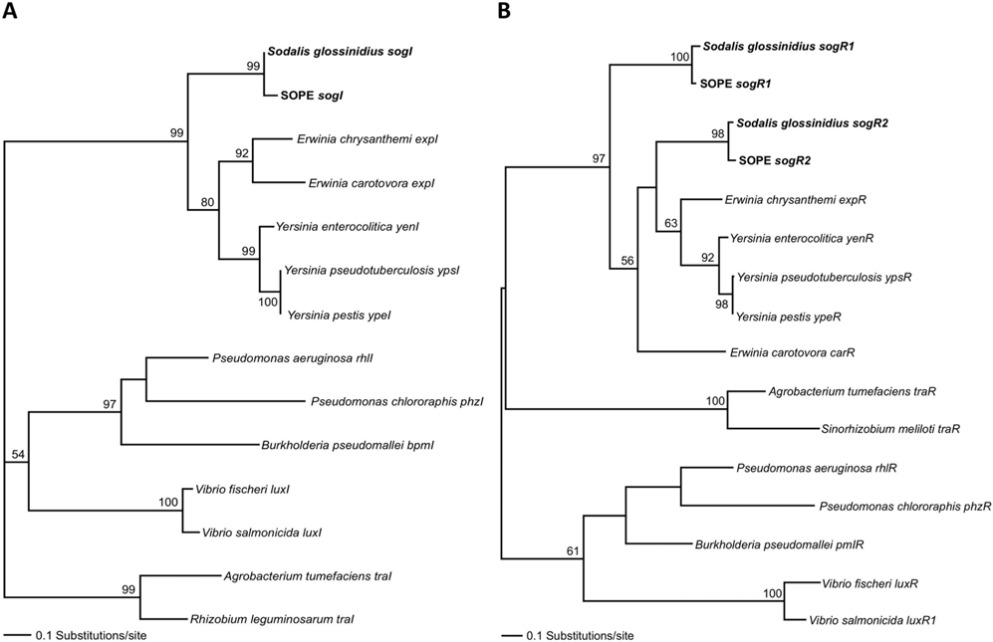
Common Ancestry of *S. glossinidius* and SOPE Quorum Sensing Regulatory Genes. Maximum likelihood phylogenetic analyses provide support for the common ancestry of the *sogI* (A), *sogR1* and *sogR2* (B) sequences in *S. glossinidius* and SOPE. Bootstrap values >50% are shown adjacent to each node. GenBank accession numbers for the sequences used in these analyses are provided in Supplementary Information S1.

The *luxI* and *luxR* homologs from *S. glossinidius*, SOPE and other Gram negative bacteria were used to construct maximum likelihood (ML) phylogenetic trees. Both the *luxI* and *luxR* trees were supported by more than 50% of ML bootstrap resamples at every node. Four major clades were resolved in the *luxI* tree (Figure 6A), each with >95% bootstrap support. The *luxI* homologs from *S. glossinidius* and SOPE were placed in a clade with 99% bootstrap support alongside *luxI* homologs from *Erwinia* spp. and *Yersinia* spp., which are the closest free-living relatives of *S. glossinidius* [43]. Furthermore, the *S. glossinidius* and SOPE sequences were placed together in a sub-clade with 100% bootstrap support, indicating their close ancestry. The same pattern of relationships was resolved in the *luxR* tree (Figure 6B). Both the *luxR* homologs from *S. glossinidius* and SOPE were placed in a clade along with *luxR* homologs from *Erwinia* spp. and *Yersinia* spp. with 97% bootstrap support. Interestingly, the *S. glossindius* and SOPE *sogR1* and *sogR2* sequences were placed in separate sub-clades with 100% and 98% bootstrap support, respectively. Thus, it seems likely that these two genes in *S. glossinidius* and SOPE have independent phylogenetic origins and did not arise from a recent gene duplication event.

Estimating *d*N/*d*S (the number of nonsynonymous base substitutions per nonsynonymous site versus the number of synonymous base substitutions per synonymous site) provides a means to assess the strength and direction of natural selection acting upon genic (coding) sequences. In bacteria, pairwise estimates of *d*N/*d*S typically fall within the range of 0.04–0.2 for functional genes evolving under stabilizing selection [44]. Conversely, genic sequences that have been rendered inactive (pseudogenes) are expected to have *d*N/*d*S ratios that approach parity. Since we made no direct assessment of the functionality of the truncated *carA* homologs in *S. glossinidius* and SOPE, or the *lux* gene homologs in SOPE, we reasoned that it would be useful to perform tests of selection on these putative coding sequences. Thus, we obtained pairwise estimates of *d*N/*d*S for the *luxI*, *luxR* and *carA* sequences from *S. glossinidius*, SOPE and close free-living relatives (Table 1). Notably, the estimates of *d*N/*d*S in pairwise sequence comparisons between *S. glossinidius* and SOPE for *sogI* (0.071), *sogR1* (0.061), *sogR2* (0.116) and the truncated *carA* homolog (0.095) are all within the range expected for genes evolving under stabilizing selection. This suggests that *sogI*, *sogR1*, *sogR2* and the truncated *carA* all remain functional in *S. glossinidius* and SOPE.

**Table 1:** *d*N∶*d*S ratios computed from pairwise comparisons of genes involved in quorum sensing in *S. glossinidius*, SOPE and related free-living bacteria.

Sequence Pairs	*d*N	*d*S	*d*N/*d*S Ratio
*S. glossinidius carA*- SOPE *carA*	0.058	0.610	0.095
*S. glossinidius carA- E. carotovora carA*	0.698	1.504	0.464
SOPE *carA*- *E. carotovora carA*	0.694	1.766	0.393
*S. glossinidius sogI*- SOPE *sogI*	0.036	0.506	0.071
*S. glossinidius sogI*- *Y. pestis ypeI*	0.466	1.800	0.259
SOPE *sogI*- *Y. pestis ypeI*	n/d*	n/d*	n/d*
*S. glossinidius sogR1*- SOPE *sogR1*	0.039	0.635	0.061
*S. glossinidius sogR1*- *Y. pestis ypeR*	0.533	1.484	0.359
SOPE *sogR1*- *Y. pestis ypeR*	n/d*	n/d*	n/d*
*S. glossinidius sogR2*- SOPE *sogR2*	0.045	0.388	0.116
*S. glossinidius sogR2*- *E. carotovora expR2*	n/d*	n/d*	n/d*
*S. glossinidius sogR2*- *E. carotovora carR*	n/d*	n/d*	n/d*
SOPE *sogR2*- *E. carotovora expR2*	0.497	1.970	0.252
SOPE *sogR2*- *E. carotovora carR*	n/d*	n/d*	n/d*
*E. carotovora expR2- E. carotovora carR*	n/d*	n/d*	n/d*

* n/d – not determined due to saturation (*d*S>2) at synonymous sites. GenBank accession numbers for the sequences used in these analyses are provided in Supplementary Information S1.

## Discussion

*Sodalis glossinidius*, an endosymbiotic bacterium found in tsetse flies (*Glossina* spp.), uses an AHL-based quorum sensing system to modulate gene expression according to cell density. Since *S. glossinidius* has no known lifestyle component outside of the insect host, the quorum sensing system must play an important role in the regulation of bacterial gene expression during symbiosis. The *S. glossinidius* quorum sensing system utilizes at least two distinct regulatory proteins, designated SogI and SogR1. SogI is an AHL synthase that is responsible for the synthesis of a signaling molecule, OHHL. SogR1 is a DNA-binding transcriptional regulator that interacts with OHHL and modulates the expression of target genes in accordance with bacterial population density. *Sodalis glossinidius* also maintains a second LuxR homolog (SogR2) but the function of this protein was not elucidated in this study.

In the current study, we used a tiling microarray to identify genes in the *S. glossinidius* genome whose expression is modulated in the presence of the quorum sensing signaling molecule, OHHL. The most striking finding was that OHHL increased the expression of a large number of genes that are known to be involved in the cellular response to oxidative stress. These includes *oxyR*, the positive regulator of hydrogen peroxide inducible genes [45], and numerous genes whose protein products are known to be involved in the direct detoxification of ROS [46], [47], including catalase (*katA*), manganese superoxide dismutase (*sodA*), peroxidase (*ahpC*) and glutathione S-transferase (*yfcG*). We also detected increased expression of genes encoding proteins known to be involved in the repair of cellular components following ROS-mediated damage [48]–[54]. These include thioredoxin reductase (*trxB*), methionine sulfoxide reductase B (*msrB*) and several chaperones known to be involved in protein refolding and maturation under oxidative stress (e.g., *clpB*, *dnaK*, *grpE*, *htpG*, *ibpA*, *nfuA*).

The microarray data also shows that OHHL modulated the expression of genes involved in metal ion transport and storage. In many bacteria, the expression of genes involved in iron and manganese transport and metabolism is coordinately regulated with genes involved in the oxidative stress response [55], [56]. Both manganese and iron are known to serve as essential co-factors in a number of enzymes that detoxify ROS (e.g., catalase and superoxide dismutase). Also, soluble iron is known to contribute directly to oxidative stress by catalyzing the formation of free radicals through the Fenton reaction [46]. OHHL was also found to increase the expression of genes involved in the production of iron siderophores and bacterioferritin, which have been shown to play a role in ameliorating oxidative stress by removing toxic iron from solution and (in the case of bacterioferritin) serving as iron storage complexes for heme enzymes [57]–[60]. In *S. glossinidius*, OHHL also increased expression of genes encoding an iron/manganese transport system (*sitABCD*) that is known to be associated with resistance to oxidative stress in other bacteria [61], [62], [63]. Furthermore, biochemical assays showed that *S. glossinidius* produced and secreted increased amounts of iron siderophores into culture media in the presence of exogenous OHHL. Finally, it should be noted that OHHL increased transcription of a cluster of genes encoding a polyol metabolism and transport system in *S. glossinidius* (SG0608–SG0614; 1.4–3 fold upregulation). This is conspicuous because polyols are known to be abundant in animal cells under conditions of hyperglycemia [64].

Many insect endosymbionts, including *S. glossinidius*, are known to maintain extremely high infection densities within host tissues and cells over prolonged periods of time [65], [66], [67]. In a recent study, Heddi et al. [68] compared the metabolic and transcriptional profiles of maize weevil bacteriocytes in the presence and absence of a symbiotic bacterium (SZPE) that is another close relative of *S. glossinidius*. Notably, symbiont-infected cells were found to display an unusual profile of carbohydrate transport and metabolism characterized by the induction of the polyol pathway through increased expression of aldose reductase enzymes. This adaptation is predicted to occur in response to the high carbohydrate uptake and intense metabolic activity in densely infected bacteriocytes, which generates conditions analogous to those found in mammalian diabetic cells. One critical consequence of this intense metabolic activity is the generation of increased concentrations of ROS [64]. Under these conditions, the weevil bacteriocytes were found to display increased expression of genes encoding proteins that are expected to ameliorate the deleterious effects of ROS.

Many bacteria are known to undergo an “adaptive response” upon challenge with a sublethal dose of ROS [69]. The sublethal challenge serves to prime the expression of a large number of genes involved in the oxidative stress response, rendering the cells more resistant to a subsequent lethal dose. Our findings suggest that quorum sensing serves to prime the cellular response to oxidative stress in *S. glossinidius* in a similar way. The use of a quorum sensing system for this purpose makes sense because the concentrations of ROS are expected to increase in accordance with the density of bacterial infection in host cells and tissues. Thus it seems that symbionts and host cells work together to modulate their gene expression profiles and metabolic activities to minimize the deleterious effects of oxidative stress encountered during their symbiotic interactions. This likely represents a key adaptation in the symbiotic relationship because it allows host cells to sustain dense intracellular symbiont infections.

The discovery of complementary responses towards oxidative stress in the weevil bacteriocytes and in *S. glossinidius* prompted us to search for the presence of quorum sensing regulatory genes in the grain weevil symbiont, SOPE. The SOPE genome sequence was indeed found to contain intact homologs of the *S. glossinidius sogI*, *sogR1* and *sogR2* genes. Phylogenetic analyses indicate that the *lux* genes were present in the last common ancestor of *S. glossinidius* and SOPE, so their acquisition likely predates the origin of the symbiotic associations involving these bacteria. Although the functions of the SOPE *lux* genes have not been defined in this study, molecular evolutionary analyses indicate that *sogI*, *sogR1* and *sogR2* are evolving under stabilizing selection, consistent with the maintenance of gene function in the weevil symbiosis. Given that many bacterial symbionts, including *S. glossinidius* and SOPE, are known to attain extremely high infection densities in host tissues in spite of the burden of oxidative stress, it seems likely that such high infection densities confer important advantages within the symbiosis. For this reason, we expect other insect-bacterial associations to have similar adaptations to deal the challenge of oxidative stress. Furthermore, it is noteworthy that insect symbionts such as *S. glossinidius* and members of the candidate genus *Arsenophonus* have proved difficult to isolate and manipulate in pure culture because they demonstrate a high level of sensitivity towards oxidative stress when cultures are maintained on agar plates at low cell densities [70]. Thus, increasing our understanding of the oxidative stress response in symbiosis may also contribute to the development of improved techniques for the culture and manipulation of symbionts.

Following the establishment of symbiosis, the switch to a strictly vertical mode of transmission and the adoption of a static lifestyle leads to a process of genome streamlining in symbiotic bacteria that is characterized by the progressive inactivation and deletion of genes evolving under relaxed selection [18]. This evolutionary trajectory is largely degenerative and irreversible because symbionts have little or no opportunity to replenish their genetic inventory through parasexual recombination. For example, in *S. glossinidius*, only 50% of the whole genome sequence is anticipated to encode functional proteins [43]. The remaining DNA is composed largely of pseudogenes – genes with reading frames truncated by more than 50% as a result of frameshifts and/or premature stop codons. In the current study we found that one such “pseudogene,” *carA* (formally a component of an antibiotic biosynthesis gene cluster), unexpectedly displayed a 5-fold increase in transcription in response to OHHL. Pairwise analyses of *d*N/*d*S ratios show that *carA* is evolving under strong stabilizing selection in *S. glossinidius* and SOPE. Taken together, these observations suggest that *carA* is maintained as a functional protein coding gene, albeit in a truncated form. This illustrates the potential for adaptive neofunctionalization to occur as a component of the process of genome degeneration and streamlining.

## Methods

### Bacterial Strains and Culture Conditions

A complete list of plasmids and strains used in this study is provided in Supplementary Table S1. *Sodalis glossinidius* was maintained at 25°C in the semi-defined liquid Mitsuhashi-Maramorosch (MM) medium as described previously [65] or in a defined liquid medium containing 0.15 g/L CaCl_2_, 0.046 g/L MgCl_2_, 0.2 g/L KCl, 7.0 g/L NaCl, 4.0 g/L glucose and 6 g/L deferrated casamino acids. *Escherichia coli* strains were maintained at 37°C in either Luria-Bertani (LB) medium or in M9 liquid minimal medium salts supplemented with 0.24 mg/L MgSO_4_, 0.01 mg/L CaCl_2_, 4 g/L lactose and 2 g/L casamino acids. *Agrobacterium tumefaciens* KYC55 (pJZ372) (pJZ384) (pJZ410) was maintained at 28°C in AT minimal medium [24]. *Chromobacterium violaceum* CV026 and *P. aeruginosa* PAO1 were maintained at 30°C and 37°C, respectively, in LB medium [25]. Where appropriate, antibiotics were added to the media at the following concentrations: 100 µg/ml of ampicillin, 100 µg/ml of gentamycin, 30 µg/ml (chromosomal integrations) or 50 µg/ml (high copy number plasmids) of kanamycin, 100 µg/ml of spectinomycin and 1.5 µg/ml of tetracycline.

### Extraction of Culture Supernatants

Acylated homoserine lactones were extracted from culture supernatants as described by Shaw et al. [71]. Briefly, 500 ml of a stationary phase culture of *S. glossinidius* (grown in liquid MM medium), *E. coli* [TOP10 and TOP10 (pSGI) grown in defined liquid medium] and *P. aeruginosa* were pelleted by centrifugation (8,000×g, 20 min., 4°C). The resulting culture supernatants were filtered through 0.2 µm pore-size membrane filters (Nalgene Labware, Cat. No. 154-0020) and extracted twice with an equal volume of ethyl acetate. The extracts were combined, dried with anhydrous magnesium sulfate, filtered, evaporated using a vacuum centrifuge and resuspended in 500 µL of acetonitrile.

### Thin Layer Chromatography Overlay Assay

Samples were chromatographed on C_18_ reverse-phase TLC plates (Whatman, Cat. No. 4801-800) using methanol∶water (60∶40). Following development, plates were dried and overlaid with live cultures of *A. tumefaciens* KYC55 (pJZ372) (pJZ384) (pJZ410) or *C. violaceum* CV026 indicator strains as described previously [24], [25].

### High Performance Liquid Chromatography and Mass Spectrometry Analysis

*S. glossinidius* and *E. coli* TOP10 (pSGI) culture supernatant extractions were fractionated using reverse-phase high performance liquid chromatography. The samples were chromatographed on a C_18_ column with a linear water-acetonitrile gradient (0–40%) containing 0.1% trifluoroacetic acid (v/v). The eluted fractions were screened for the presence of AHL with the *A. tumefaciens* biosensor strain [24]. Bioactive fractions from the *E. coli* TOP10 (pSGI) sample were evaporated using a vacuum centrifuge and resuspended in methanol. The samples were analyzed by electrospray ionization and CID-MS using a Micromass Quattro II - Triple Quadrupole Mass Spectrometer under positive-ion conditions. Fractions were injected into the mass spectrometer in a solvent containing 50% methanol, 49.9% water and 0.1% formic acid at a flow rate of 5 µL/min.

### β-Galactosidase Assay

*E. coli* strain BW25113, harboring specific reporter plasmids, were grown overnight in LB medium. Overnight cultures were diluted 1∶500 in fresh medium containing 0, 4 nM, 40 nM, 0.4 µM, 4 µM, 40 µM, 100 µM or 200 µM of N-(3-oxohexanoyl)-homoserine lactone (Sigma Aldrich, Cat. No. K3255). The cultures were maintained for 3 hours at 37°C and β-galactosidase activity was measured as described by Miller [72]. All assays were conducted in triplicates.

### Artificial Induction of Quorum and RNA Isolation

A culture of *S. glossinidius* was grown to early log phase (OD_600_≈0.04, approximately 1.5×10^7^ CFU/ml) in MM liquid medium. This culture was separated to yield eight cultures of equal volume and 100 µM of N-(3-oxohexanoyl)-homoserine lactone (Sigma Aldrich) was added to four of the replicates. After 12 hours of incubation at 25°C, the cells were harvested by centrifugation (5,000×g, 10 min., 4°C). RNA was prepared using the SV Total RNA Isolation System (Promega, Cat. No. Z3100), according to the kit instructions. Aliquots of the RNA samples were treated with DNase I (Ambion, Cat. No. AM1907) to remove contaminating DNA. RNA samples were then reverse transcribed using the TaqMan Reverse Transcription Reagents (Applied Biosystems, Part No. N808-0234), according to the manufacturers instructions. Artificial induction of quorum was verified by measuring relative transcript levels of *sogI* in induced versus uninduced samples using qPCR.

### Microarray Expression Analyses and Quantitative PCR

A custom tiling microarray was designed using the eArray Design Creation online application (Agilent Technologies). The array consisted of approximately 40,000 sixty-mer oligonucleotide probes, one probe per 200 bp, covering the entire *Sodalis glossinidius* genome (GenBank accession number AP008232.1). Standard positive and negative control features were also included on the array. Microarrays were printed using Agilent SurePrint technology in the 4×44 k slide format.

Microarray hybridization was performed according to the Agilent Technologies protocol, with the following modifications. Briefly, poly(A) tails were added to the 3′ end of the RNA molecules using the Ambion Poly(A) tailing kit (Cat. No. 1350). The polyadenylated RNA was then used as a template to generate fluorescently labeled cRNA using the Agilent Two-Color Low RNA Input Linear Amplification Kit, labeling OHHL+ RNA with cyanine 3-cytosine triphosphate (CTP) and OHHL- RNA with cyanine 5-CTP, and *vice versa*. Fluorescently labeled cRNA samples (825 ng each), in addition to Agilent RNA spike-in controls, were then fragmented and hybridized to the tiling microarray using the Agilent 2-color GE Hybridization/Wash protocol. Hybridized slides were then scanned in an Agilent Technologies G2505B Microarray Scanner at 5 µm resolution, performing a simultaneous detection of Cyanine-3 and Cyanine-5 signal on the hybridized slide. An extended dynamic range scan was then accomplished by performing a primary scan at 100% laser power and a secondary scan at 10% power; the former used to calculate intensities for non-saturating features, and the latter used to calculate intensities for saturating features. The scanned microarray image files were then loaded into Agilent Feature Extraction Software (v. 9.5.1), which was used to perform calculations that included feature intensities, background measurements, and statistical analyses. To control for variation in individual probe hybridization efficiency and fluorescence intensity, mechanically sheared whole genomic DNA was used as a template for cRNA hybridization on a separate array. Differences in individual probe intensities were used to normalize the raw experimental data. Hierarchical clustering analyses correctly broke down normalized data by sample treatment (+/− OHHL, dye type; r^2^ values >0.95).

Statistical analysis of the results was performed using Tiling Microarray Analysis Tools 2 (http://sourceforge.net/projects/timat2). CyberT was used to estimate a confidence in the differential expression by calculating the posterior probability of differential expression [73]. Affymetrix's Integrated Genome Browser was used to visualize the microarray analysis data (http://www.affymetrix.com/support/developer/tools/download_igb.affx).

Microarray results were verified by qPCR. Briefly, RNA samples from the four biological replicates were pooled together, and subjected to DNase I treatment (Ambion, Cat. No. AM1907) until no DNA could be detected by qPCR. RNA samples were then reverse transcribed using the TaqMan Reverse Transcription Reagents (Applied Biosystems, Part No. N808-0234), according to the manufacturers instructions. Reactions were performed in triplicates using iQ Supermix (Bio-Rad, Cat. No. 170-8862), and the samples were quantitated using an iCycler iQ Multicolor Real-Time PCR Detection System (Bio-Rad). Relative transcript levels were estimated using the standard curve method described previously by Dale et al. [74]. In order to validate the microarray results, 10 different loci that were either upregulated, downregulated or displayed no changed in expression profile in the microarray experiment were selected for the qPCR verification.

### Siderophore Quantitative Assay

A 150 ml culture of *S. glossinidius* was grown to early log phase (OD_600_≈0.04, approximately 1.5×10^7^ CFU/ml) in MM liquid medium. The cells were pelleted by centrifugation (4,500×g, 10 min., 15°C) and washed twice in an equal volume of 0.85% NaCl solution. After a third centrifugation, the cells were resuspended in 15 ml of 0.85% NaCl solution and 1 ml of this cell suspension was used to inoculate six replicates of 30 ml of defined medium. 100 µM N-(3-oxohexanoyl)-homoserine lactone (Sigma Aldrich, Cat. No. K3255) was added to three of the replicates. After 24 and 48 hours of incubation at 25°C, culture aliquots were pelleted by centrifugation (8,000×g, 20 min., 4°C), supernatants were filtered through 0.2 µm pore-size membrane filters (Millipore, Cat. No. SLGP033RB) and siderophore units were estimated using the chrome azurol S liquid assay [75]. To control for the effects of N-(3-oxohexanoyl)-homoserine lactone as a potential iron chelating agent [76], 100 µM of N-(3-oxohexanoyl)-homoserine lactone was added to all supernatants and reference readings prior to quantitation.

### Phylogenetic and Molecular Evolutionary Analyses

Phylogenetic analyses were performed on homologs of *luxI* and *luxR* from *S. glossinidius*, a closely related grain weevil endosymbiont (SOPE) [67], and other closely related Gram negative bacteria known to maintain AHL-based quorum sensing systems. The nucleotide sequences of *luxI* and *luxR* were aligned according to the corresponding protein sequence alignments generated in CLUSTAL, to provide in-frame nucleotide sequence alignments. All third codon position characters were then excluded from subsequent analyses to improve the signal to noise ratio (third position characters were deemed uninformative due to high levels of substitution at synonymous sites). Maximum likelihood (ML) analyses were performed in PAUP 4.0 [77], using the heuristic tree-bisection-reconnection algorithm. ML parameters were estimated from an initial neighbor-joining tree and optimized in the construction of ML trees using variable base frequencies, a symmetrical substitution matrix and gamma distributed rate variation among sites. Bootstrap values were obtained from analysis of 100 replicates.

Pairwise molecular evolutionary sequence analyses were performed in MEGA 4.0 [78]. Nucleotide sequence alignments were generated in frame, as described above. The frequencies of synonymous and nonsynonymous substitutions (*d*S and *d*N, respectively) were estimated using the Kumar method. Z-tests of selection were then used to estimate the probability of rejecting a null hypothesis of strict neutrality in favor of an alternative hypothesis of stabilizing (purifying) selection.

## Supporting Information

Table S1 Plasmids and Strains Used in this Study
(0.06 MB DOC)

Table S2 Results from Microarray Expression Analyses
(2.68 MB DOC)

Supplementary Information S1 (0.03 MB DOC)
