# Supplementary material for: Quorum Sensing Primes the Oxidative Stress Response in the Insect Endosymbiont, Sodalis glossinidius
Source: PLoS One. 2008 Oct 28;3(10):e3541. doi: 10.1371/journal.pone.0003541 (PMC2568817; doi:10.1371/journal.pone.0003541)
Supplement: Table S1 — Plasmids and Strains Used in this Study (0.06 MB DOC) [file pone.0003541.s001.doc]

**Supplementary Table S1. Plasmids and Strains Used in this Study.**

| **Plasmid or strain** | **Relevant characteristic** | **Source** |
| --- | --- | --- |
| **Plasmids** | | |
| pCR Blunt II TOPO | Kan^R^, Neo^R^, general cloning vector | Invitrogen |
| pMP1 | Amp^R^, pRS415 derivative containing pUC19 multiple cloning site in its SmaI site | This study |
| pMP2 | Amp^R^, pMP1 containing *S. glossinidius* *sogI* (GenBank locus tag SG0284) promoter transcriptionally fused to *lacZ* | This study |
| pMP3 | Amp^R^, pMP2 containing *S. glossinidius* *sogR1* gene (SG0285) expressed from its native promoter | This study |
| pMP4 | Amp^R^, pMP2 containing *S. glossinidius* *sogR2* gene (SG1740) expressed from its native promoter | This study |
| pMP5 | Amp^R^, pMP1 containing *S. glossinidius* *carA* homologue (SG0586) promoter transcriptionally fused to *lacZ* | This study |
| pMP6 | Amp^R^, pMP5 containing *S. glossinidius* *sogR1* gene (SG0285) expressed from its native promoter | This study |
| pMP7 | Amp^R^, pMP5 containing *S. glossinidius* *sogR2* gene (SG1740) expressed from its native promoter | This study |
| pRS415 | Amp^R^, promoterless *lacZ* vector | [79] |
| pSGI | Kan^R^, Neo^R^, pCR Blunt II TOPO carrying *S. glossinidius* *sogI* gene (SG0284) expressed from the Plac promoter | This study |
| pUC19 | Amp^R^, general cloning vector | Invitrogen |
| **Strains** | | |
| *Agrobacterium tumefaciens* KYC55 (pJZ372) (pJZ384) (pJZ410) | Gen^R^, Spc^R^, Tet^R^, acylated homoserine lactone (AHL) reporter strain | [24] |
| *Chromobacterium violaceum* CV026 | Kan^R^, AHL reporter strain | [25] |
| BW25113 | *E. coli* K-12 derivative | [80] |
| BW25113 (pMP2) | Amp^R^, BW25113 harboring pMP2 | This study |
| BW25113 (pMP3) | Amp^R^, BW25113 harboring pMP3 | This study |
| BW25113 (pMP4) | Amp^R^, BW25113 harboring pMP4 | This study |
| BW25113 (pMP5) | Amp^R^, BW25113 harboring pMP5 | This study |
| BW25113 (pMP6) | Amp^R^, BW25113 harboring pMP6 | This study |
| BW25113 (pMP7) | Amp^R^, BW25113 harboring pMP7 | This study |
| *Pseudomonas aeruginosa* PAO1 | AHL producing, reference strain | Sang-Jin Suh, Auburn University |
| *Sodalis glossinidius* str. 'morsitans' | Organism of study | Serap Aksoy, Yale University |
| TOP10 | Str^R^, *E. coli* MC1061 derivative | Invitrogen |
| TOP10 (pSGI) | Kan^R^, Neo^R^, Str^R^, TOP10 harboring pSGI | This study |

Abreviations: Amp^R^- ampicillin resistance; Gen^R^- gentamycin resistance; Kan^R^- kanamycin resistance; Neo^R^- neomycin resistance; Spc^R^- spectinomycin resistance; Str^R^- streptomycin resistance; Tet^R^- tetracyclin resistance.
