# Supplementary material for: Quorum Sensing Primes the Oxidative Stress Response in the Insect Endosymbiont, Sodalis glossinidius
Source: PLoS One. 2008 Oct 28;3(10):e3541. doi: 10.1371/journal.pone.0003541 (PMC2568817; doi:10.1371/journal.pone.0003541)
Supplement: Table S2 — Results from Microarray Expression Analyses (2.68 MB DOC) [file pone.0003541.s002.doc]

**Supplementary Table S2. Results from Microarray Expression Analyses.**

| **GenBank locus tag; GenBank GI ; GenBank Gene ID; predicted function** | **Fold difference between OHHL+/OHHL- (quantitative PCR validation)** | **PPDE(p)** |
| --- | --- | --- |
| SG0586; GI:85058564; GeneID:3867700; hypothetical protein | 10.6 (5.83) | 1.000 |
| SG0585; GI:85058563; GeneID:3867699; hypothetical protein | 7.82 | 1.000 |
| SG0284; GI:85058262; GeneID:3868591; N-acylhomoserine lactone synthase | 4.55 (7.46) | 1.000 |
| SGP1_0024; GI:85060435; GeneID:3866563; hypothetical protein | 3.77 | 1.000 |
| SGP1_0023; GI:85060434; GeneID:3866562; hypothetical protein | 3.13 | 1.000 |
| SGP1_0021; GI:85060432; GeneID:3866560; putative TraE | 3.07 | 1.000 |
| SGP1_0022; GI:85060433; GeneID:3866561; TraL | 2.91 (2.38) | 1.000 |
| SGP1_0020; GI:85060431; GeneID:3866559; hypothetical protein | 2.79 | 1.000 |
| SGP1_0051; GI:85060462; GeneID:3866536; small heat shock protein | 2.45 | 0.985 |
| SG0610; GI:85058588; GeneID:3867584; putative xylitol dehydrogenase | 2.37 (2.98) | 1.000 |
| SG0614; GI:85058592; GeneID:3867588; sugar ABC transporter periplasmic component | 2.27 | 1.000 |
| SG0611; GI:85058589; GeneID:3867585; xylulose kinase | 2.22 | 1.000 |
| SG0612; GI:85058590; GeneID:3867586; sugar ABC transporter ATP-binding component | 2.22 | 1.000 |
| SGP1_0018; GI:85060429; GeneID:3866557; hypothetical protein | 2.19 | 1.000 |
| SG1553; GI:85059531; GeneID:3867193; hypothetical protein | 2.16 | 1.000 |
| SGP1_0019; GI:85060430; GeneID:3866558; hypothetical protein | 2.12 | 1.000 |
| SG0613; GI:85058591; GeneID:3867587; sugar ABC transporter permease component | 2.04 | 1.000 |
| SGP1_0017; GI:85060428; GeneID:3866556; hypothetical protein | 2.01 | 1.000 |
| SGP1_0045; GI:85060456; GeneID:3866584; achromobactin biosynthetic and transport gene | 1.99 (2.13) | 1.000 |
| SG1517; GI:85059495; GeneID:3868243; iron ABC transporter ATP-binding component | 1.95 | 1.000 |
| SG2101; GI:85060079; GeneID:3866476; catalase | 1.94 (2.16) | 1.000 |
| SG1904; GI:85059882; GeneID:3867777; hypothetical protein | 1.92 | 1.000 |
| SGP1_0046; GI:85060457; GeneID:3866585; aminobutyrate aminotransferase | 1.88 | 1.000 |
| SG0947; GI:85058925; GeneID:3868434; hypothetical protein | 1.83 | 1.000 |
| SG0745; GI:85058723; GeneID:3867722; putative phage endonuclease | 1.77 | 1.000 |
| SG0684; GI:85058662; GeneID:3868151; acriflavin efflux protein AcrB | 1.77 | 1.000 |
| SG1827; GI:85059805; GeneID:3866654; hypothetical protein | 1.76 | 1.000 |
| SG1516; GI:85059494; GeneID:3868242; iron ABC transporter periplasmic component | 1.75 | 1.000 |
| SG0872; GI:85058850; GeneID:3868320; succinate dehydrogenase cytochrome b-556 | 1.72 | 1.000 |
| SGP1_0015; GI:85060426; GeneID:3866554; hypothetical protein | 1.69 | 1.000 |
| SG1168; GI:85059146; GeneID:3866238; hypothetical protein | 1.69 | 1.000 |
| SG0692; GI:85058670; GeneID:3868609; heat shock protein HtpG | 1.68 | 1.000 |
| SG0584; GI:85058562; GeneID:3867698; heat shock protein | 1.67 | 1.000 |
| SG1286; GI:85059264; GeneID:3866396; putative secreted effector protein | 1.67 | 1.000 |
| SG0743; GI:85058721; GeneID:3867720; hypothetical protein | 1.67 | 1.000 |
| SG1518; GI:85059496; GeneID:3868244; iron ABC transporter permease component | 1.66 | 1.000 |
| SGP1_0016; GI:85060427; GeneID:3866555; putative TraU | 1.65 | 1.000 |
| SG0305; GI:85058283; GeneID:3868573; chaperonin GroES | 1.64 | 1.000 |
| SG1348; GI:85059326; GeneID:3866419; hypothetical protein | 1.63 | 1.000 |
| SGP1_0044; GI:85060455; GeneID:3866583; achromobactin biosynthetic and transport gene | 1.62 | 1.000 |
| SG0871; GI:85058849; GeneID:3868319; citrate synthase | 1.62 | 1.000 |
| SG2134; GI:85060112; GeneID:3867295; hypothetical protein | 1.61 | 1.000 |
| SG1289; GI:85059267; GeneID:3866399; translocation machinery component | 1.60 | 1.000 |
| SG1853; GI:85059831; GeneID:3867051; putative cation transport ATPase | 1.59 | 0.999 |
| SG0409; GI:85058387; GeneID:3867578; chaperone protein DnaK | 1.58 | 1.000 |
| SG1467; GI:85059445; GeneID:3866892; hypothetical protein | 1.57 | 1.000 |
| SG1556; GI:85059534; GeneID:3867196; putative phage terminase large subunit | 1.56 | 1.000 |
| SG2325; GI:85060303; GeneID:3867310; hypothetical protein | 1.54 | 1.000 |
| SG1243; GI:85059221; GeneID:3866766; proline dehydrogenase | 1.54 | 1.000 |
| SG1285; GI:85059263; GeneID:3866395; hypothetical protein | 1.53 | 1.000 |
| SG1288; GI:85059266; GeneID:3866398; putative translocation machinery component | 1.52 | 1.000 |
| SG1493; GI:85059471; GeneID:3868180; hypothetical protein | 1.51 | 1.000 |
| SG1035; GI:85059013; GeneID:3867221; putative phage integrase | 1.51 | 1.000 |
| SG1387; GI:85059365; GeneID:3867262; phage lysozyme lysis protein | 1.50 | 0.999 |
| SG0073; GI:85058051; GeneID:3867497; oligopeptidase A | 1.48 | 1.000 |
| SGP1_0043; GI:85060454; GeneID:3866582; achromobactin biosynthetic and transport gene | 1.47 | 1.000 |
| SG1555; GI:85059533; GeneID:3867195; putative phage minor head protein | 1.47 | 1.000 |
| SG1290; GI:85059268; GeneID:3866400; putative secreted effector protein | 1.46 | 1.000 |
| SG1522; GI:85059500; GeneID:3868248; hypothetical protein | 1.46 | 1.000 |
| SG0516; GI:85058494; GeneID:3868217; sulfite reductase (NADPH) flavoprotein | 1.45 | 1.000 |
| SG0874; GI:85058852; GeneID:3868322; succinate dehydrogenase flavoprotein subunit | 1.45 | 1.000 |
| SG1554; GI:85059532; GeneID:3867194; hypothetical protein | 1.45 | 1.000 |
| SG0877; GI:85058855; GeneID:3868325; 2-oxoglutarate dehydrogenase E2 component | 1.45 | 1.000 |
| SG1468; GI:85059446; GeneID:3866893; hypothetical protein | 1.45 | 1.000 |
| SG1584; GI:85059562; GeneID:3867879; ribonucleoside-diphosphate reductase 1 alpha | 1.44 | 1.000 |
| SG0782; GI:85058760; GeneID:3868049; putative N-acetylmuramoyl-L-alanine amidase | 1.44 | 1.000 |
| SG0673; GI:85058651; GeneID:3868140; ATP-dependent protease Lon | 1.44 | 1.000 |
| SG0609; GI:85058587; GeneID:3867583; hypothetical protein | 1.44 | 1.000 |
| SG1609; GI:85059587; GeneID:3867787; putative glutathione S-transferase | 1.44 | 1.000 |
| SG2135; GI:85060113; GeneID:3867296; conserced hypothetical protein | 1.44 | 1.000 |
| SG2133; GI:85060111; GeneID:3867294; hydantoinase | 1.44 | 1.000 |
| SG1637; GI:85059615; GeneID:3867160; heme exporter protein A | 1.43 | 0.998 |
| SG0897; GI:85058875; GeneID:3868345; UDP-D-galactose 4-epimerase GalE | 1.43 | 1.000 |
| SGP2_0010; GI:85060476; GeneID:3866611; putative hydantoinase | 1.42 | 1.000 |
| SG1521; GI:85059499; GeneID:3868247; hypothetical protein | 1.42 | 0.992 |
| SG0873; GI:85058851; GeneID:3868321; succinate dehydrogenase hydrophobic subunit | 1.42 | 1.000 |
| SG0608; GI:85058586; GeneID:3866676; xylitol repressor | 1.41 | 1.000 |
| SG0748; GI:85058726; GeneID:3867725; hypothetical protein | 1.41 | 1.000 |
| SG2169; GI:85060147; GeneID:3867943; heat shock protein | 1.41 | 1.000 |
| SG1828; GI:85059806; GeneID:3866655; hypothetical protein | 1.41 | 1.000 |
| SG0008; GI:85057986; GeneID:3867665; heat shock protein A | 1.41 | 1.000 |
| SG0685; GI:85058663; GeneID:3868152; acriflavin efflux protein AcrA | 1.40 | 1.000 |
| SG1585; GI:85059563; GeneID:3867880; ribonucleoside-diphosphate reductase 1 beta | 1.40 | 1.000 |
| SG1586; GI:85059564; GeneID:3867881; hypothetical protein | 1.40 | 1.000 |
| SG1525; GI:85059503; GeneID:3867224; major facilitator family transport protein | 1.39 | 1.000 |
| SG1965; GI:85059943; GeneID:3866223; hypothetical protein | 1.39 | 1.000 |
| SG0939; GI:85058917; GeneID:3868387; phage lysozyme lysis protein | 1.39 | 1.000 |
| SG0071; GI:85058049; GeneID:3867495; cytosine transport protein | 1.39 | 0.999 |
| SG0306; GI:85058284; GeneID:3868574; chaperonin GroEL | 1.39 | 1.000 |
| SG1327; GI:85059305; GeneID:3867522; PTS system mannose-specific IIAB component ManX | 1.38 | 1.000 |
| SG1034; GI:85059012; GeneID:3867220; hypothetical protein | 1.38 | 0.988 |
| SG1519; GI:85059497; GeneID:3868245; iron ABC transporter permease component | 1.38 | 1.000 |
| SG0410; GI:85058388; GeneID:3867579; chaperone protein DnaJ | 1.38 | 1.000 |
| SG0742; GI:85058720; GeneID:3867719; hypothetical protein | 1.37 | 1.000 |
| SG1349; GI:85059327; GeneID:3866420; hypothetical protein | 1.37 | 1.000 |
| SG0290; GI:85058268; GeneID:3868597; putative glycosyltransferase | 1.37 | 0.999 |
| SG2078; GI:85060056; GeneID:3868109; type III secretion apparatus | 1.36 | 1.000 |
| SG1821; GI:85059799; GeneID:3866648; hypothetical protein | 1.36 | 0.999 |
| SG1287; GI:85059265; GeneID:3866397; putative type III secretion chaperone | 1.36 | 1.000 |
| SGP1_0014; GI:85060425; GeneID:3866553; hypothetical protein | 1.36 | 1.000 |
| SG0962; GI:85058940; GeneID:3867089; mgl repressor and galactose operon inducer | 1.35 | 0.998 |
| SG0875; GI:85058853; GeneID:3868323; succinate dehydrogenase iron-sulfur subunit | 1.35 | 0.999 |
| SG1496; GI:85059474; GeneID:3868183; hypothetical protein | 1.34 | 0.998 |
| SG1292; GI:85059270; GeneID:3867107; putative type III secretion apparatus | 1.34 | 1.000 |
| SG2168; GI:85060146; GeneID:3867942; heat shock protein | 1.34 | 1.000 |
| SG0774; GI:85058752; GeneID:3868041; hypothetical protein | 1.33 | 0.984 |
| SG0878; GI:85058856; GeneID:3868326; succinyl-CoA synthase beta subunit | 1.33 | 1.000 |
| SG1279; GI:85059257; GeneID:3866389; putative two-component response regulator | 1.33 | 1.000 |
| SG0408; GI:85058386; GeneID:3867577; alanine-sodium transport protein | 1.33 | 0.947 |
| SG0517; GI:85058495; GeneID:3868218; sulfite reductase (NADPH) hemoprotein | 1.33 | 1.000 |
| SG2280; GI:85060258; GeneID:3866422; bacterioferritin | 1.33 | 0.998 |
| SG2123; GI:85060101; GeneID:3867284; hypothetical protein | 1.32 | 1.000 |
| SG1227; GI:85059205; GeneID:3868680; phage repressor | 1.32 | 1.000 |
| SG0876; GI:85058854; GeneID:3868324; 2-oxoglutarate dehydrogenase E1 component | 1.32 | 1.000 |
| SG2079; GI:85060057; GeneID:3868110; type III secretion apparatus | 1.32 | 1.000 |
| SG0017; GI:85057995; GeneID:3867674; manganese superoxide dismutase | 1.32 | 0.975 |
| SG1479; GI:85059457; GeneID:3866904; putative cytosine transport protein | 1.32 | 0.998 |
| SG1714; GI:85059692; GeneID:3866853; transaldolase A | 1.31 | 0.999 |
| SG1042; GI:85059020; GeneID:3866954; hypothetical protein | 1.31 | 0.997 |
| SG0518; GI:85058496; GeneID:3868219; 3'-phosphoadenosine 5'-phosphosulfate | 1.30 | 0.998 |
| SG0642; GI:85058620; GeneID:3868461; putative peroxidase | 1.30 | 0.997 |
| SG2122; GI:85060100; GeneID:3866263; acetyl-coenzyme A synthase | 1.30 | 0.999 |
| SG1706; GI:85059684; GeneID:3866452; sulfate ABC transporter ATP-binding component | 1.30 | 0.998 |
| SG0094; GI:85058072; GeneID:3867754; phage lysozyme lysis protein | 1.30 | 0.999 |
| SG0682; GI:85058660; GeneID:3868149; hypothetical protein | 1.29 | 1.000 |
| SG1498; GI:85059476; GeneID:3868185; thiol peroxidase | 1.29 | 1.000 |
| SG1132; GI:85059110; GeneID:3867560; exonuclease I | 1.29 | 1.000 |
| SG2214; GI:85060192; GeneID:3868759; hypothetical protein | 1.28 | 1.000 |
| SG0504; GI:85058482; GeneID:3868283; deoxyguanosine triphosphate triphosphohydrolase | 1.28 | 0.996 |
| SG1865; GI:85059843; GeneID:3867063; osmotically inducible lipoprotein OsmE | 1.27 | 0.996 |
| SG0469; GI:85058447; GeneID:3868209; dihydrolipoamide dehydrogenase | 1.27 | 0.999 |
| SG1145; GI:85059123; GeneID:3867136; hypothetical protein | 1.27 | 1.000 |
| SG0879; GI:85058857; GeneID:3868327; succinyl-CoA synthase alpha subunit | 1.27 | 0.991 |
| SG1798; GI:85059776; GeneID:3866801; heat shock protein GrpE | 1.27 | 0.992 |
| SG1360; GI:85059338; GeneID:3867332; hypothetical protein | 1.26 | 0.992 |
| SG1205; GI:85059183; GeneID:3866844; hypothetical protein | 1.26 | 0.997 |
| SGP2_0009; GI:85060475; GeneID:3866610; hypothetical protein | 1.26 | 0.945 |
| SG1138; GI:85059116; GeneID:3867129; hypothetical protein | 1.26 | 0.977 |
| SG1755; GI:85059733; GeneID:3866914; putative acetyltransferase | 1.26 | 0.998 |
| SG0700; GI:85058678; GeneID:3868617; isocitrate dehydrogenase | 1.25 | 1.000 |
| SG0709; GI:85058687; GeneID:3868729; phage DNA-invertase | 1.25 | 1.000 |
| SG2094; GI:85060072; GeneID:3866469; putative transcriptional repressor | 1.25 | 0.998 |
| SG2162; GI:85060140; GeneID:3867936; 5,10-methylenetetrahydrofolate reductase | 1.25 | 0.998 |
| SG2080; GI:85060058; GeneID:3868111; putative type III secretion chaperone | 1.25 | 0.996 |
| SG1291; GI:85059269; GeneID:3867106; type III secretion chaperone | 1.25 | 0.999 |
| SG0020; GI:85057998; GeneID:3867677; transcriptional regulator | 1.25 | 0.937 |
| SG0728; GI:85058706; GeneID:3867705; phage lysozyme lysis protein | 1.25 | 1.000 |
| SG1488; GI:85059466; GeneID:3868175; pyridine nucleotide transhydrogenase alpha | 1.25 | 1.000 |
| GI:125470047; GI:125470047; gp32; possible endopeptidase Rz; similar to gp13 of pSG3 | 1.24 | 1.000 |
| SG1375; GI:85059353; GeneID:3867250; oligopeptide ABC transporter permease component | 1.24 | 1.000 |
| SG2220; GI:85060198; GeneID:3868804; putative transcription regulator | 1.24 | 0.988 |
| SG0982; GI:85058960; GeneID:3866875; hypothetical protein | 1.24 | 0.992 |
| SG1137; GI:85059115; GeneID:3867128; 3-methyl-adenine DNA glycosylase I | 1.24 | 0.948 |
| SG0477; GI:85058455; GeneID:3868691; aconitate hydratase 2 | 1.24 | 0.998 |
| SG0427; GI:85058405; GeneID:3867538; organic solvent tolerance protein | 1.24 | 1.000 |
| SG0250; GI:85058228; GeneID:3868302; arsenate reductase | 1.24 | 0.998 |
| SG0057; GI:85058035; GeneID:3867442; flagellar hook-associated protein 2 FliD | 1.24 | 0.998 |
| SG2158; GI:85060136; GeneID:3868267; hydrogen peroxide-inducible regulon activator | 1.24 | 0.988 |
| SG0671; GI:85058649; GeneID:3868138; ATP-dependent Clp protease proteolytic subunit | 1.24 | 0.995 |
| SG1311; GI:85059289; GeneID:3867506; hypothetical protein | 1.23 | 0.958 |
| SG1241; GI:85059219; GeneID:3866764; trehalose-6-phosphate synthase | 1.23 | 1.000 |
| SG1600; GI:85059578; GeneID:3867895; NADH dehydrogenase I subunit B | 1.23 | 0.994 |
| SGP1_0042; GI:85060453; GeneID:3866581; achromobactin biosynthetic and transport gene | 1.23 | 0.999 |
| SG0522; GI:85058500; GeneID:3868223; ATP-sulfurylase subunit 1 | 1.23 | 0.992 |
| SGP1_0047; GI:85060458; GeneID:3866586; transposase | 1.23 | 0.994 |
| SG1743; GI:85059721; GeneID:3866488; putative transport system permease protein | 1.23 | 0.997 |
| SG0811; GI:85058789; GeneID:3868020; putative tRNA-thiotransferase | 1.23 | 0.989 |
| SG1345; GI:85059323; GeneID:3866416; hypothetical protein | 1.22 | 0.997 |
| SG2239; GI:85060217; GeneID:3867367; putative molybdopterin-guanine dinucleotide | 1.22 | 0.999 |
| SG1562; GI:85059540; GeneID:3867202; death-on-curing protein | 1.22 | 0.819 |
| SG0103; GI:85058081; GeneID:3867976; hypothetical protein | 1.22 | 0.989 |
| SG0298; GI:85058276; GeneID:3868566; hypothetical protein | 1.22 | 0.997 |
| SG1280; GI:85059258; GeneID:3866390; putative two-component sensor kinase | 1.22 | 0.999 |
| SG0523; GI:85058501; GeneID:3868224; adenosine 5-phosphosulfate kinase | 1.22 | 0.971 |
| SG2361; GI:85060339; GeneID:3866818; hypothetical protein | 1.22 | 0.999 |
| SG1373; GI:85059351; GeneID:3867248; oligopeptide ABC transporter periplasmic | 1.22 | 0.998 |
| SG0014; GI:85057992; GeneID:3867671; PTS system mannitol-specific IIABC component | 1.22 | 0.999 |
| SG1709; GI:85059687; GeneID:3866455; sulfate ABC transporter periplasmic component | 1.21 | 0.983 |
| SG0297; GI:85058275; GeneID:3868565; hypothetical protein | 1.21 | 0.996 |
| SG0359; GI:85058337; GeneID:3867471; arginine repressor | 1.21 | 0.971 |
| SG0155; GI:85058133; GeneID:3868521; hypothetical protein | 1.21 | 0.876 |
| SG0985; GI:85058963; GeneID:3866878; putative glycosyltransferase | 1.21 | 0.968 |
| SG0800; GI:85058778; GeneID:3868483; nicotinate-nucleotide adenylyltransferase | 1.21 | 0.998 |
| SG0986; GI:85058964; GeneID:3866879; putative extracellular polysaccharide | 1.20 | 0.985 |
| SG1643; GI:85059621; GeneID:3867821; hypothetical protein | 1.20 | 0.976 |
| SGP1_0003; GI:85060414; GeneID:3866542; hypothetical protein | 1.20 | 0.973 |
| SGP1_0041; GI:85060452; GeneID:3866580; achromobactin biosynthetic and transport gene | 1.20 | 0.994 |
| SG1106; GI:85059084; GeneID:3868716; thioredoxin reductase | 1.20 | 0.990 |
| SG1987; GI:85059965; GeneID:3867068; putative phosphatase | 1.20 | 0.996 |
| SG2077; GI:85060055; GeneID:3868108; type III secretion apparatus | 1.20 | 0.999 |
| SGP3_0004; GI:85060494; GeneID:3866615; hypothetical protein | 1.20 | 0.990 |
| SG0785; GI:85058763; GeneID:3868468; hypothetical protein | 1.20 | 0.990 |
| SG0848; GI:85058826; GeneID:3867806; hypothetical protein | 1.20 | 0.974 |
| SG2197; GI:85060175; GeneID:3867005; putative lipopolysaccharide glycosyltransferase | 1.20 | 0.996 |
| SG1440; GI:85059418; GeneID:3866943; hypothetical protein | 1.20 | 0.950 |
| SG1069; GI:85059047; GeneID:3866709; hypothetical protein | 1.20 | 0.998 |
| SG0794; GI:85058772; GeneID:3868477; hypothetical protein | 1.20 | 0.984 |
| SG0693; GI:85058671; GeneID:3868610; adenylate kinase | 1.19 | 0.995 |
| SGP1_0040; GI:85060451; GeneID:3866579; achromobactin biosynthetic and transport gene | 1.19 | 0.989 |
| SG0714; GI:85058692; GeneID:3868734; hypothetical protein | 1.19 | 0.996 |
| SG1884; GI:85059862; GeneID:3866358; ethanolamine utilization protein EutN | 1.19 | 0.994 |
| SG0265; GI:85058243; GeneID:3867905; hypothetical protein | 1.19 | 0.992 |
| SG1018; GI:85058996; GeneID:3867204; dihydroorotate dehydrogenase | 1.19 | 0.992 |
| SG1999; GI:85059977; GeneID:3867080; hypothetical protein | 1.19 | 0.997 |
| SG1122; GI:85059100; GeneID:3867550; gluconate-6-phosphate dehydrogenase | 1.19 | 0.997 |
| SG2095; GI:85060073; GeneID:3866470; putative antibiotic biosynthesis protein | 1.19 | 0.987 |
| SG0665; GI:85058643; GeneID:3868011; cytochrome o ubiquinol oxidase subunit III | 1.19 | 0.977 |
| SG0666; GI:85058644; GeneID:3868012; cytochrome o ubiquinol oxidase subunit I | 1.19 | 0.992 |
| SG1326; GI:85059304; GeneID:3867521; PTS system mannose-specific IIC component ManY | 1.19 | 0.997 |
| SG0815; GI:85058793; GeneID:3868024; N-acetylglucosamine-6-phosphate deacetylase | 1.19 | 0.999 |
| SG1251; GI:85059229; GeneID:3866774; copper homeostasis protein | 1.19 | 0.914 |
| SG1303; GI:85059281; GeneID:3867118; putative type III secretion apparatus | 1.19 | 0.998 |
| SGP1_0039; GI:85060450; GeneID:3866578; achromobactin biosynthetic and transport gene | 1.18 | 0.978 |
| SG0340; GI:85058318; GeneID:3868119; hypothetical protein | 1.18 | 0.987 |
| SG2230; GI:85060208; GeneID:3868814; glutamine synthase | 1.18 | 0.994 |
| GI:125470045; GI:125470045; gp30; possible untranslated holin; similar to gp15 of | 1.18 | 0.995 |
| SG0978; GI:85058956; GeneID:3867105; amylovoran export outer membrane protein AmsH | 1.18 | 0.992 |
| SG1610; GI:85059588; GeneID:3867788; putative sugar nucleotide epimerase | 1.18 | 0.998 |
| SG1155; GI:85059133; GeneID:3866225; hypothetical protein | 1.18 | 0.835 |
| SG1997; GI:85059975; GeneID:3867078; hypothetical protein | 1.18 | 0.997 |
| SG1333; GI:85059311; GeneID:3866404; ribonuclease D | 1.18 | 0.988 |
| SG1092; GI:85059070; GeneID:3868169; hypothetical protein | 1.18 | 0.996 |
| SG1232; GI:85059210; GeneID:3868685; hypothetical protein | 1.18 | 0.995 |
| SG0243; GI:85058221; GeneID:3868295; transposase | 1.18 | 0.997 |
| SG1635; GI:85059613; GeneID:3867158; heme exporter protein D | 1.18 | 0.992 |
| SG0148; GI:85058126; GeneID:3868514; hypothetical protein | 1.18 | 0.993 |
| SG0905; GI:85058883; GeneID:3868353; biotin synthesis protein BioC | 1.18 | 0.987 |
| SG1589; GI:85059567; GeneID:3867884; NADH dehydrogenase I subunit N | 1.18 | 0.998 |
| SG1461; GI:85059439; GeneID:3867278; mannose-6-phosphate isomerase | 1.18 | 0.995 |
| SGP1_0026; GI:85060437; GeneID:3866565; hypothetical protein | 1.18 | 0.976 |
| SGP1_0013; GI:85060424; GeneID:3866552; hypothetical protein | 1.18 | 0.990 |
| SG1698; GI:85059676; GeneID:3866281; hypothetical protein | 1.18 | 0.989 |
| SG0011; GI:85057989; GeneID:3867668; glycyl-tRNA synthase alpha subunit | 1.18 | 0.971 |
| SG0841; GI:85058819; GeneID:3868783; hypothetical protein | 1.17 | 0.934 |
| SG0668; GI:85058646; GeneID:3868135; putative lipoprotein | 1.17 | 0.997 |
| SG0901; GI:85058879; GeneID:3868349; hypothetical protein | 1.17 | 0.996 |
| SG0984; GI:85058962; GeneID:3866877; hypothetical protein | 1.17 | 0.952 |
| SG0894; GI:85058872; GeneID:3868342; phosphoglyceromutase 1 | 1.17 | 0.985 |
| SG0983; GI:85058961; GeneID:3866876; hypothetical protein | 1.17 | 0.884 |
| SG1480; GI:85059458; GeneID:3866905; hypothetical protein | 1.17 | 0.942 |
| SG0737; GI:85058715; GeneID:3867714; putative transport protein | 1.17 | 0.964 |
| SG1051; GI:85059029; GeneID:3866963; dihydroorotase | 1.17 | 0.996 |
| SG1924; GI:85059902; GeneID:3867657; hypothetical protein | 1.17 | 0.991 |
| SG0732; GI:85058710; GeneID:3867709; hypothetical protein | 1.17 | 0.954 |
| SG1222; GI:85059200; GeneID:3868675; hypothetical protein | 1.17 | 0.983 |
| SG0453; GI:85058431; GeneID:3868193; cell division protein FtsZ | 1.17 | 0.990 |
| SG0747; GI:85058725; GeneID:3867724; putative phage lysozyme lysis protein | 1.17 | 0.982 |
| SG0068; GI:85058046; GeneID:3867492; glutathione reductase | 1.17 | 0.981 |
| SG0981; GI:85058959; GeneID:3866874; amylovoran biosynthesis glycosyl transferase | 1.17 | 0.903 |
| SG1704; GI:85059682; GeneID:3866450; putative two-component system response | 1.17 | 0.976 |
| SG0242; GI:85058220; GeneID:3868294; hypothetical protein | 1.17 | 0.985 |
| SG1381; GI:85059359; GeneID:3867256; membrane protein TonB | 1.17 | 0.978 |
| SG1966; GI:85059944; GeneID:3866224; putative phage replication protein | 1.17 | 0.986 |
| SG1338; GI:85059316; GeneID:3866409; putative isomerase | 1.17 | 0.985 |
| SG1826; GI:85059804; GeneID:3866653; putative transcriptional regulator | 1.17 | 0.973 |
| GI:125470038; GI:125470038; gp23; hypothetical protein; similar to gp23 of pSG3 | 1.17 | 0.991 |
| SG0620; GI:85058598; GeneID:3867594; hypothetical protein | 1.16 | 0.944 |
| SG1765; GI:85059743; GeneID:3866924; putative thiosulfate sulfurtransferase | 1.16 | 0.987 |
| SG0932; GI:85058910; GeneID:3868380; hypothetical protein | 1.16 | 0.998 |
| SG1866; GI:85059844; GeneID:3867064; NH3-dependent NAD synthase | 1.16 | 0.984 |
| SG1231; GI:85059209; GeneID:3868684; hypothetical protein | 1.16 | 0.959 |
| SG0664; GI:85058642; GeneID:3868010; cytochrome o ubiquinol oxidase subunit IV | 1.16 | 0.980 |
| SG1070; GI:85059048; GeneID:3866710; hypothetical protein | 1.16 | 0.992 |
| SG0266; GI:85058244; GeneID:3867906; outer membrane protein TolC precursor | 1.16 | 0.990 |
| SG2329; GI:85060307; GeneID:3867314; aerobic glycerol-3-phosphate dehydrogenase GlpD | 1.16 | 0.944 |
| SG0058; GI:85058036; GeneID:3867443; flagellin FliC | 1.16 (1.09) | 0.991 |
| SG1727; GI:85059705; GeneID:3866866; glycine cleavage system transcriptional | 1.16 | 0.982 |
| SG0474; GI:85058452; GeneID:3868688; hypothetical protein | 1.16 | 0.983 |
| SG0056; GI:85058034; GeneID:3867441; flagellar protein FliS | 1.16 | 0.970 |
| SG1317; GI:85059295; GeneID:3867512; heat shock protein | 1.16 | 0.988 |
| SG2317; GI:85060295; GeneID:3866323; phosphoenolpyruvate carboxykinase | 1.15 | 0.913 |
| SG1130; GI:85059108; GeneID:3867558; ATP phosphoribosyltransferase HisG | 1.15 | 0.982 |
| SG2044; GI:85060022; GeneID:3866755; hypothetical protein | 1.15 | 0.998 |
| SG0980; GI:85058958; GeneID:3866873; putative tyrosine-protein kinase | 1.15 | 0.965 |
| SG0486; GI:85058464; GeneID:3868700; aspartate 1-decarboxylase precursor | 1.15 | 0.970 |
| SG1484; GI:85059462; GeneID:3866909; fumarate nitrate reduction regulator | 1.15 | 0.986 |
| SG1549; GI:85059527; GeneID:3867189; putative right origin-binding protein | 1.15 | 0.933 |
| SG0426; GI:85058404; GeneID:3867537; survival protein SurA precursor | 1.15 | 0.989 |
| SG1680; GI:85059658; GeneID:3868544; putative sulfate transport protein | 1.15 | 0.969 |
| GI:125470064; GI:125470064; gp52; possible holin; similar to gp30 of pSOG3 and to | 1.15 | 0.987 |
| SG1935; GI:85059913; GeneID:3867847; outer membrane protein | 1.15 | 0.975 |
| SG1634; GI:85059612; GeneID:3867157; thiol:disulfide interchange protein DsbE | 1.15 | 0.946 |
| GI:125470036; GI:125470036; gp20; possible ExoVIII (RecE) protein; similar to | 1.15 | 0.992 |
| SG0570; GI:85058548; GeneID:3867684; type III secretion apparatus SpaQ | 1.15 | 0.937 |
| SG0686; GI:85058664; GeneID:3868153; AcrAB operon repressor AcrR | 1.15 | 0.978 |
| SG1389; GI:85059367; GeneID:3867029; putative phage terminase large subunit | 1.15 | 0.990 |
| SG1171; GI:85059149; GeneID:3866241; excinuclease ABC subunit C | 1.15 | 0.969 |
| SG0493; GI:85058471; GeneID:3868272; sugar fermentation stimulation protein | 1.15 | 0.957 |
| SG2409; GI:85060387; GeneID:3868408; ATP synthase subunit C | 1.15 | 0.979 |
| SG0022; GI:85058000; GeneID:3867679; flagellar transcriptional activator FlhC | 1.15 | 0.974 |
| SG0101; GI:85058079; GeneID:3867761; hypothetical protein | 1.15 | 0.965 |
| SG1573; GI:85059551; GeneID:3866686; hypothetical protein | 1.15 | 0.994 |
| SG1636; GI:85059614; GeneID:3867159; heme exporter protein C | 1.15 | 0.983 |
| SG1815; GI:85059793; GeneID:3866642; hypothetical protein | 1.14 | 0.981 |
| SG0778; GI:85058756; GeneID:3868045; hypothetical protein | 1.14 | 0.990 |
| SG0765; GI:85058743; GeneID:3868032; hypothetical protein | 1.14 | 0.860 |
| SG0707; GI:85058685; GeneID:3868727; hypothetical protein | 1.14 | 0.996 |
| SG1174; GI:85059152; GeneID:3868545; hypothetical protein | 1.14 | 0.859 |
| SG0903; GI:85058881; GeneID:3868351; biotin synthase BioB | 1.14 | 0.971 |
| SG1792; GI:85059770; GeneID:3866795; sigma-E factor negative regulator | 1.14 | 0.922 |
| SG2099; GI:85060077; GeneID:3866474; hypothetical protein | 1.14 | 0.904 |
| SG1557; GI:85059535; GeneID:3867197; hypothetical protein | 1.14 | 0.967 |
| SG1790; GI:85059768; GeneID:3866534; sigma-E factor regulator | 1.14 | 0.914 |
| SG1962; GI:85059940; GeneID:3866220; hypothetical protein | 1.14 | 0.982 |
| SG0195; GI:85058173; GeneID:3866634; phosphocarrier protein NPr | 1.14 | 0.985 |
| SG2240; GI:85060218; GeneID:3866717; putative transferase | 1.14 | 0.967 |
| SG1594; GI:85059572; GeneID:3867889; NADH dehydrogenase I subunit I | 1.14 | 0.941 |
| SG0531; GI:85058509; GeneID:3868647; DNA mismatch repair protein MutS | 1.14 | 0.967 |
| SG1882; GI:85059860; GeneID:3866356; hypothetical protein | 1.14 | 0.983 |
| SG1446; GI:85059424; GeneID:3866949; hypothetical protein | 1.14 | 0.973 |
| SG0979; GI:85058957; GeneID:3866872; putative tyrosine-phosphatase | 1.14 | 0.913 |
| SG0535; GI:85058513; GeneID:3868651; amino-acid ABC transporter permease component | 1.14 | 0.927 |
| SG1460; GI:85059438; GeneID:3867277; hypothetical protein | 1.14 | 0.912 |
| SG1374; GI:85059352; GeneID:3867249; oligopeptide ABC transporter permease component | 1.14 | 0.936 |
| SG1376; GI:85059354; GeneID:3867251; oligopeptide ABC transporter ATP-binding | 1.14 | 0.976 |
| SG1038; GI:85059016; GeneID:3866950; putative acylphosphatase | 1.14 | 0.961 |
| SG0968; GI:85058946; GeneID:3867095; cytidine deaminase | 1.14 | 0.916 |
| SG1658; GI:85059636; GeneID:3867836; hypothetical protein | 1.14 | 0.964 |
| SG0349; GI:85058327; GeneID:3868128; FKBP-type peptidyl-prolyl cis-trans isomerase | 1.14 | 0.922 |
| SG0806; GI:85058784; GeneID:3868015; glutamate-aspartate ABC transporter periplasmic | 1.14 | 0.856 |
| SG1325; GI:85059303; GeneID:3867520; PTS system mannose-specific IID component ManZ | 1.13 | 0.961 |
| SG1140; GI:85059118; GeneID:3867131; hypothetical protein | 1.13 | 0.763 |
| SG2263; GI:85060241; GeneID:3866288; 50S ribosomal protein L6 | 1.13 | 0.867 |
| SG1401; GI:85059379; GeneID:3867041; anthranilate synthase component II TrpD | 1.13 | 0.833 |
| SG2022; GI:85060000; GeneID:3868801; glutathione synthase | 1.13 | 0.982 |
| SG2246; GI:85060224; GeneID:3866723; polypeptide deformylase | 1.13 | 0.944 |
| SG1514; GI:85059492; GeneID:3868240; exoribonuclease II | 1.13 | 0.978 |
| SG1745; GI:85059723; GeneID:3866490; putative PTS system galactitol-specific IIB | 1.13 | 0.985 |
| SG1820; GI:85059798; GeneID:3866647; phage terminase large subunit | 1.13 | 0.965 |
| GI:125470050; GI:125470050; gp36; possible Tn21-like resolvase; similar to gp08 of | 1.13 | 0.957 |
| SG1489; GI:85059467; GeneID:3868176; hypothetical protein | 1.13 | 0.972 |
| SG0407; GI:85058385; GeneID:3867576; hypothetical protein | 1.13 | 0.880 |
| SG0922; GI:85058900; GeneID:3868370; serine transport protein | 1.13 | 0.964 |
| SG2049; GI:85060027; GeneID:3866501; hypothetical protein | 1.13 | 0.982 |
| SG1331; GI:85059309; GeneID:3866402; hypothetical protein | 1.13 | 0.946 |
| SG2322; GI:85060300; GeneID:3867307; ferrous iron transport protein A | 1.13 | 0.879 |
| SG0865; GI:85058843; GeneID:3868313; negative modulator of replication initiation | 1.13 | 0.948 |
| SGP1_0053; GI:85060464; GeneID:3866538; hypothetical protein | 1.13 | 0.891 |
| SG1890; GI:85059868; GeneID:3867763; hypothetical protein | 1.13 | 0.889 |
| SG0854; GI:85058832; GeneID:3867812; putative phage baseplate protein | 1.12 | 0.874 |
| SGP1_0010; GI:85060421; GeneID:3866549; hypothetical protein | 1.12 | 0.982 |
| SG1707; GI:85059685; GeneID:3866453; sulfate ABC transporter permease component CysW | 1.12 | 0.733 |
| SG1845; GI:85059823; GeneID:3868638; conserved hypothteical protein | 1.12 | 0.930 |
| SG2083; GI:85060061; GeneID:3868114; type III secretion apparatus SpaQ | 1.12 | 0.934 |
| SG0969; GI:85058947; GeneID:3867096; hypothetical protein | 1.12 | 0.932 |
| SG1716; GI:85059694; GeneID:3866855; hypothetical protein | 1.12 | 0.965 |
| SG0694; GI:85058672; GeneID:3868611; ferrochelatase | 1.12 | 0.932 |
| SGP1_0005; GI:85060416; GeneID:3866544; hypothetical protein | 1.12 | 0.924 |
| SG1463; GI:85059441; GeneID:3867280; DNA replication terminus site-binding protein | 1.12 | 0.899 |
| SG1416; GI:85059394; GeneID:3866977; hypothetical protein | 1.12 | 0.955 |
| SG0902; GI:85058880; GeneID:3868350; adenosylmethionine-8-amino-7-oxononanoate | 1.12 | 0.934 |
| SG1240; GI:85059218; GeneID:3866763; trehalose-6-phosphate phophatase | 1.12 | 0.962 |
| SG1175; GI:85059153; GeneID:3868546; phage recombinase | 1.12 | 0.558 |
| SG1524; GI:85059502; GeneID:3868250; hypothetical protein | 1.12 | 0.920 |
| SG0247; GI:85058225; GeneID:3868299; hypothetical protein | 1.12 | 0.955 |
| SG1347; GI:85059325; GeneID:3866418; glyceraldehyde 3-phosphate dehydrogenase | 1.12 | 0.794 |
| SG1036; GI:85059014; GeneID:3867222; putative transport protein | 1.12 | 0.804 |
| SG0589; GI:85058567; GeneID:3866657; putative hydroxyacylglutathione hydrolase | 1.12 | 0.964 |
| SG0910; GI:85058888; GeneID:3868358; hypothetical protein | 1.12 | 0.951 |
| SG0282; GI:85058260; GeneID:3868589; biopolymer transport protein | 1.12 | 0.912 |
| SG1426; GI:85059404; GeneID:3866987; hypothetical protein | 1.12 | 0.887 |
| SGP2_0008; GI:85060474; GeneID:3866609; cytosine transporter protein | 1.12 | 0.675 |
| SG0749; GI:85058727; GeneID:3867726; phage terminase large subunit | 1.12 | 0.956 |
| SG1297; GI:85059275; GeneID:3867112; putative type III secretion apparatus | 1.12 | 0.938 |
| SG2151; GI:85060129; GeneID:3868260; putative gluconokinase | 1.12 | 0.845 |
| SG0566; GI:85058544; GeneID:3867618; type III secretion apparatus | 1.12 | 0.927 |
| SG2388; GI:85060366; GeneID:3867359; ATP-dependent RNA helicase | 1.12 | 0.964 |
| SG1843; GI:85059821; GeneID:3868636; putative formyl transferase | 1.12 | 0.846 |
| SGP1_0027; GI:85060438; GeneID:3866566; hypothetical protein | 1.12 | 0.944 |
| GI:125470037; GI:125470037; gp21; hypothetical protein | 1.12 | 0.864 |
| SG0741; GI:85058719; GeneID:3867718; hypothetical protein | 1.12 | 0.929 |
| SGP1_0011; GI:85060422; GeneID:3866550; hypothetical protein | 1.12 | 0.948 |
| SG1006; GI:85058984; GeneID:3866370; aspartate aminotransferase | 1.12 | 0.936 |
| SG0102; GI:85058080; GeneID:3867975; hypothetical protein | 1.12 | 0.914 |
| SG1220; GI:85059198; GeneID:3868673; hypothetical protein | 1.11 | 0.906 |
| SG1844; GI:85059822; GeneID:3868637; putative glycosyl transferase | 1.11 | 0.925 |
| SGtRNA50; GeneID:3867372; tRNA-Leu | 1.11 | 0.802 |
| SG0043; GI:85058021; GeneID:3867428; flagellar protein FliP | 1.11 | 0.944 |
| SG1666; GI:85059644; GeneID:3868530; hypothetical protein | 1.11 | 0.939 |
| SG1487; GI:85059465; GeneID:3868174; pyridine nucleotide transhydrogenase beta | 1.11 | 0.964 |
| SG1628; GI:85059606; GeneID:3867151; hypothetical protein | 1.11 | 0.957 |
| SG1435; GI:85059413; GeneID:3866938; putative selenocysteine lyase | 1.11 | 0.936 |
| SG0533; GI:85058511; GeneID:3868649; putative ABC transporter ATP-binding component | 1.11 | 0.953 |
| SG0964; GI:85058942; GeneID:3867091; galactoside ABC transporter ATP-binding | 1.11 | 0.897 |
| SG2076; GI:85060054; GeneID:3868107; type III secretion apparatus | 1.11 | 0.908 |
| SG1340; GI:85059318; GeneID:3866411; disulfide bond formation protein | 1.11 | 0.900 |
| SG2056; GI:85060034; GeneID:3866508; flagellar biosynthesis protein FliP | 1.11 | 0.879 |
| SG2125; GI:85060103; GeneID:3867286; putative sodium-hydrogen antiporter | 1.11 | 0.934 |
| SG1925; GI:85059903; GeneID:3867658; hypothetical protein | 1.11 | 0.597 |
| SG0770; GI:85058748; GeneID:3868037; suppressor for copper-sensitivity C | 1.11 | 0.910 |
| SG2112; GI:85060090; GeneID:3866253; hypothetical protein | 1.11 | 0.832 |
| SG0660; GI:85058638; GeneID:3868006; 4-methyl-5(beta-hydroxyethyl)-thiazole | 1.11 | 0.880 |
| SG0222; GI:85058200; GeneID:3868054; monofunctional biosynthetic peptidoglycan | 1.11 | 0.916 |
| SG1302; GI:85059280; GeneID:3867117; putative type III secretion apparatus | 1.11 | 0.890 |
| SG0704; GI:85058682; GeneID:3868621; peptidyl-prolyl cis-trans isomerase B | 1.11 | 0.903 |
| SG2387; GI:85060365; GeneID:3867358; thioredoxin 1 | 1.11 | 0.746 |
| SG1565; GI:85059543; GeneID:3866678; hypothetical protein | 1.11 | 0.919 |
| SG0318; GI:85058296; GeneID:3867390; hypothetical protein | 1.11 | 0.882 |
| SG1478; GI:85059456; GeneID:3866903; hypothetical protein | 1.11 | 0.890 |
| SG0513; GI:85058491; GeneID:3868214; enolase | 1.11 | 0.830 |
| SG0661; GI:85058639; GeneID:3868007; 2-dehydropantoate 2-reductase | 1.11 | 0.868 |
| SG2029; GI:85060007; GeneID:3866740; hypothetical protein | 1.11 | 0.924 |
| SG1791; GI:85059769; GeneID:3866794; sigma-E factor regulator | 1.11 | 0.898 |
| SG0482; GI:85058460; GeneID:3868696; hypoxanthine phosphoribosyltransferase | 1.11 | 0.904 |
| SGP1_0038; GI:85060449; GeneID:3866577; achromobactin ABC transporter periplasmic | 1.11 | 0.861 |
| SG1400; GI:85059378; GeneID:3867040; anthranilate synthase component II TrpD | 1.11 | 0.803 |
| SG1448; GI:85059426; GeneID:3867265; tyrosyl-tRNA synthase | 1.11 | 0.949 |
| SG2185; GI:85060163; GeneID:3866993; protein-export protein SecB | 1.11 | 0.932 |
| SG2389; GI:85060367; GeneID:3867360; guanosine pentaphosphatase exopolyphosphatase | 1.11 | 0.921 |
| SG1341; GI:85059319; GeneID:3866412; hypothetical protein | 1.11 | 0.847 |
| SG1266; GI:85059244; GeneID:3867873; hypothetical protein | 1.11 | 0.875 |
| SG2224; GI:85060202; GeneID:3868808; ATP-dependent DNA helicase | 1.11 | 0.828 |
| SG0221; GI:85058199; GeneID:3868053; sigma cross-reacting protein 27A (SCRP-27A) | 1.11 | 0.924 |
| SG1324; GI:85059302; GeneID:3867519; hypothetical protein | 1.11 | 0.910 |
| SG0464; GI:85058442; GeneID:3868204; quinolinate phosphoribosyltransferase | 1.11 | 0.911 |
| SG0540; GI:85058518; GeneID:3868656; putative phosphatase | 1.11 | 0.846 |
| SG0991; GI:85058969; GeneID:3866884; 3-phosphoshikimate 1-carboxyvinyltransferase | 1.11 | 0.936 |
| SG0483; GI:85058461; GeneID:3868697; carbonic anhydrase | 1.11 | 0.936 |
| SG1996; GI:85059974; GeneID:3867077; hypothetical protein | 1.11 | 0.674 |
| SG1210; GI:85059188; GeneID:3866849; putative phage reverse transcriptase/maturase | 1.11 | 0.916 |
| SG1953; GI:85059931; GeneID:3866211; putative glycosidase | 1.11 | 0.899 |
| SG0850; GI:85058828; GeneID:3867808; hypothetical protein | 1.11 | 0.960 |
| SG1427; GI:85059405; GeneID:3866988; phospho-2-dehydro-3-deoxyheptonate aldolase | 1.11 | 0.814 |
| SG1167; GI:85059145; GeneID:3866237; hypothetical protein | 1.11 | 0.921 |
| SG0501; GI:85058479; GeneID:3868280; hypothetical protein | 1.11 | 0.512 |
| SG1847; GI:85059825; GeneID:3868640; hypothetical protein | 1.11 | 0.891 |
| SG1223; GI:85059201; GeneID:3868676; hypothetical protein | 1.11 | 0.934 |
| SG0093; GI:85058071; GeneID:3867753; hypothetical protein | 1.11 | 0.960 |
| SG0740; GI:85058718; GeneID:3867717; hypothetical protein | 1.11 | 0.898 |
| SG1939; GI:85059917; GeneID:3867851; 1-deoxy-D-xylulose 5-phosphate reductoisomerase | 1.11 | 0.813 |
| SG2241; GI:85060219; GeneID:3866718; hypothetical protein | 1.11 | 0.837 |
| SG1024; GI:85059002; GeneID:3867210; putative lipoprotein protein | 1.11 | 0.906 |
| SG2065; GI:85060043; GeneID:3868096; response regulator for chemotaxis | 1.10 | 0.951 |
| SG1097; GI:85059075; GeneID:3868707; putative lipoprotein | 1.10 | 0.787 |
| SG0099; GI:85058077; GeneID:3867759; hypothetical protein | 1.10 | 0.768 |
| SG1249; GI:85059227; GeneID:3866772; hypothetical protein | 1.10 | 0.884 |
| SG1386; GI:85059364; GeneID:3867261; hypothetical protein | 1.10 | 0.822 |
| SG1909; GI:85059887; GeneID:3867642; thioredoxin 2 | 1.10 | 0.735 |
| SG0346; GI:85058324; GeneID:3868125; primosomal replication protein N | 1.10 | 0.729 |
| SG0974; GI:85058952; GeneID:3867101; 2'-deoxycytidine 5'-triphosphate deaminase | 1.10 | 0.920 |
| SG1068; GI:85059046; GeneID:3866708; hypothetical protein | 1.10 | 0.801 |
| SG1402; GI:85059380; GeneID:3867042; anthranilate synthase component I TrpE | 1.10 | 0.838 |
| SG1676; GI:85059654; GeneID:3868540; glutamyl-tRNA synthase | 1.10 | 0.879 |
| SG0762; GI:85058740; GeneID:3867739; hypothetical protein | 1.10 | 0.821 |
| SG2292; GI:85060270; GeneID:3866434; hypothetical protein | 1.10 | 0.450 |
| SG0844; GI:85058822; GeneID:3867802; hypothetical protein | 1.10 | 0.779 |
| SG0777; GI:85058755; GeneID:3868044; hypothetical protein | 1.10 | 0.775 |
| SG1713; GI:85059691; GeneID:3866852; hypothetical protein | 1.10 | 0.908 |
| SG1049; GI:85059027; GeneID:3866961; hypothetical protein | 1.10 | 0.745 |
| SG1646; GI:85059624; GeneID:3867824; hypothetical protein | 1.10 | 0.914 |
| SG1294; GI:85059272; GeneID:3867109; putative type III secretion apparatus | 1.10 | 0.868 |
| SG1911; GI:85059889; GeneID:3867644; CDP-diacylglycerol-serine | 1.10 | 0.729 |
| SG1950; GI:85059928; GeneID:3867344; hypothetical protein | 1.10 | 0.812 |
| SG1638; GI:85059616; GeneID:3867161; phage integrase | 1.10 | 0.845 |
| SG2411; GI:85060389; GeneID:3868410; ATP synthase delta subunit | 1.10 | 0.739 |
| SG2061; GI:85060039; GeneID:3866513; negative regulator of flagellin synthesis FlgM | 1.10 | 0.828 |
| SG1538; GI:85059516; GeneID:3867237; hemin ABC transporter periplasmic component | 1.10 | 0.861 |
| SG1768; GI:85059746; GeneID:3866927; peptidase B | 1.10 | 0.874 |
| SG1728; GI:85059706; GeneID:3866867; bacterioferritin comigratory protein | 1.10 | 0.656 |
| SG2013; GI:85059991; GeneID:3868792; hypothetical protein | 1.10 | 0.915 |
| SG0767; GI:85058745; GeneID:3868034; hypothetical protein | 1.10 | 0.847 |
| SG2176; GI:85060154; GeneID:3867950; triosephosphate isomerase | 1.10 | 0.859 |
| SG0887; GI:85058865; GeneID:3868335; peptidoglycan-associated lipoprotein | 1.10 | 0.524 |
| SG2286; GI:85060264; GeneID:3866428; 30S ribosomal protein S12 | 1.10 | 0.788 |
| SG1750; GI:85059728; GeneID:3866495; exodeoxyribonuclease VII large subunit | 1.10 | 0.847 |
| SG0898; GI:85058876; GeneID:3868346; putative molybdenum uptake operon | 1.10 | 0.905 |
| SG1428; GI:85059406; GeneID:3866931; hypothetical protein | 1.10 | 0.861 |
| SG1949; GI:85059927; GeneID:3867343; hypothetical protein | 1.10 | 0.671 |
| SG0843; GI:85058821; GeneID:3867801; hypothetical protein | 1.10 | 0.823 |
| SG1159; GI:85059137; GeneID:3866229; putative phage repressor | 1.10 | 0.826 |
| GI:125470066; GI:125470066; gp54; possible endopeptidase; similar to gp32 of pSOG3 | 1.10 | 0.906 |
| SG0766; GI:85058744; GeneID:3868033; hypothetical protein | 1.10 | 0.590 |
| SG1602; GI:85059580; GeneID:3867897; putative aminotransferase | 1.10 | 0.869 |
| SG1967; GI:85059945; GeneID:3866775; hypothetical protein | 1.10 | 0.817 |
| SG2113; GI:85060091; GeneID:3866254; ornithine carbamoyltransferase chain I | 1.10 | 0.740 |
| SG1740; GI:85059718; GeneID:3866485; transcriptional activator | 1.10 | 0.566 |
| SG0786; GI:85058764; GeneID:3868469; hypothetical protein | 1.10 | 0.827 |
| SG1154; GI:85059132; GeneID:3867145; thiamin biosynthesis lipoprotein | 1.10 | 0.836 |
| SG1160; GI:85059138; GeneID:3866230; hypothetical protein | 1.10 | 0.843 |
| SG2175; GI:85060153; GeneID:3867949; hypothetical protein | 1.10 | 0.881 |
| SG1071; GI:85059049; GeneID:3866711; hypothetical protein | 1.09 | 0.846 |
| SG0931; GI:85058909; GeneID:3868379; hypothetical protein | 1.09 | 0.933 |
| SG1356; GI:85059334; GeneID:3867328; hypothetical protein | 1.09 | 0.894 |
| SG1445; GI:85059423; GeneID:3866948; hypothetical protein | 1.09 | 0.802 |
| SG0706; GI:85058684; GeneID:3868726; 5,10-methylene-tetrahydrofolate dehydrogenase | 1.09 | 0.836 |
| SG2216; GI:85060194; GeneID:3868761; hypothetical protein | 1.09 | 0.877 |
| SG2031; GI:85060009; GeneID:3866742; hypothetical protein | 1.09 | 0.843 |
| SG2365; GI:85060343; GeneID:3866822; putative phage integrase | 1.09 | 0.738 |
| SG1977; GI:85059955; GeneID:3866785; thymidylate synthase | 1.09 | 0.812 |
| SG0746; GI:85058724; GeneID:3867723; hypothetical protein | 1.09 | 0.743 |
| SG0617; GI:85058595; GeneID:3867591; hypothetical protein | 1.09 | 0.940 |
| SG1015; GI:85058993; GeneID:3866379; asparaginyl-tRNA synthase | 1.09 | 0.787 |
| SG0421; GI:85058399; GeneID:3867532; dihydrofolate reductase | 1.09 | 0.821 |
| SG1523; GI:85059501; GeneID:3868249; hypothetical protein | 1.09 | 0.806 |
| SG0258; GI:85058236; GeneID:3867898; tRNA nucleotidyl transferase | 1.09 | 0.835 |
| SG0434; GI:85058412; GeneID:3867409; 3-isopropylmalate dehydratase small subunit | 1.09 | 0.655 |
| SG2018; GI:85059996; GeneID:3868797; biosynthetic arginine decarboxylase | 1.09 | 0.811 |
| SGP2_0004; GI:85060470; GeneID:3866605; hypothetical protein | 1.09 | 0.761 |
| SG1868; GI:85059846; GeneID:3867066; hypothetical protein | 1.09 | 0.681 |
| SG1744; GI:85059722; GeneID:3866489; putative hydrolase | 1.09 | 0.889 |
| SG1388; GI:85059366; GeneID:3867263; hypothetical protein | 1.09 | 0.778 |
| SG1456; GI:85059434; GeneID:3867273; hypothetical protein | 1.09 | 0.695 |
| SG1622; GI:85059600; GeneID:3867800; cell division protein | 1.09 | 0.877 |
| SG0738; GI:85058716; GeneID:3867715; hypothetical protein | 1.09 | 0.915 |
| SG0631; GI:85058609; GeneID:3868450; hypothetical protein | 1.09 | 0.562 |
| SG2350; GI:85060328; GeneID:3867178; hypothetical protein | 1.09 | 0.836 |
| SG0820; GI:85058798; GeneID:3868029; hypothetical protein | 1.09 | 0.874 |
| SG1321; GI:85059299; GeneID:3867516; hypothetical protein | 1.09 | 0.720 |
| SG0723; GI:85058701; GeneID:3868743; phage portal protein | 1.09 | 0.912 |
| SG1873; GI:85059851; GeneID:3866347; 2-dehydro-3-deoxyphosphooctonate aldolase | 1.09 | 0.800 |
| SG2343; GI:85060321; GeneID:3867171; putative phosphatase | 1.09 | 0.864 |
| SG2410; GI:85060388; GeneID:3868409; ATP synthase subunit B | 1.09 | 0.681 |
| SG0904; GI:85058882; GeneID:3868352; 8-amino-7-oxononanoate synthase | 1.09 | 0.746 |
| SG2010; GI:85059988; GeneID:3868789; ribose 5-phosphate isomerase A | 1.09 | 0.809 |
| SG1499; GI:85059477; GeneID:3868186; transcriptional regulator | 1.09 | 0.784 |
| SG0735; GI:85058713; GeneID:3867712; hypothetical protein | 1.09 | 0.757 |
| SG1486; GI:85059464; GeneID:3868173; putative short-chain dehydrogenase | 1.09 | 0.886 |
| SG0115; GI:85058093; GeneID:3867988; transcriptional activator | 1.09 | 0.761 |
| SG0795; GI:85058773; GeneID:3868478; penicillin-binding protein 5 | 1.09 | 0.824 |
| SG1736; GI:85059714; GeneID:3866481; polyphosphate kinase | 1.09 | 0.786 |
| SG0942; GI:85058920; GeneID:3868429; hypothetical protein | 1.09 | 0.682 |
| SG2149; GI:85060127; GeneID:3868258; putative spermidine/putrescine ABC transporter | 1.09 | 0.834 |
| SG1970; GI:85059948; GeneID:3866778; hypothetical protein | 1.09 | 0.797 |
| SG0639; GI:85058617; GeneID:3868458; hypothetical protein | 1.09 | 0.960 |
| SG1109; GI:85059087; GeneID:3868719; outer membrane lipoprotein carrier protein LolA | 1.09 | 0.755 |
| SG1771; GI:85059749; GeneID:3866930; putative RNA methyltransferase | 1.09 | 0.843 |
| SG0337; GI:85058315; GeneID:3868116; host factor-I protein (HF-I) | 1.09 | 0.737 |
| SG1715; GI:85059693; GeneID:3866854; transketolase 1 | 1.08 | 0.813 |
| SG0512; GI:85058490; GeneID:3868213; CTP synthase | 1.08 | 0.687 |
| SG0484; GI:85058462; GeneID:3868698; ABC transporter ATP-binding component | 1.08 | 0.609 |
| SG2054; GI:85060032; GeneID:3866506; flagellar motor switch protein FliN | 1.08 | 0.789 |
| SG1595; GI:85059573; GeneID:3867890; NADH dehydrogenase I subunit H | 1.08 | 0.695 |
| GI:125470052; GI:125470052; gp39; phage-related protein; similar to gp04 of pSG3 and | 1.08 | 0.820 |
| SG1316; GI:85059294; GeneID:3867511; carboxy-terminal proteinase | 1.08 | 0.686 |
| SG0021; GI:85057999; GeneID:3867678; flagellar transcriptional activator FlhD | 1.08 (1.2) | 0.712 |
| SG0536; GI:85058514; GeneID:3868652; amino-acid ABC transporter periplasmic | 1.08 | 0.689 |
| SG2291; GI:85060269; GeneID:3866433; FKBP-type peptidyl-prolyl cis-trans isomerase | 1.08 | 0.687 |
| SG1219; GI:85059197; GeneID:3868672; hypothetical protein | 1.08 | 0.769 |
| SG0126; GI:85058104; GeneID:3867960; pantothenate kinase | 1.08 | 0.761 |
| SG1008; GI:85058986; GeneID:3866372; putative phage replication protein | 1.08 | 0.795 |
| SG1797; GI:85059775; GeneID:3866800; uracil-DNA glycosylase | 1.08 | 0.738 |
| SG0632; GI:85058610; GeneID:3868451; hypothetical protein | 1.08 | 0.749 |
| SG2344; GI:85060322; GeneID:3867172; phage integrase | 1.08 | 0.779 |
| SG0390; GI:85058368; GeneID:3867637; DNA polymerase III psi subunit | 1.08 | 0.722 |
| SG0710; GI:85058688; GeneID:3868730; hypothetical protein | 1.08 | 0.759 |
| SG0171; GI:85058149; GeneID:3868498; hypothetical protein | 1.08 | 0.592 |
| SG2191; GI:85060169; GeneID:3866999; hypothetical protein | 1.08 | 0.726 |
| SG0680; GI:85058658; GeneID:3868147; putative ammonium transport protein | 1.08 | 0.758 |
| SG2037; GI:85060015; GeneID:3866748; membrane-bound lytic murein transglycosylase C | 1.08 | 0.767 |
| SG0047; GI:85058025; GeneID:3867432; flagellar protein FliL | 1.08 | 0.842 |
| SG1770; GI:85059748; GeneID:3866929; hypothetical protein | 1.08 | 0.596 |
| SG0013; GI:85057991; GeneID:3867670; hypothetical protein | 1.08 | 0.788 |
| SG1660; GI:85059638; GeneID:3867838; hypothetical protein | 1.08 | 0.768 |
| SG1806; GI:85059784; GeneID:3866809; hypothetical protein | 1.08 | 0.855 |
| SG0667; GI:85058645; GeneID:3868134; cytochrome o ubiquinol oxidase subunit II | 1.08 | 0.687 |
| SG0287; GI:85058265; GeneID:3868594; transposase | 1.08 | 0.603 |
| SG1891; GI:85059869; GeneID:3867764; hypothetical protein | 1.08 | 0.673 |
| SG0485; GI:85058463; GeneID:3868699; ABC transporter permease component | 1.08 | 0.683 |
| SG0858; GI:85058836; GeneID:3867816; glucosamine-6-phosphate deaminase | 1.08 | 0.664 |
| SG1215; GI:85059193; GeneID:3868668; phage terminase small subunit | 1.08 | 0.791 |
| SG1505; GI:85059483; GeneID:3868192; putative heme/hemoglobin transport protein | 1.08 | 0.734 |
| SG0291; GI:85058269; GeneID:3868598; hypothetical protein | 1.08 | 0.774 |
| SG1136; GI:85059114; GeneID:3867127; putative transcriptional regulator | 1.08 | 0.747 |
| SG0182; GI:85058160; GeneID:3866621; fucose transport protein | 1.08 | 0.902 |
| SG1903; GI:85059881; GeneID:3867776; putative transposase | 1.08 | 0.733 |
| SG1128; GI:85059106; GeneID:3867556; histidinol-phosphate aminotransferase HisC | 1.08 | 0.691 |
| SG0392; GI:85058370; GeneID:3867639; peptide chain release factor RF-3 | 1.08 | 0.729 |
| SG1881; GI:85059859; GeneID:3866355; peptidyl-tRNA hydrolase | 1.08 | 0.759 |
| SG1470; GI:85059448; GeneID:3866895; putative oxidoreductase | 1.08 | 0.517 |
| SG1093; GI:85059071; GeneID:3868170; arginine ABC transporter permease component | 1.08 | 0.740 |
| SG1546; GI:85059524; GeneID:3867186; hypothetical protein | 1.08 | 0.822 |
| SG1947; GI:85059925; GeneID:3867341; hypothetical protein | 1.08 | 0.501 |
| SG1233; GI:85059211; GeneID:3866756; hypothetical protein | 1.08 | 0.825 |
| SG1270; GI:85059248; GeneID:3867877; glucose-6-phosphate 1-dehydrogenase | 1.08 | 0.737 |
| SG0538; GI:85058516; GeneID:3868654; alanyl-tRNA synthase | 1.08 | 0.799 |
| SG2150; GI:85060128; GeneID:3868259; hypothetical protein | 1.08 | 0.691 |
| SG0024; GI:85058002; GeneID:3867448; chemotaxis protein MotB | 1.08 | 0.662 |
| SG0621; GI:85058599; GeneID:3867595; putative proline-sodium transport protein | 1.08 | 0.791 |
| SG2316; GI:85060294; GeneID:3866322; heat shock protein | 1.08 | 0.643 |
| SG0722; GI:85058700; GeneID:3868742; phage major head protein | 1.08 | 0.752 |
| SG0622; GI:85058600; GeneID:3867596; putative translation initiation inhibitor | 1.08 | 0.727 |
| SG0753; GI:85058731; GeneID:3867730; hypothetical protein | 1.08 | 0.811 |
| SG1444; GI:85059422; GeneID:3866947; outer membrane lipoprotein | 1.08 | 0.191 |
| SG0406; GI:85058384; GeneID:3867575; threonine synthase ThrC | 1.08 | 0.726 |
| SG1310; GI:85059288; GeneID:3867125; hypothetical protein | 1.08 | 0.644 |
| SG0846; GI:85058824; GeneID:3867804; phage tail core protein | 1.08 | 0.890 |
| SG0567; GI:85058545; GeneID:3867619; type III secretion apparatus SpaN | 1.07 | 0.642 |
| SG0209; GI:85058187; GeneID:3868080; putative anti-sigma-B factor antagonist | 1.07 | 0.745 |
| SG0027; GI:85058005; GeneID:3867451; flagellar protein FlgN | 1.07 | 0.702 |
| SG0231; GI:85058209; GeneID:3868063; hypothetical protein | 1.07 | 0.913 |
| SG1354; GI:85059332; GeneID:3867326; hypothetical protein | 1.07 | 0.712 |
| SG1598; GI:85059576; GeneID:3867893; NADH dehydrogenase I subunit E | 1.07 | 0.615 |
| SG0041; GI:85058019; GeneID:3867465; flagellar protein FliR | 1.07 | 0.881 |
| SG0528; GI:85058506; GeneID:3868229; stationary-phase survival protein | 1.07 | 0.711 |
| SG0888; GI:85058866; GeneID:3868336; hypothetical protein | 1.07 | 0.660 |
| SG0804; GI:85058782; GeneID:3868013; hypothetical protein | 1.07 | 0.581 |
| SG1918; GI:85059896; GeneID:3867651; putative lipoprotein | 1.07 | 0.716 |
| SG1986; GI:85059964; GeneID:3867067; putative transcriptional regulator | 1.07 | 0.500 |
| SG0524; GI:85058502; GeneID:3868225; hypothetical protein | 1.07 | 0.471 |
| SG1864; GI:85059842; GeneID:3867062; putative multidrug efflux transport protein | 1.07 | 0.720 |
| SG1627; GI:85059605; GeneID:3867150; chorismate synthase | 1.07 | 0.670 |
| SG0884; GI:85058862; GeneID:3868332; colicin import protein TolR | 1.07 | 0.702 |
| SG2069; GI:85060047; GeneID:3868100; arsenate reductase | 1.07 | 0.705 |
| SG1023; GI:85059001; GeneID:3867209; paraquat-inducible protein B | 1.07 | 0.674 |
| SG1807; GI:85059785; GeneID:3866810; hypothetical protein | 1.07 | 0.785 |
| SG0023; GI:85058001; GeneID:3867680; chemotaxis protein MotA | 1.07 | 0.698 |
| SG0082; GI:85058060; GeneID:3867742; hypothetical protein | 1.07 | 0.667 |
| SG1088; GI:85059066; GeneID:3868165; hypothetical protein | 1.07 | 0.805 |
| SG1337; GI:85059315; GeneID:3866408; hypothetical protein | 1.07 | 0.727 |
| GI:125470054; GI:125470054; gp41; possible phage-related recombination protein; | 1.07 | 0.726 |
| SG1590; GI:85059568; GeneID:3867885; NADH dehydrogenase I subunit M | 1.07 | 0.639 |
| SG0104; GI:85058082; GeneID:3867977; hypothetical protein | 1.07 | 0.801 |
| SG0029; GI:85058007; GeneID:3867453; flagellar basal body P-ring formation protein | 1.07 | 0.803 |
| SG1725; GI:85059703; GeneID:3866864; lipoprotein | 1.07 | 0.541 |
| SG1846; GI:85059824; GeneID:3868639; hypothetical protein | 1.07 | 0.700 |
| SG0900; GI:85058878; GeneID:3868348; hypothetical protein | 1.07 | 0.750 |
| SG0081; GI:85058059; GeneID:3867505; hypothetical protein | 1.07 | 0.679 |
| SG1361; GI:85059339; GeneID:3867333; DNA topoisomerase III | 1.07 | 0.716 |
| SG0373; GI:85058351; GeneID:3867485; phosphoglucomutase/phosphomannomutase MrsA | 1.07 | 0.700 |
| SG2193; GI:85060171; GeneID:3867001; ADP-L-glycero-D-manno-heptose-6-epimerase | 1.07 | 0.679 |
| SG1141; GI:85059119; GeneID:3867132; hypothetical protein | 1.07 | 0.706 |
| SG0405; GI:85058383; GeneID:3867574; homoserine kinase ThrB | 1.07 | 0.698 |
| SG2209; GI:85060187; GeneID:3868754; DNA/pantothenate metabolism flavoprotein | 1.07 | 0.677 |
| SG2014; GI:85059992; GeneID:3868793; fructose-bisphosphate aldolase class II | 1.07 | 0.446 |
| SG1139; GI:85059117; GeneID:3867130; putative transcriptional regulator | 1.07 | 0.576 |
| SG1623; GI:85059601; GeneID:3867146; 3-oxoacyl-(acyl-carrier-protein) synthase I | 1.07 | 0.482 |
| SG1563; GI:85059541; GeneID:3867203; putative phage capsid scaffolding protein | 1.07 | 0.724 |
| SG0164; GI:85058142; GeneID:3868491; putative succinate-semialdehyde dehydrogenase | 1.07 | 0.747 |
| SG1204; GI:85059182; GeneID:3866843; putative phage tail protein | 1.07 | 0.763 |
| SG1099; GI:85059077; GeneID:3868709; hypothetical protein | 1.07 | 0.675 |
| SG1030; GI:85059008; GeneID:3867216; outer membrane protein A precursor | 1.07 | 0.463 |
| SG0207; GI:85058185; GeneID:3868078; hypothetical protein | 1.07 | 0.723 |
| SG1659; GI:85059637; GeneID:3867837; hypothetical protein | 1.07 | 0.635 |
| SG1319; GI:85059297; GeneID:3867514; hypothetical protein | 1.07 | 0.419 |
| SG1653; GI:85059631; GeneID:3867831; hypothetical protein | 1.07 | 0.630 |
| SG1217; GI:85059195; GeneID:3868670; hypothetical protein | 1.07 | 0.629 |
| SG0503; GI:85058481; GeneID:3868282; 5'-methylthioadenosine/S-adenosylhomocysteine | 1.07 | 0.586 |
| SG1834; GI:85059812; GeneID:3868627; hypothetical protein | 1.07 | 0.609 |
| SG0721; GI:85058699; GeneID:3868741; hypothetical protein | 1.07 | 0.799 |
| SG1344; GI:85059322; GeneID:3866415; hypothetical protein | 1.07 | 0.589 |
| SG2081; GI:85060059; GeneID:3868112; type III secretion apparatus | 1.07 | 0.752 |
| SG1218; GI:85059196; GeneID:3868671; hypothetical protein | 1.06 | 0.632 |
| SG1732; GI:85059710; GeneID:3866871; phosphoribosylaminoimidazole synthase | 1.06 | 0.553 |
| SG0188; GI:85058166; GeneID:3866627; hypothetical protein | 1.06 | 0.581 |
| SG0975; GI:85058953; GeneID:3867102; outer membrane assembly protein | 1.06 | 0.643 |
| SG1829; GI:85059807; GeneID:3866656; hypothetical protein | 1.06 | 0.459 |
| SG0780; GI:85058758; GeneID:3868047; hypothetical protein | 1.06 | 0.695 |
| SG1296; GI:85059274; GeneID:3867111; putative type III secretion apparatus | 1.06 | 0.600 |
| SG1697; GI:85059675; GeneID:3866280; hypothetical protein | 1.06 | 0.579 |
| SG0953; GI:85058931; GeneID:3868440; hypothetical protein | 1.06 | 0.687 |
| SG2051; GI:85060029; GeneID:3866503; hypothetical protein | 1.06 | 0.801 |
| SG2196; GI:85060174; GeneID:3867004; hypothetical protein | 1.06 | 0.473 |
| SG1561; GI:85059539; GeneID:3867201; hypothetical protein | 1.06 | 0.620 |
| SG0317; GI:85058295; GeneID:3867389; hypothetical protein | 1.06 | 0.609 |
| SG1293; GI:85059271; GeneID:3867108; putative type III secretion apparatus | 1.06 | 0.618 |
| SG1991; GI:85059969; GeneID:3867072; single-stranded-DNA-specific exonuclease | 1.06 | 0.605 |
| SG0389; GI:85058367; GeneID:3867636; ribosomal RNA small subunit methyltransferase C | 1.06 | 0.600 |
| SG0814; GI:85058792; GeneID:3868023; N-acetylglucosamine repressor | 1.06 | 0.536 |
| SG0038; GI:85058016; GeneID:3867462; flagellar protein FlgJ | 1.06 | 0.615 |
| SG1298; GI:85059276; GeneID:3867113; type III secretion apparatus | 1.06 | 0.583 |
| SG1783; GI:85059761; GeneID:3866527; holo-(acyl-carrier protein) synthase | 1.06 | 0.624 |
| SG0376; GI:85058354; GeneID:3867623; transcription pausing L factor | 1.06 | 0.503 |
| SG0757; GI:85058735; GeneID:3867734; hypothetical protein | 1.06 | 0.596 |
| SG1405; GI:85059383; GeneID:3867045; putative pseudouridine synthase | 1.06 | 0.515 |
| SG1125; GI:85059103; GeneID:3867553; 1-(5-phosphoribosyl)-5-[(5-phosphoribosylamino) | 1.06 | 0.616 |
| SG1604; GI:85059582; GeneID:3867782; putative phosphatase | 1.06 | 0.607 |
| SG2357; GI:85060335; GeneID:3866814; phage capsid protein | 1.06 | 0.610 |
| SG1852; GI:85059830; GeneID:3867050; putative transcriptional regulator | 1.06 | 0.372 |
| SG1501; GI:85059479; GeneID:3868188; phage shock protein C | 1.06 | 0.576 |
| SG0223; GI:85058201; GeneID:3868055; hypothetical protein | 1.06 | 0.638 |
| SG1926; GI:85059904; GeneID:3867659; cell cycle protein | 1.06 | 0.557 |
| SG0715; GI:85058693; GeneID:3868735; hypothetical protein | 1.06 | 0.637 |
| SG1515; GI:85059493; GeneID:3868241; hypothetical protein | 1.06 | 0.577 |
| SG1536; GI:85059514; GeneID:3867235; putative two-component regulator protein | 1.06 | 0.494 |
| SG0404; GI:85058382; GeneID:3867573; aspartokinase I ThrA | 1.06 | 0.603 |
| SG1700; GI:85059678; GeneID:3866446; cysteine synthase A | 1.06 | 0.597 |
| SG0592; GI:85058570; GeneID:3866660; DNA polymerase III epsilon subunit | 1.06 | 0.587 |
| SG1431; GI:85059409; GeneID:3866934; hypothetical protein | 1.06 | 0.538 |
| SG2373; GI:85060351; GeneID:3866830; putative sugar-nucleotide epimerase/dehydratase | 1.06 | 0.783 |
| SG1278; GI:85059256; GeneID:3866388; hypothetical protein | 1.06 | 0.434 |
| SG1813; GI:85059791; GeneID:3866640; hypothetical protein | 1.06 | 0.576 |
| SGP1_0054; GI:85060465; GeneID:3866539; hypothetical protein | 1.06 | 0.837 |
| SG1737; GI:85059715; GeneID:3866482; exopolyphosphatase | 1.06 | 0.531 |
| SG0331; GI:85058309; GeneID:3867403; putative GDP-mannose dehydratase | 1.06 | 0.592 |
| SG0328; GI:85058306; GeneID:3867400; KDP operon transcriptional regulatory protein | 1.06 | 0.515 |
| SG0467; GI:85058445; GeneID:3868207; pyruvate dehydrogenase E1 component | 1.06 | 0.357 |
| SG2117; GI:85060095; GeneID:3866258; hypothetical protein | 1.06 | 0.523 |
| SG1582; GI:85059560; GeneID:3866695; DNA gyrase subunit A | 1.06 | 0.496 |
| SG1854; GI:85059832; GeneID:3867052; putative phosphonate metabolism protein PhnP | 1.06 | 0.520 |
| SG2227; GI:85060205; GeneID:3868811; hypothetical protein | 1.06 | 0.471 |
| SG1017; GI:85058995; GeneID:3866381; aminopeptidase N | 1.06 | 0.563 |
| SG0228; GI:85058206; GeneID:3868060; putative lysR-family transcriptional regulator | 1.06 | 0.594 |
| SG1011; GI:85058989; GeneID:3866375; putative phage tail protein | 1.06 | 0.715 |
| SG1789; GI:85059767; GeneID:3866533; GTP-binding elongation factor LepA | 1.06 | 0.572 |
| SG1567; GI:85059545; GeneID:3866680; putative phage tail protein | 1.06 | 0.578 |
| SG2093; GI:85060071; GeneID:3866468; putative efflux transport protein | 1.06 | 0.562 |
| SG1397; GI:85059375; GeneID:3867037; tryptophan synthase alpha subunit TrpA | 1.06 | 0.566 |
| SG0687; GI:85058665; GeneID:3868604; primosomal replication protein N | 1.06 | 0.480 |
| SG1495; GI:85059473; GeneID:3868182; hypothetical protein | 1.06 | 0.561 |
| SG1482; GI:85059460; GeneID:3866907; hypothetical protein | 1.06 | 0.729 |
| SG0192; GI:85058170; GeneID:3866631; hypothetical protein | 1.06 | 0.447 |
| SG2232; GI:85060210; GeneID:3868816; two-component nitrogen regulation protein | 1.06 | 0.565 |
| SG1650; GI:85059628; GeneID:3867828; hypothetical protein | 1.06 | 0.590 |
| SG2090; GI:85060068; GeneID:3866465; type III secretion apparatus InvA | 1.06 | 0.691 |
| SG0646; GI:85058624; GeneID:3868465; queuine tRNA-ribosyltransferase | 1.06 | 0.574 |
| SG0330; GI:85058308; GeneID:3867402; GDP-L-fucose-synthase | 1.06 | 0.704 |
| SG2370; GI:85060348; GeneID:3866827; hypothetical protein | 1.06 | 0.647 |
| SG1509; GI:85059487; GeneID:3868235; hypothetical protein | 1.06 | 0.703 |
| SG2075; GI:85060053; GeneID:3868106; putative type III secretion apparatus | 1.06 | 0.491 |
| SG0507; GI:85058485; GeneID:3868286; putative tartronate semialdehyde reductase | 1.06 | 0.536 |
| SG0626; GI:85058604; GeneID:3867600; putative 2-hydroxyacid-family dehydrogenase | 1.06 | 0.528 |
| SG0127; GI:85058105; GeneID:3867961; elongation factor Tu | 1.06 | 0.397 |
| SG1871; GI:85059849; GeneID:3866345; cation transport regulator ChaC | 1.06 | 0.421 |
| SG0498; GI:85058476; GeneID:3868277; putative ABC transport system permease | 1.05 | 0.555 |
| SG0445; GI:85058423; GeneID:3867420; UDP-N-acetylmuramoylalanyl-D-glutamyl-2,6- | 1.05 | 0.458 |
| SG2320; GI:85060298; GeneID:3867305; transcription elongation factor GreB | 1.05 | 0.527 |
| SG1031; GI:85059009; GeneID:3867217; DNA helicase IV | 1.05 | 0.474 |
| SG0077; GI:85058055; GeneID:3867501; hypothetical protein | 1.05 | 0.533 |
| SG1898; GI:85059876; GeneID:3867771; hypothetical protein | 1.05 | 0.426 |
| SG0347; GI:85058325; GeneID:3868126; 30S ribosomal protein S18 | 1.05 | 0.490 |
| SG0009; GI:85057987; GeneID:3867666; valine-pyruvate aminotransferase | 1.05 | 0.543 |
| SG2314; GI:85060292; GeneID:3866320; hypothetical protein | 1.05 | 0.489 |
| SG1665; GI:85059643; GeneID:3868529; hypothetical protein | 1.05 | 0.681 |
| SG2180; GI:85060158; GeneID:3867954; two-component response regulator | 1.05 | 0.449 |
| SG0371; GI:85058349; GeneID:3867483; cell division protein | 1.05 | 0.444 |
| SG1763; GI:85059741; GeneID:3866922; hypothetical protein | 1.05 | 0.496 |
| SG1934; GI:85059912; GeneID:3867846; outer membrane protein | 1.05 | 0.372 |
| SG1206; GI:85059184; GeneID:3866845; hypothetical protein | 1.05 | 0.563 |
| SG1330; GI:85059308; GeneID:3866401; hypothetical protein | 1.05 | 0.425 |
| SG1392; GI:85059370; GeneID:3867032; hypothetical protein | 1.05 | 0.579 |
| SG2038; GI:85060016; GeneID:3866749; hypothetical protein | 1.05 | 0.503 |
| SG0201; GI:85058179; GeneID:3868072; hypothetical protein | 1.05 | 0.545 |
| SG2074; GI:85060052; GeneID:3868105; putative type III secretion apparatus | 1.05 | 0.490 |
| SG0452; GI:85058430; GeneID:3867427; cell division protein FtsA | 1.05 | 0.505 |
| GI:125470025; GI:125470025; gp09; possible virion protein; similar to gp14 of P22 | 1.05 | 0.767 |
| SG2284; GI:85060262; GeneID:3866426; elongation factor G | 1.05 (1.23) | 0.377 |
| SG0190; GI:85058168; GeneID:3866629; hypothetical protein | 1.05 | 0.695 |
| SG1946; GI:85059924; GeneID:3867858; 2,3,4,5-tetrahydropyridine-2-carboxylate | 1.05 | 0.480 |
| SG1237; GI:85059215; GeneID:3866760; hypothetical protein | 1.05 | 0.437 |
| SG0342; GI:85058320; GeneID:3868121; adenylosuccinate synthase | 1.05 | 0.409 |
| SG0143; GI:85058121; GeneID:3868509; phosphoribosylaminoimidazolecarboxamide | 1.05 | 0.566 |
| SG1534; GI:85059512; GeneID:3867233; putative carbohydrate kinase | 1.05 | 0.577 |
| SG1284; GI:85059262; GeneID:3866394; putative secreted effector protein | 1.05 | 0.558 |
| SG0829; GI:85058807; GeneID:3868771; hypothetical protein | 1.05 | 0.637 |
| SG0268; GI:85058246; GeneID:3867908; hypothetical protein | 1.05 | 0.492 |
| SG1816; GI:85059794; GeneID:3866643; hypothetical protein | 1.05 | 0.455 |
| SG0018; GI:85057996; GeneID:3867675; hypothetical protein | 1.05 | 0.496 |
| SG0725; GI:85058703; GeneID:3868745; hypothetical protein | 1.05 | 0.699 |
| SG0204; GI:85058182; GeneID:3868075; putative isomerase | 1.05 | 0.566 |
| SG2324; GI:85060302; GeneID:3867309; putative biotin biosynthesis protein | 1.05 | 0.442 |
| SG1776; GI:85059754; GeneID:3866520; nitrogen regulatory protein P-II | 1.05 | 0.374 |
| SG0414; GI:85058392; GeneID:3867525; isoleucyl-tRNA synthase | 1.05 | 0.473 |
| SG0339; GI:85058317; GeneID:3868118; hypothetical protein | 1.05 | 0.475 |
| SG0044; GI:85058022; GeneID:3867429; flagellar protein FliO | 1.05 | 0.536 |
| SG0156; GI:85058134; GeneID:3868522; rod shape-determining protein MreB | 1.05 | 0.351 |
| SG1156; GI:85059134; GeneID:3866226; putative phage integrase | 1.05 | 0.695 |
| SG1362; GI:85059340; GeneID:3867334; putative pyrophosphohydrolase | 1.05 | 0.450 |
| SG1856; GI:85059834; GeneID:3867054; hypothetical protein | 1.05 | 0.625 |
| SG0960; GI:85058938; GeneID:3867087; two-component system sensor kinase | 1.05 | 0.455 |
| SG1708; GI:85059686; GeneID:3866454; sulfate ABC transporter permease component CysT | 1.05 | 0.280 |
| SG0837; GI:85058815; GeneID:3868779; hypothetical protein | 1.05 | 0.668 |
| SGP3_0006; GI:85060496; GeneID:3866617; putative replication protein | 1.05 | 0.371 |
| SG1887; GI:85059865; GeneID:3866361; putative sugar-phosphate isomerase | 1.05 | 0.347 |
| SG0234; GI:85058212; GeneID:3868066; hypothetical protein | 1.05 | 0.502 |
| SG1558; GI:85059536; GeneID:3867198; hypothetical protein | 1.05 | 0.475 |
| SG0866; GI:85058844; GeneID:3868314; phosphoglucomutase Pgm | 1.05 | 0.532 |
| GI:125470046; GI:125470046; gp31; possible lysozyme; similar to gp14 of pSG3 and gp53 | 1.05 | 0.403 |
| SG0999; GI:85058977; GeneID:3866363; hypothetical protein | 1.05 | 0.419 |
| SG0839; GI:85058817; GeneID:3868781; hypothetical protein | 1.05 | 0.599 |
| SG1306; GI:85059284; GeneID:3867121; putative type III secretion apparatus | 1.05 | 0.477 |
| GI:125470042; GI:125470042; gp27; hypothetical protein; similar to gp18 of pSG3 | 1.05 | 0.430 |
| SG0062; GI:85058040; GeneID:3867447; dipeptide ABC transporter permease component | 1.05 | 0.426 |
| SG2392; GI:85060370; GeneID:3868091; lysR-family transcriptional regulator | 1.05 | 0.400 |
| SG0350; GI:85058328; GeneID:3868129; inositol monophosphatase family protein | 1.05 | 0.443 |
| SG2028; GI:85060006; GeneID:3866739; pyrroline-5-carboxylate reductase | 1.05 | 0.530 |
| SG1544; GI:85059522; GeneID:3867243; putative iron ABC transporter ATP-binding | 1.05 | 0.443 |
| SG2047; GI:85060025; GeneID:3866499; putative transcriptional regulator | 1.05 | 0.350 |
| SG0776; GI:85058754; GeneID:3868043; hypothetical protein | 1.05 | 0.443 |
| SG0756; GI:85058734; GeneID:3867733; hypothetical protein | 1.05 | 0.519 |
| SG1566; GI:85059544; GeneID:3866679; putative phage DNA modification | 1.05 | 0.543 |
| SG2115; GI:85060093; GeneID:3866256; aspartate carbamoyltransferase catalytic | 1.05 | 0.401 |
| SG1464; GI:85059442; GeneID:3867281; putative transport protein | 1.05 | 0.478 |
| SG0468; GI:85058446; GeneID:3868208; pyruvate dehydrogenase | 1.05 | 0.346 |
| SG1258; GI:85059236; GeneID:3867865; dATP pyrophosphohydrolase | 1.05 | 0.450 |
| SG0358; GI:85058336; GeneID:3867470; hypothetical protein | 1.05 | 0.391 |
| SG0825; GI:85058803; GeneID:3868767; hypothetical protein | 1.05 | 0.586 |
| SG0197; GI:85058175; GeneID:3866636; nitrogen regulatory IIA protein | 1.05 | 0.546 |
| SG1923; GI:85059901; GeneID:3867656; hypothetical protein | 1.05 | 0.493 |
| SG0921; GI:85058899; GeneID:3868369; D-alanyl-D-alanine carboxypeptidase | 1.05 | 0.422 |
| SG2267; GI:85060245; GeneID:3866292; 50S ribosomal protein L24 | 1.05 | 0.299 |
| SG1944; GI:85059922; GeneID:3867856; methionine aminopeptidase | 1.05 | 0.443 |
| SG0487; GI:85058465; GeneID:3868701; pantothenate synthase | 1.05 | 0.453 |
| SG0572; GI:85058550; GeneID:3867686; type III secretion apparatus SpaS | 1.05 | 0.688 |
| SG0711; GI:85058689; GeneID:3868731; putative phage tail protein | 1.05 | 0.542 |
| SG0372; GI:85058350; GeneID:3867484; dihydropteroate synthase | 1.05 | 0.477 |
| SG0050; GI:85058028; GeneID:3867435; flagellum-specific ATP synthase FliI | 1.05 | 0.498 |
| SG2375; GI:85060353; GeneID:3866832; putative 4-alpha-L-fucosyltransferase | 1.05 | 0.460 |
| SG1762; GI:85059740; GeneID:3866921; putative fimbrial biogenesis protein | 1.05 | 0.420 |
| SG1102; GI:85059080; GeneID:3868712; ATP-dependent Clp protease ATP-binding | 1.05 | 0.355 |
| SG2403; GI:85060381; GeneID:3868402; transcriptional regulator AsnC | 1.05 | 0.424 |
| SG0438; GI:85058416; GeneID:3867413; fructose operon repressor | 1.05 | 0.136 |
| SG0672; GI:85058650; GeneID:3868139; ATP-dependent Clp protease ATP-binding subunit | 1.05 | 0.306 |
| SG1812; GI:85059790; GeneID:3866639; hypothetical protein | 1.04 | 0.435 |
| SG1352; GI:85059330; GeneID:3867324; hypothetical protein | 1.04 | 0.381 |
| SG1077; GI:85059055; GeneID:3868154; lipoprotein releasing system ATP-binding protein | 1.04 | 0.388 |
| SG0971; GI:85058949; GeneID:3867098; hypothetical protein | 1.04 | 0.415 |
| SG2356; GI:85060334; GeneID:3867184; hypothetical protein | 1.04 | 0.603 |
| SG1075; GI:85059053; GeneID:3866715; transcription-repair coupling factor | 1.04 | 0.394 |
| SG2025; GI:85060003; GeneID:3866736; putative rhamnulose-1-phosphate aldolase | 1.04 | 0.442 |
| SG1699; GI:85059677; GeneID:3866282; phage integrase | 1.04 | 0.615 |
| SG0430; GI:85058408; GeneID:3867541; RNA polymerase associated helicase | 1.04 | 0.407 |
| SG0048; GI:85058026; GeneID:3867433; flagellar hook-length control protein FliK | 1.04 | 0.474 |
| SG0705; GI:85058683; GeneID:3868622; cysteinyl-tRNA synthase | 1.04 | 0.475 |
| SG0712; GI:85058690; GeneID:3868732; hypothetical protein | 1.04 | 0.368 |
| SG2355; GI:85060333; GeneID:3867183; hypothetical protein | 1.04 | 0.555 |
| SG2301; GI:85060279; GeneID:3866307; cAMP-regulatory protein | 1.04 | 0.232 |
| SG1599; GI:85059577; GeneID:3867894; NADH dehydrogenase I subunit C/D | 1.04 | 0.277 |
| SG0226; GI:85058203; GeneID:3868058; putative glycosylase | 1.04 | 0.371 |
| SG0241; GI:85058219; GeneID:3868293; putative transcriptional regulator | 1.04 | 0.386 |
| SG1504; GI:85059482; GeneID:3868191; hypothetical protein | 1.04 | 0.580 |
| SG0039; GI:85058017; GeneID:3867463; flagellar hook-associated protein 1 FlgK | 1.04 | 0.484 |
| SG0179; GI:85058157; GeneID:3868506; fucose operon protein | 1.04 | 0.370 |
| SG1603; GI:85059581; GeneID:3867781; hypothetical protein | 1.04 | 0.369 |
| SG2417; GI:85060395; GeneID:3868416; glucosamine-fructose-6-phosphate | 1.04 | 0.410 |
| SG2266; GI:85060244; GeneID:3866291; 50S ribosomal protein L5 | 1.04 | 0.254 |
| SG1880; GI:85059858; GeneID:3866354; ribose-phosphate pyrophosphokinase | 1.04 | 0.427 |
| SG1295; GI:85059273; GeneID:3867110; type III secretion apparatus | 1.04 | 0.362 |
| SG1640; GI:85059618; GeneID:3867163; putative phage outer membrane protein | 1.04 | 0.565 |
| SG1087; GI:85059065; GeneID:3868164; putative phage integrase | 1.04 | 0.344 |
| SG0319; GI:85058297; GeneID:3867391; hypothetical protein | 1.04 | 0.402 |
| SG2331; GI:85060309; GeneID:3867316; hypothetical protein | 1.04 | 0.486 |
| SG0578; GI:85058556; GeneID:3867692; 3-deoxy-D-arabino-heptulosonate-7-phosphate | 1.04 | 0.332 |
| SG0598; GI:85058576; GeneID:3866666; xanthine-guanine phosphoribosyltransferase | 1.04 | 0.409 |
| SG1597; GI:85059575; GeneID:3867892; NADH dehydrogenase I subunit F | 1.04 | 0.364 |
| SG0703; GI:85058681; GeneID:3868620; putative UDP-2,3-diacylglucosamine hydrolase | 1.04 | 0.362 |
| SG0793; GI:85058771; GeneID:3868476; lipoate-protein ligase B | 1.04 | 0.324 |
| SG1151; GI:85059129; GeneID:3867142; putative transport protein | 1.04 | 0.318 |
| SG1618; GI:85059596; GeneID:3867796; hypothetical protein | 1.04 | 0.396 |
| SG1948; GI:85059926; GeneID:3867342; hypothetical protein | 1.04 | 0.318 |
| SG1738; GI:85059716; GeneID:3866483; putative magnesium transport protein | 1.04 | 0.352 |
| SG0842; GI:85058820; GeneID:3868784; hypothetical protein | 1.04 | 0.470 |
| SGP1_0037; GI:85060448; GeneID:3866576; achromobactin ABC transporter permease component | 1.04 | 0.377 |
| SG0784; GI:85058762; GeneID:3868467; putative lipid A biosynthesis acyltransferase | 1.04 | 0.303 |
| SG2283; GI:85060261; GeneID:3866425; elongation factor Tu | 1.04 | 0.272 |
| SG2249; GI:85060227; GeneID:3866726; potassium transport protein | 1.04 | 0.388 |
| SG1548; GI:85059526; GeneID:3867188; putative fumarate reductase subunit D | 1.04 | 0.378 |
| SG0893; GI:85058871; GeneID:3868341; 3-deoxy-D-arabino-heptulosonate-7-phosphate | 1.04 | 0.401 |
| SG1407; GI:85059385; GeneID:3867047; hypothetical protein | 1.04 | 0.258 |
| SG1914; GI:85059892; GeneID:3867647; hypothetical protein | 1.04 | 0.390 |
| SG0636; GI:85058614; GeneID:3868455; hypothetical protein | 1.04 | 0.301 |
| SG1951; GI:85059929; GeneID:3867345; hypothetical protein | 1.04 | 0.432 |
| SG0638; GI:85058616; GeneID:3868457; hypothetical protein | 1.04 | 0.403 |
| SG1726; GI:85059704; GeneID:3866865; dihydrodipicolinate synthase | 1.04 | 0.244 |
| SG0674; GI:85058652; GeneID:3868141; DNA-binding protein HU-beta | 1.04 | 0.128 |
| SG1010; GI:85058988; GeneID:3866374; hypothetical protein | 1.04 | 0.383 |
| SG0302; GI:85058280; GeneID:3868570; putative cation tolerance protein | 1.04 | 0.343 |
| SG1531; GI:85059509; GeneID:3867230; hypothetical protein | 1.04 | 0.418 |
| SG1858; GI:85059836; GeneID:3867056; propanediol diffusion facilitator | 1.04 | 0.504 |
| SG1211; GI:85059189; GeneID:3866850; hypothetical protein | 1.04 | 0.462 |
| SG2041; GI:85060019; GeneID:3866752; putative PTS system IIA component | 1.04 | 0.152 |
| SG1902; GI:85059880; GeneID:3867775; putative glutamine synthase III | 1.04 | 0.359 |
| SG0286; GI:85058264; GeneID:3868593; hypothetical protein | 1.04 | 0.307 |
| SG0896; GI:85058874; GeneID:3868344; putative galactose-1-phosphate | 1.04 | 0.228 |
| SG1048; GI:85059026; GeneID:3866960; hypothetical protein | 1.04 | 0.383 |
| SG1104; GI:85059082; GeneID:3868714; cytochrome-related ABC transporter ATP-binding | 1.04 | 0.311 |
| SG1039; GI:85059017; GeneID:3866951; putative phage lysozyme lysis protein | 1.04 | 0.337 |
| SG1990; GI:85059968; GeneID:3867071; peptide chain release factor RF-2 | 1.04 | 0.219 |
| SG1814; GI:85059792; GeneID:3866641; hypothetical protein | 1.04 | 0.341 |
| SG1683; GI:85059661; GeneID:3866266; phage tail fiber assembly protein | 1.04 | 0.316 |
| SG0361; GI:85058339; GeneID:3867473; hypothetical protein | 1.04 | 0.374 |
| SG0708; GI:85058686; GeneID:3868728; hypothetical protein | 1.04 | 0.471 |
| SG0289; GI:85058267; GeneID:3868596; putative branched-chain amino acid ABC | 1.04 | 0.492 |
| SGP2_0017; GI:85060483; GeneID:3866595; hypothetical protein | 1.04 | 0.363 |
| SG2312; GI:85060290; GeneID:3866318; hypothetical protein | 1.04 | 0.327 |
| SG1308; GI:85059286; GeneID:3867123; putative type III secretion apparatus | 1.04 | 0.338 |
| SG1216; GI:85059194; GeneID:3868669; hypothetical protein | 1.04 | 0.363 |
| GI:125470022; GI:125470022; gp06; possible DNA stabilization protein; similar to HkbU | 1.04 | 0.484 |
| SG1795; GI:85059773; GeneID:3866798; ATP-dependent RNA helicase | 1.04 | 0.321 |
| SG2138; GI:85060116; GeneID:3867299; hypothetical protein | 1.04 | 0.356 |
| SG0370; GI:85058348; GeneID:3867482; cell division protein | 1.04 | 0.317 |
| SG1571; GI:85059549; GeneID:3866684; putative ATP-dependent helicase | 1.04 | 0.285 |
| SG2399; GI:85060377; GeneID:3868391; hypothetical protein | 1.04 | 0.319 |
| SG1391; GI:85059369; GeneID:3867031; hypothetical protein | 1.04 | 0.395 |
| SG1332; GI:85059310; GeneID:3866403; hypothetical protein | 1.04 | 0.272 |
| SG0299; GI:85058277; GeneID:3868567; bactoprenol glucosyl transferase | 1.04 | 0.494 |
| SG0930; GI:85058908; GeneID:3868378; hypothetical protein | 1.04 | 0.346 |
| SG1037; GI:85059015; GeneID:3867223; putative sulfite reductase | 1.04 | 0.328 |
| SG1869; GI:85059847; GeneID:3866343; putative invasin | 1.04 | 0.326 |
| SG0457; GI:85058435; GeneID:3868197; preprotein translocase SecA | 1.04 | 0.377 |
| SG0323; GI:85058301; GeneID:3867395; hypothetical protein | 1.04 | 0.291 |
| SG0752; GI:85058730; GeneID:3867729; hypothetical protein | 1.04 | 0.355 |
| SG1931; GI:85059909; GeneID:3867843; acyl-(acyl-carrier-protein)--UDP-N- | 1.04 | 0.273 |
| SG2254; GI:85060232; GeneID:3866731; 30S ribosomal protein S4 | 1.04 | 0.236 |
| SG1583; GI:85059561; GeneID:3866696; 3-demethylubiquinone-9 3-methyltransferase | 1.03 | 0.265 |
| SG0184; GI:85058162; GeneID:3866623; putative sugar ABC transporter periplasmic | 1.03 | 0.411 |
| SG1044; GI:85059022; GeneID:3866956; hypothetical protein | 1.03 | 0.336 |
| SG0670; GI:85058648; GeneID:3868137; trigger factor | 1.03 | 0.309 |
| SG0307; GI:85058285; GeneID:3868575; hypothetical protein | 1.03 | 0.294 |
| SG0281; GI:85058259; GeneID:3868588; biopolymer transport protein | 1.03 | 0.322 |
| SG2205; GI:85060183; GeneID:3868750; phosphopantetheine adenylyltransferase | 1.03 | 0.361 |
| SG1574; GI:85059552; GeneID:3866687; hypothetical protein | 1.03 | 0.265 |
| SG0761; GI:85058739; GeneID:3867738; hypothetical protein | 1.03 | 0.354 |
| SG2114; GI:85060092; GeneID:3866255; hypothetical protein | 1.03 | 0.366 |
| SG1083; GI:85059061; GeneID:3868160; adenylosuccinate lyase | 1.03 | 0.329 |
| SG0172; GI:85058150; GeneID:3868499; hypothetical protein | 1.03 | 0.244 |
| SG1723; GI:85059701; GeneID:3866862; hypothetical protein | 1.03 | 0.294 |
| SG1907; GI:85059885; GeneID:3867780; putative transcriptional regulator | 1.03 | 0.248 |
| SG2427; GI:85060405; GeneID:3868426; putative PTS system IIA component | 1.03 | 0.388 |
| SGP1_0004; GI:85060415; GeneID:3866543; hypothetical protein | 1.03 | 0.345 |
| SG1283; GI:85059261; GeneID:3866393; putative type III secretion apparatus | 1.03 | 0.339 |
| SG2281; GI:85060259; GeneID:3866423; hypothetical protein | 1.03 | 0.319 |
| SG2092; GI:85060070; GeneID:3866467; putative type III secretion apparatus | 1.03 | 0.285 |
| SG1878; GI:85059856; GeneID:3866352; lipoprotein LolB precursor | 1.03 | 0.269 |
| SG1126; GI:85059104; GeneID:3867554; glutamine amidotransferase HisH | 1.03 | 0.264 |
| SG0550; GI:85058528; GeneID:3867602; putative invasion protein | 1.03 | 0.414 |
| SG0271; GI:85058249; GeneID:3867911; topoisomerase IV subunit B | 1.03 | 0.267 |
| SG1712; GI:85059690; GeneID:3866458; coproporphyrinogen III oxidase | 1.03 | 0.312 |
| SG2161; GI:85060139; GeneID:3868270; acetylornithine deacetylase | 1.03 | 0.244 |
| SG0177; GI:85058155; GeneID:3868504; putative tagatose 6-phosphate kinase | 1.03 | 0.486 |
| SG1422; GI:85059400; GeneID:3866983; phenylalanyl-tRNA synthase alpha subunit | 1.03 | 0.285 |
| SG1883; GI:85059861; GeneID:3866357; ethanolamine utilization protein EutJ | 1.03 | 0.399 |
| SG1142; GI:85059120; GeneID:3867133; hypothetical protein | 1.03 | 0.341 |
| SG0696; GI:85058674; GeneID:3868613; hypothetical protein | 1.03 | 0.367 |
| SG1268; GI:85059246; GeneID:3867875; pyruvate kinase II | 1.03 | 0.259 |
| SG0245; GI:85058223; GeneID:3868297; putative phage tail tube protein | 1.03 | 0.386 |
| SG0895; GI:85058873; GeneID:3868343; putative galactokinase | 1.03 | 0.275 |
| SG0146; GI:85058124; GeneID:3868512; putative oligopeptide ABC transporter | 1.03 | 0.174 |
| SG0304; GI:85058282; GeneID:3868572; phage suppressor of F exclusion | 1.03 | 0.220 |
| SG1767; GI:85059745; GeneID:3866926; enhanced serine sensitivity protein | 1.03 | 0.273 |
| SG0909; GI:85058887; GeneID:3868357; molybdopterin biosynthesis protein C | 1.03 | 0.283 |
| SG1981; GI:85059959; GeneID:3866789; DNA mismatch repair protein | 1.03 | 0.167 |
| SG2073; GI:85060051; GeneID:3868104; hypothetical protein | 1.03 | 0.281 |
| SG1876; GI:85059854; GeneID:3866350; peptide chain release factor RF-1 | 1.03 | 0.262 |
| SG0818; GI:85058796; GeneID:3868027; hypothetical protein | 1.03 | 0.375 |
| SG0264; GI:85058242; GeneID:3867904; hypothetical protein | 1.03 | 0.243 |
| SG1837; GI:85059815; GeneID:3868630; putative mannonate oxidoreductase | 1.03 | 0.386 |
| SG0074; GI:85058052; GeneID:3867498; universal stress protein A | 1.03 | 0.211 |
| GI:125470017; GI:125470017; gp01; possible TerS subunit; similar to gp03 of P22 | 1.03 | 0.425 |
| SG0803; GI:85058781; GeneID:3868486; leucyl-tRNA synthase | 1.03 | 0.272 |
| SG2276; GI:85060254; GeneID:3866301; 50S ribosomal protein L23 | 1.03 | 0.218 |
| SG0827; GI:85058805; GeneID:3868769; hypothetical protein | 1.03 | 0.442 |
| SG0005; GI:85057983; GeneID:3867662; putative galactonate operon transcriptional | 1.03 | 0.304 |
| SG0203; GI:85058181; GeneID:3868074; hypothetical protein | 1.03 | 0.299 |
| SG1968; GI:85059946; GeneID:3866776; putative phage anti-termination protein | 1.03 | 0.258 |
| SG0259; GI:85058237; GeneID:3867899; hypothetical protein | 1.03 | 0.202 |
| SG2098; GI:85060076; GeneID:3866473; hypothetical protein | 1.03 | 0.263 |
| SG0571; GI:85058549; GeneID:3867685; type III secretion apparatus SpaR | 1.03 | 0.314 |
| SG0497; GI:85058475; GeneID:3868276; hypothetical protein | 1.03 | 0.321 |
| SG1271; GI:85059249; GeneID:3867878; putative transcriptional regulator | 1.03 | 0.249 |
| SG0496; GI:85058474; GeneID:3868275; hypothetical protein | 1.03 | 0.270 |
| SG0547; GI:85058525; GeneID:3868663; 16S rRNA processing protein RimM | 1.03 | 0.210 |
| SG0826; GI:85058804; GeneID:3868768; hypothetical protein | 1.03 | 0.349 |
| SG0136; GI:85058114; GeneID:3867970; putative thiamine biosynthesis protein ThiH | 1.03 | 0.381 |
| SG0927; GI:85058905; GeneID:3868375; putrescine ABC transporter permease component | 1.03 | 0.273 |
| SG1799; GI:85059777; GeneID:3866802; hypothetical protein | 1.03 | 0.214 |
| SG0196; GI:85058174; GeneID:3866635; hypothetical protein | 1.03 | 0.284 |
| SG0122; GI:85058100; GeneID:3867956; hypothetical protein | 1.03 | 0.245 |
| SG0159; GI:85058137; GeneID:3868525; ribonuclease G | 1.03 | 0.277 |
| SG2100; GI:85060078; GeneID:3866475; hypothetical protein | 1.03 | 0.283 |
| SG2067; GI:85060045; GeneID:3868098; flagellar transcriptional activator FlhC | 1.03 | 0.202 |
| SG1449; GI:85059427; GeneID:3867266; endonuclease III | 1.03 | 0.242 |
| SG0214; GI:85058192; GeneID:3868085; hypothetical protein | 1.03 | 0.258 |
| SG2058; GI:85060036; GeneID:3866510; flagellar biosynthetic protein FliR | 1.03 | 0.274 |
| SG2156; GI:85060134; GeneID:3868265; hypothetical protein | 1.03 | 0.178 |
| GI:125470061; GI:125470061; gp49; hypothetical protein | 1.03 | 0.233 |
| SG1734; GI:85059712; GeneID:3866479; putative phosphate ABC transporter ATP-binding | 1.03 | 0.220 |
| GI:125470020; GI:125470020; gp04; possible scaffolding protein; similar to HkbR of | 1.03 | 0.321 |
| SG2153; GI:85060131; GeneID:3868262; glutamate racemase | 1.03 | 0.271 |
| SG0787; GI:85058765; GeneID:3868470; hypothetical protein | 1.03 | 0.320 |
| SG0521; GI:85058499; GeneID:3868222; ATP-sulfurylase subunit 2 | 1.03 | 0.147 |
| SG0492; GI:85058470; GeneID:3868271; dnaK suppressor protein | 1.03 | 0.218 |
| SG0176; GI:85058154; GeneID:3868503; putative xylose ABC transporter periplasmic | 1.03 | 0.245 |
| SG0970; GI:85058948; GeneID:3867097; methionyl-tRNA synthase | 1.03 | 0.283 |
| SG0515; GI:85058493; GeneID:3868216; putative 6-pyruvoyl tetrahydrobiopterin | 1.03 | 0.216 |
| SG0481; GI:85058459; GeneID:3868695; hypothetical protein | 1.03 | 0.182 |
| SG1619; GI:85059597; GeneID:3867797; pseudouridylate synthase I | 1.03 | 0.212 |
| SG0690; GI:85058668; GeneID:3868607; hypothetical protein | 1.03 | 0.250 |
| SG0792; GI:85058770; GeneID:3868475; lipoate synthase | 1.03 | 0.248 |
| SG1969; GI:85059947; GeneID:3866777; hypothetical protein | 1.03 | 0.159 |
| SG1507; GI:85059485; GeneID:3868233; peptide ABC transporter permease component SapB | 1.03 | 0.185 |
| SG0951; GI:85058929; GeneID:3868438; hypothetical protein | 1.03 | 0.240 |
| SG0444; GI:85058422; GeneID:3867419; UDP-N-acetylmuramoylalanyl-D-glutamate-2,6- | 1.03 | 0.210 |
| SG2412; GI:85060390; GeneID:3868411; ATP synthase alpha subunit | 1.03 | 0.162 |
| SG2358; GI:85060336; GeneID:3866815; phage protease | 1.03 | 0.304 |
| SG1089; GI:85059067; GeneID:3868166; hypothetical protein | 1.03 | 0.167 |
| SG0789; GI:85058767; GeneID:3868472; putative phage base plate protein | 1.03 | 0.243 |
| SG2139; GI:85060117; GeneID:3867300; putative regulator | 1.03 | 0.192 |
| SG1434; GI:85059412; GeneID:3866937; hypothetical protein | 1.03 | 0.229 |
| SG1508; GI:85059486; GeneID:3868234; hypothetical protein | 1.03 | 0.218 |
| SG0957; GI:85058935; GeneID:3868444; GTP cyclohydrolase I | 1.03 | 0.211 |
| SG2319; GI:85060297; GeneID:3867304; two-component regulatory protein | 1.03 | 0.209 |
| SG0729; GI:85058707; GeneID:3867706; hypothetical protein | 1.03 | 0.345 |
| SG1143; GI:85059121; GeneID:3867134; hypothetical protein | 1.03 | 0.137 |
| GI:125470041; GI:125470041; gp26; hypothetical protein; possible two-component | 1.03 | 0.206 |
| SG0899; GI:85058877; GeneID:3868347; D-alanine-D-alanine ligase A | 1.03 | 0.227 |
| SG1473; GI:85059451; GeneID:3866898; hypothetical protein | 1.03 | 0.249 |
| SG1144; GI:85059122; GeneID:3867135; hypothetical protein | 1.02 | 0.271 |
| SG2186; GI:85060164; GeneID:3866994; glutaredoxin 3 | 1.02 | 0.227 |
| SG0012; GI:85057990; GeneID:3867669; glycyl-tRNA synthase beta subunit | 1.02 | 0.214 |
| SG0465; GI:85058443; GeneID:3868205; aromatic amino acid transport protein | 1.02 | 0.224 |
| SG1832; GI:85059810; GeneID:3868625; hypothetical protein | 1.02 | 0.314 |
| SG0600; GI:85058578; GeneID:3866668; gamma-glutamate kinase | 1.02 | 0.208 |
| SG0075; GI:85058053; GeneID:3867499; low-affinity inorganic phosphate transport | 1.02 | 0.249 |
| SG0500; GI:85058478; GeneID:3868279; glutamate-1-semialdehyde aminotransferase | 1.02 | 0.199 |
| SG1819; GI:85059797; GeneID:3866646; hypothetical protein | 1.02 | 0.212 |
| SG2192; GI:85060170; GeneID:3867000; hypothetical protein | 1.02 | 0.215 |
| SG2064; GI:85060042; GeneID:3868095; chemotaxis protein CheZ | 1.02 | 0.303 |
| SG1982; GI:85059960; GeneID:3866790; putative aldo/keto reductase | 1.02 | 0.233 |
| SG0615; GI:85058593; GeneID:3867589; hypothetical protein | 1.02 | 0.248 |
| SG1359; GI:85059337; GeneID:3867331; hypothetical protein | 1.02 | 0.181 |
| SG1568; GI:85059546; GeneID:3866681; putative phage tail fiber component | 1.02 | 0.229 |
| SG1668; GI:85059646; GeneID:3868532; hypothetical protein | 1.02 | 0.326 |
| SG0847; GI:85058825; GeneID:3867805; hypothetical protein | 1.02 | 0.269 |
| SG0320; GI:85058298; GeneID:3867392; hypothetical protein | 1.02 | 0.205 |
| SG0205; GI:85058183; GeneID:3868076; putative ABC transporter ATP-binding component | 1.02 | 0.209 |
| SG0357; GI:85058335; GeneID:3867469; UDP-N-acetylmuramate:L-alanyl-gamma-D-glutamyl- | 1.02 | 0.231 |
| SG2309; GI:85060287; GeneID:3866315; shikimate kinase I | 1.02 | 0.185 |
| SG0838; GI:85058816; GeneID:3868780; hypothetical protein | 1.02 | 0.281 |
| SG2043; GI:85060021; GeneID:3866754; hypothetical protein | 1.02 | 0.271 |
| SG1593; GI:85059571; GeneID:3867888; NADH dehydrogenase I subunit J | 1.02 | 0.190 |
| SG0856; GI:85058834; GeneID:3867814; putative phage tail fiber protein | 1.02 | 0.340 |
| SG1983; GI:85059961; GeneID:3866791; galactose operon repressor | 1.02 | 0.198 |
| SG1963; GI:85059941; GeneID:3866221; hypothetical protein | 1.02 | 0.259 |
| SG0724; GI:85058702; GeneID:3868744; phage terminase large subunit | 1.02 | 0.288 |
| SG1900; GI:85059878; GeneID:3867773; putative acetyltransferase | 1.02 | 0.224 |
| SG2140; GI:85060118; GeneID:3867301; putative transposase | 1.02 | 0.187 |
| SG1886; GI:85059864; GeneID:3866360; periplasmic trehalase | 1.02 | 0.212 |
| SG1794; GI:85059772; GeneID:3866797; L-aspartate oxidase | 1.02 | 0.184 |
| SG0944; GI:85058922; GeneID:3868431; hypothetical protein | 1.02 | 0.262 |
| SG2183; GI:85060161; GeneID:3866991; serine acetyltransferase | 1.02 | 0.172 |
| GI:125470058; GI:125470058; gp46; possible partitioning protein; similar to ParA from | 1.02 | 0.290 |
| SG1552; GI:85059530; GeneID:3867192; hypothetical protein | 1.02 | 0.171 |
| GI:125470053; GI:125470053; gp40; phage-related protein; similar to gp04 of pSG3, | 1.02 | 0.203 |
| SG0580; GI:85058558; GeneID:3867694; chorismate mutase P/prephenate dehydratase | 1.02 | 0.197 |
| SG0051; GI:85058029; GeneID:3867436; flagellar assembly protein FliH | 1.02 | 0.210 |
| SG1892; GI:85059870; GeneID:3867765; phage integrase | 1.02 | 0.101 |
| SG0312; GI:85058290; GeneID:3868580; phosphatidylserine decarboxylase | 1.02 | 0.198 |
| SG0530; GI:85058508; GeneID:3868231; lipoprotein | 1.02 | 0.214 |
| SG1870; GI:85059848; GeneID:3866344; hypothetical protein | 1.02 | 0.184 |
| SG0648; GI:85058626; GeneID:3867994; protein-export membrane protein SecD | 1.02 | 0.200 |
| SGP2_0011; GI:85060477; GeneID:3866589; hypothetical protein | 1.02 | 0.111 |
| SG0993; GI:85058971; GeneID:3866886; 30S ribosomal protein S1 | 1.02 | 0.154 |
| SG0338; GI:85058316; GeneID:3868117; GTP-binding protein | 1.02 | 0.167 |
| SG1788; GI:85059766; GeneID:3866532; signal peptidase I | 1.02 | 0.184 |
| SG0425; GI:85058403; GeneID:3867536; pyridoxal phosphate biosynthetic protein | 1.02 | 0.200 |
| SG0864; GI:85058842; GeneID:3868312; hypothetical protein | 1.02 | 0.165 |
| SG2166; GI:85060144; GeneID:3867940; primosomal protein N' factor | 1.02 | 0.173 |
| SG1550; GI:85059528; GeneID:3867190; transcriptional regulator | 1.02 | 0.193 |
| SG2116; GI:85060094; GeneID:3866257; aspartate carbamoyltransferase regulatory | 1.02 | 0.161 |
| SG0324; GI:85058302; GeneID:3867396; hypothetical protein | 1.02 | 0.159 |
| SG0541; GI:85058519; GeneID:3868657; gamma-glutamylcysteine synthase | 1.02 | 0.173 |
| SG2194; GI:85060172; GeneID:3867002; lipopolysaccharide heptosyltransferase II | 1.02 | 0.189 |
| SG1281; GI:85059259; GeneID:3866391; putative secreted effector protein | 1.02 | 0.141 |
| SG0355; GI:85058333; GeneID:3867467; inorganic pyrophosphatase | 1.02 | 0.167 |
| SG0315; GI:85058293; GeneID:3868583; hypothetical protein | 1.02 | 0.234 |
| SG0505; GI:85058483; GeneID:3868284; global stress requirement protein GsrA | 1.02 | 0.155 |
| SG1691; GI:85059669; GeneID:3866274; hypothetical protein | 1.02 | 0.179 |
| SG1029; GI:85059007; GeneID:3867215; putative transposase | 1.02 | 0.160 |
| SG1170; GI:85059148; GeneID:3866240; transcription regulator | 1.02 | 0.140 |
| SG2091; GI:85060069; GeneID:3866466; type III secretion apparatus InvG | 1.02 | 0.195 |
| SG0244; GI:85058222; GeneID:3868296; putative phage capsid portal protein | 1.02 | 0.203 |
| SG0067; GI:85058045; GeneID:3867491; ketodeoxygluconokinase | 1.02 | 0.208 |
| SG1804; GI:85059782; GeneID:3866807; small protein B | 1.02 | 0.176 |
| SG0326; GI:85058304; GeneID:3867398; hypothetical protein | 1.02 | 0.287 |
| SG1265; GI:85059243; GeneID:3867872; lipid A biosynthesis acyltransferase | 1.02 | 0.156 |
| SG0476; GI:85058454; GeneID:3868690; putative chitinase | 1.02 | 0.194 |
| SG2045; GI:85060023; GeneID:3866497; hypothetical protein | 1.02 | 0.127 |
| SG1305; GI:85059283; GeneID:3867120; putative type III secretion apparatus | 1.02 | 0.192 |
| SG0211; GI:85058189; GeneID:3868082; UDP-N-acetylglucosamine | 1.02 | 0.194 |
| SG1406; GI:85059384; GeneID:3867046; putative protease | 1.02 | 0.192 |
| SG2298; GI:85060276; GeneID:3866304; hypothetical protein | 1.02 | 0.176 |
| SG1664; GI:85059642; GeneID:3868528; hypothetical protein | 1.02 | 0.191 |
| SG1458; GI:85059436; GeneID:3867275; putative secretion protein | 1.02 | 0.150 |
| SG2308; GI:85060286; GeneID:3866314; 3-dehydroquinate synthase | 1.02 | 0.178 |
| SG0691; GI:85058669; GeneID:3868608; recombination protein RecR | 1.02 | 0.199 |
| SG0300; GI:85058278; GeneID:3868568; putative transcriptional regulator | 1.02 | 0.179 |
| SG1920; GI:85059898; GeneID:3867653; prolyl-tRNA synthase | 1.02 | 0.196 |
| SG0564; GI:85058542; GeneID:3867616; type III secretion apparatus InvB | 1.02 | 0.204 |
| SG0356; GI:85058334; GeneID:3867468; fructose-1,6-bisphosphatase | 1.02 | 0.164 |
| SG0036; GI:85058014; GeneID:3867460; flagellar L-ring protein precursor FlgH | 1.02 | 0.229 |
| SG0255; GI:85058233; GeneID:3868307; hypothetical protein | 1.02 | 0.158 |
| SG1701; GI:85059679; GeneID:3866447; PTS system phosphocarrier protein HPr | 1.02 | 0.178 |
| SG1492; GI:85059470; GeneID:3868179; acyl carrier protein phosphodiesterase | 1.02 | 0.138 |
| SG1803; GI:85059781; GeneID:3866806; hypothetical protein | 1.02 | 0.144 |
| SG0593; GI:85058571; GeneID:3866661; hypothetical protein | 1.02 | 0.119 |
| SG1621; GI:85059599; GeneID:3867799; erythronate-4-phosphate dehydrogenase | 1.02 | 0.147 |
| SG1150; GI:85059128; GeneID:3867141; hypothetical protein | 1.02 | 0.236 |
| SG0447; GI:85058425; GeneID:3867422; UDP-N-acetylmuramoylalanine-D-glutamate ligase | 1.02 | 0.146 |
| SG0719; GI:85058697; GeneID:3868739; phage tail sheath protein | 1.02 | 0.220 |
| SG0555; GI:85058533; GeneID:3867607; putative type III secretion apparatus | 1.02 | 0.206 |
| SGP2_0007; GI:85060473; GeneID:3866608; hypothetical protein | 1.02 | 0.084 |
| SG0181; GI:85058159; GeneID:3866620; L-fucose isomerase | 1.02 | 0.195 |
| SG1596; GI:85059574; GeneID:3867891; NADH dehydrogenase I subunit G | 1.02 | 0.147 |
| SG1662; GI:85059640; GeneID:3868526; hypothetical protein | 1.02 | 0.168 |
| SG2000; GI:85059978; GeneID:3867081; glycine dehydrogenase | 1.02 | 0.198 |
| SG0052; GI:85058030; GeneID:3867437; flagellar motor switch protein FliG | 1.02 | 0.150 |
| SG0178; GI:85058156; GeneID:3868505; L-fucose operon regulator | 1.02 | 0.179 |
| SG2268; GI:85060246; GeneID:3866293; 50S ribosomal protein L14 | 1.02 | 0.106 |
| SG1067; GI:85059045; GeneID:3866707; putative deoxyribonuclease | 1.02 | 0.131 |
| SG1511; GI:85059489; GeneID:3868237; peptide ABC transporter ATP-binding component | 1.02 | 0.151 |
| SG0915; GI:85058893; GeneID:3868363; putative amino acid ABC transporter ATP-binding | 1.02 | 0.178 |
| SG0422; GI:85058400; GeneID:3867533; bis(5'-nucleosyl)-tetraphosphatase | 1.02 | 0.163 |
| SG2024; GI:85060002; GeneID:3868803; hypothetical protein | 1.02 | 0.140 |
| SG1235; GI:85059214; GeneID:3866758; hypothetical protein | 1.02 | 0.055 |
| SG0213; GI:85058191; GeneID:3868084; serine protease | 1.02 | 0.129 |
| SG0882; GI:85058860; GeneID:3868330; hypothetical protein | 1.02 | 0.144 |
| SG1383; GI:85059361; GeneID:3867258; hypothetical protein | 1.02 | 0.146 |
| SG2120; GI:85060098; GeneID:3866261; putative type II restriction enzyme | 1.02 | 0.168 |
| SG1985; GI:85059963; GeneID:3866793; formate/nitrite transport protein | 1.02 | 0.147 |
| SG1013; GI:85058991; GeneID:3866377; hypothetical protein | 1.02 | 0.142 |
| SG0314; GI:85058292; GeneID:3868582; putative oligoribonuclease | 1.02 | 0.138 |
| SG2400; GI:85060378; GeneID:3868399; lysR-family transcriptional regulator | 1.02 | 0.143 |
| SG0293; GI:85058271; GeneID:3868600; hypothetical protein | 1.02 | 0.201 |
| SG0595; GI:85058573; GeneID:3866663; phosphoheptose isomerase | 1.01 | 0.111 |
| SG0823; GI:85058801; GeneID:3868765; hypothetical protein | 1.01 | 0.186 |
| SG2376; GI:85060354; GeneID:3867347; hypothetical protein | 1.01 | 0.139 |
| SG0151; GI:85058129; GeneID:3868517; ribosomal protein L11 methyltransferase | 1.01 | 0.127 |
| SG2238; GI:85060216; GeneID:3867366; hypothetical protein | 1.01 | 0.115 |
| SG1129; GI:85059107; GeneID:3867557; histidinol dehydrogenase HisD | 1.01 | 0.115 |
| SG0327; GI:85058305; GeneID:3867399; potassium-transporting ATPase C chain | 1.01 | 0.124 |
| SG0701; GI:85058679; GeneID:3868618; phosphoribosylaminoimidazole carboxylase ATPase | 1.01 | 0.124 |
| SG1835; GI:85059813; GeneID:3868628; putative transposase | 1.01 | 0.112 |
| SG0463; GI:85058441; GeneID:3868203; putative prelipin peptidase dependent protein | 1.01 | 0.110 |
| SG0236; GI:85058214; GeneID:3868068; hypothetical protein | 1.01 | 0.137 |
| SG1530; GI:85059508; GeneID:3867229; hypothetical protein | 1.01 | 0.099 |
| SG1105; GI:85059083; GeneID:3868715; cytochrome-related ABC transporter ATP-binding | 1.01 | 0.103 |
| SG0755; GI:85058733; GeneID:3867732; hypothetical protein | 1.01 | 0.138 |
| SG0845; GI:85058823; GeneID:3867803; phage tail sheath protein | 1.01 | 0.163 |
| SG0078; GI:85058056; GeneID:3867502; hypothetical protein | 1.01 | 0.114 |
| SG2256; GI:85060234; GeneID:3866733; 30S ribosomal protein S13 | 1.01 | 0.092 |
| GI:125470062; GI:125470062; gp50; possible HU-like DNA binding protein; similar to | 1.01 | 0.100 |
| SG0967; GI:85058945; GeneID:3867094; NAD-linked malate dehydrogenase | 1.01 | 0.109 |
| SG2084; GI:85060062; GeneID:3866459; type III secretion apparatus SpaP | 1.01 | 0.097 |
| SG0807; GI:85058785; GeneID:3868016; apolipoprotein N-acyltransferase | 1.01 | 0.058 |
| SG0822; GI:85058800; GeneID:3868031; hypothetical protein | 1.01 | 0.155 |
| SG2154; GI:85060132; GeneID:3868263; tRNA (uracil-5)-methyltransferase | 1.01 | 0.113 |
| SG2311; GI:85060289; GeneID:3866317; penicillin-binding protein 1A | 1.01 | 0.110 |
| SG0360; GI:85058338; GeneID:3867472; hypothetical protein | 1.01 | 0.000 |
| SG1329; GI:85059307; GeneID:3867524; para-aminobenzoate synthase component I | 1.01 | 0.090 |
| SG0454; GI:85058432; GeneID:3868194; UDP-3-O-[3-hydroxymyristoyl] N-acetylglucosamine | 1.01 | 0.091 |
| SG1673; GI:85059651; GeneID:3868537; putative oxidoreductase | 1.01 | 0.122 |
| SG1746; GI:85059724; GeneID:3866491; putative PTS system galactitol-specific IIA | 1.01 | 0.140 |
| SG1079; GI:85059057; GeneID:3868156; putative NAD-dependent protein deacetylase | 1.01 | 0.106 |
| SG0248; GI:85058226; GeneID:3868300; hypothetical protein | 1.01 | 0.106 |
| SG1475; GI:85059453; GeneID:3866900; hypothetical protein | 1.01 | 0.132 |
| SG0040; GI:85058018; GeneID:3867464; flagellar hook-associated protein 3 FlgL | 1.01 | 0.088 |
| SG0090; GI:85058068; GeneID:3867750; hypothetical protein | 1.01 | 0.131 |
| SG0734; GI:85058712; GeneID:3867711; hypothetical protein | 1.01 | 0.129 |
| SG1591; GI:85059569; GeneID:3867886; NADH dehydrogenase I subunit L | 1.01 | 0.068 |
| SG1183; GI:85059161; GeneID:3868554; hypothetical protein | 1.01 | 0.060 |
| SG0191; GI:85058169; GeneID:3866630; hypothetical protein | 1.01 | 0.100 |
| SG2199; GI:85060177; GeneID:3867007; hypothetical protein | 1.01 | 0.107 |
| SG2347; GI:85060325; GeneID:3867175; adenylate cyclase | 1.01 | 0.066 |
| SG2307; GI:85060285; GeneID:3866313; hypothetical protein | 1.01 | 0.089 |
| SG0906; GI:85058884; GeneID:3868354; dethiobiotin synthase | 1.01 | 0.078 |
| SG1867; GI:85059845; GeneID:3867065; hypothetical protein | 1.01 | 0.092 |
| SGP3_0003; GI:85060493; GeneID:3866614; hypothetical protein | 1.01 | 0.094 |
| SG1735; GI:85059713; GeneID:3866480; putative phosphate ABC transporter permease | 1.01 | 0.090 |
| SG1236; GI:85059213; GeneID:3866759; phage outer membrane protein | 1.01 | 0.107 |
| SG1506; GI:85059484; GeneID:3868232; peptide ABC transporter periplasmic component | 1.01 | 0.086 |
| SG1398; GI:85059376; GeneID:3867038; tryptophan synthase beta subunit TrpB | 1.01 | 0.086 |
| SG2231; GI:85060209; GeneID:3868815; two-component nitrogen regulation protein | 1.01 | 0.077 |
| SG0935; GI:85058913; GeneID:3868383; hypothetical protein | 1.01 | 0.100 |
| SG1850; GI:85059828; GeneID:3867048; hypothetical protein | 1.01 | 0.093 |
| SG1282; GI:85059260; GeneID:3866392; type III secretion apparatus | 1.01 | 0.096 |
| SG0731; GI:85058709; GeneID:3867708; hypothetical protein | 1.01 | 0.093 |
| SG1802; GI:85059780; GeneID:3866805; hypothetical protein | 1.01 | 0.056 |
| SG0173; GI:85058151; GeneID:3868500; putative transcriptional regulator | 1.01 | 0.083 |
| SG1012; GI:85058990; GeneID:3866376; putative phage DNA adenine methylase | 1.01 | 0.086 |
| SG1906; GI:85059884; GeneID:3867779; glycine betaine/L-proline ABC transporter | 1.01 | 0.107 |
| SG1110; GI:85059088; GeneID:3868720; putative ATPase protein | 1.01 | 0.076 |
| SG0462; GI:85058440; GeneID:3868202; hypothetical protein | 1.01 | 0.081 |
| SG1739; GI:85059717; GeneID:3866484; putative hydoxyethylthiazole kinase | 1.01 | 0.006 |
| SG0754; GI:85058732; GeneID:3867731; hypothetical protein | 1.01 | 0.093 |
| SG0420; GI:85058398; GeneID:3867531; carbamoyl-phosphate synthase large subunit | 1.01 | 0.066 |
| SG2277; GI:85060255; GeneID:3866302; 50S ribosomal protein L4 | 1.01 | 0.064 |
| SG2323; GI:85060301; GeneID:3867308; sucrose phosphorylase | 1.01 | 0.097 |
| SG1050; GI:85059028; GeneID:3866962; DNA-damage-inducible protein I | 1.01 | 0.042 |
| SG0045; GI:85058023; GeneID:3867430; flagellar motor switch protein FliN | 1.01 | 0.073 |
| SG0216; GI:85058194; GeneID:3868087; 50S ribosomal protein L13 | 1.01 | 0.062 |
| SG2027; GI:85060005; GeneID:3866738; hypothetical protein | 1.01 | 0.046 |
| SG0031; GI:85058009; GeneID:3867455; flagellar basal-body rod protein FlgC | 1.01 | 0.064 |
| GI:125470057; GI:125470057; gp45; possible replication protein; similar to RepA from | 1.01 | 0.091 |
| SG1769; GI:85059747; GeneID:3866928; putative aminotransferase | 1.01 | 0.022 |
| SG0855; GI:85058833; GeneID:3867813; putative phage baseplate assembly protein | 1.01 | 0.090 |
| SG0283; GI:85058261; GeneID:3868590; putative oxidoreductase | 1.01 | 0.068 |
| SG0237; GI:85058215; GeneID:3868069; hypothetical protein | 1.01 | 0.069 |
| SG1857; GI:85059835; GeneID:3867055; putative propanediol utilization protein | 1.01 | 0.076 |
| SGP2_0001; GI:85060467; GeneID:3866602; hypothetical protein | 1.01 | 0.077 |
| SG1667; GI:85059645; GeneID:3868531; hypothetical protein | 1.01 | 0.064 |
| SG0398; GI:85058376; GeneID:3867567; phosphoserine phosphatase | 1.01 | 0.057 |
| SG1248; GI:85059226; GeneID:3866771; arginyl-tRNA synthase | 1.01 | 0.047 |
| SG0868; GI:85058846; GeneID:3868316; deoxyribodipyrimidine photolyase | 1.01 | 0.045 |
| SG2053; GI:85060031; GeneID:3866505; flagellar motor switch protein FliM | 1.01 | 0.066 |
| SG0006; GI:85057984; GeneID:3867663; putative effector protein | 1.01 | 0.059 |
| SG1343; GI:85059321; GeneID:3866414; putative sodium-hydrogen antiporter | 1.01 | 0.046 |
| SG1404; GI:85059382; GeneID:3867044; hypothetical protein | 1.01 | 0.058 |
| SG1754; GI:85059732; GeneID:3866913; hypothetical protein | 1.01 | 0.072 |
| SG2251; GI:85060229; GeneID:3866728; hypothetical protein | 1.01 | 0.039 |
| SG2057; GI:85060035; GeneID:3866509; flagellar biosynthetic protein FliQ | 1.01 | 0.065 |
| SG2236; GI:85060214; GeneID:3867364; thiol:disulfide interchange protein | 1.00 | 0.039 |
| SG1152; GI:85059130; GeneID:3867143; hypothetical protein | 1.00 | 0.039 |
| SG0689; GI:85058667; GeneID:3868606; DNA polymerase III gamma and tau subunits | 1.00 | 0.043 |
| SG0730; GI:85058708; GeneID:3867707; putative DNA replication protein | 1.00 | 0.044 |
| SGP1_0049; GI:85060460; GeneID:3866588; hypothetical protein | 1.00 | 0.058 |
| SG2234; GI:85060212; GeneID:3867362; hypothetical protein | 1.00 | 0.039 |
| SG1244; GI:85059222; GeneID:3866767; hypothetical protein | 1.00 | 0.052 |
| SG2335; GI:85060313; GeneID:3867320; hypothetical protein | 1.00 | 0.042 |
| SG0105; GI:85058083; GeneID:3867978; hypothetical protein | 1.00 | 0.054 |
| SG2351; GI:85060329; GeneID:3867179; putative phage tail protein | 1.00 | 0.044 |
| SG2068; GI:85060046; GeneID:3868099; flagellar transcriptional activator FlhD | 1.00 | 0.028 |
| SG1758; GI:85059736; GeneID:3866917; hypothetical protein | 1.00 | 0.031 |
| SG0659; GI:85058637; GeneID:3868005; thiamine biosynthesis protein ThiI | 1.00 | 0.030 |
| SG1879; GI:85059857; GeneID:3866353; 4-diphosphocytidyl-2-C-methyl-D-erythritol | 1.00 | 0.035 |
| SG0959; GI:85058937; GeneID:3868446; two-component system response regulator | 1.00 | 0.031 |
| SG0199; GI:85058177; GeneID:3868070; RNA polymerase sigma-54 factor RpoN | 1.00 | 0.029 |
| SG1710; GI:85059688; GeneID:3866456; hypothetical protein | 1.00 | 0.073 |
| SG2262; GI:85060240; GeneID:3866287; 50S ribosomal protein L18 | 1.00 | 0.027 |
| SG0080; GI:85058058; GeneID:3867504; hypothetical protein | 1.00 | 0.023 |
| SG1605; GI:85059583; GeneID:3867783; hypothetical protein | 1.00 | 0.034 |
| SG1212; GI:85059190; GeneID:3866851; hypothetical protein | 1.00 | 0.056 |
| SG0028; GI:85058006; GeneID:3867452; negative regulator of flagellin synthesis FlgM | 1.00 | 0.037 |
| SG2026; GI:85060004; GeneID:3866737; putative L-rhamnose operon transcriptional | 1.00 | 0.032 |
| SG1307; GI:85059285; GeneID:3867122; putative type III secretion apparatus | 1.00 | 0.027 |
| SG0679; GI:85058657; GeneID:3868146; nitrogen regulatory protein P-II | 1.00 | 0.025 |
| SG1896; GI:85059874; GeneID:3867769; putative transport protein | 1.00 | 0.024 |
| SG1848; GI:85059826; GeneID:3868641; putative pyrrolidone-carboxylate peptidase | 1.00 | 0.016 |
| SG0635; GI:85058613; GeneID:3868454; hypothetical protein | 1.00 | 0.018 |
| SG0224; GI:85058202; GeneID:3868056; putative phosphoheptose isomerase | 1.00 | 0.020 |
| SG0840; GI:85058818; GeneID:3868782; hypothetical protein | 1.00 | 0.035 |
| SG0720; GI:85058698; GeneID:3868740; hypothetical protein | 1.00 | 0.029 |
| SG2167; GI:85060145; GeneID:3867941; cell division protein | 1.00 | 0.022 |
| SG2198; GI:85060176; GeneID:3867006; putative lipid A core:surface polymer ligase | 1.00 | 0.008 |
| SG0809; GI:85058787; GeneID:3868018; hypothetical protein | 1.00 | 0.011 |
| SG1724; GI:85059702; GeneID:3866863; phosphoribosylaminoimidazole-succinocarboxamide | 1.00 | 0.020 |
| SG0070; GI:85058048; GeneID:3867494; putative transposase | 1.00 | 0.019 |
| SG0144; GI:85058122; GeneID:3868510; putative transcriptional regulator | 1.00 | 0.012 |
| SG2385; GI:85060363; GeneID:3867356; undecaprenyl-phosphate | 1.00 | 0.023 |
| SG0726; GI:85058704; GeneID:3867703; hypothetical protein | 1.00 | 0.021 |
| SG0569; GI:85058547; GeneID:3867621; type III secretion apparatus SpaP | 1.00 | 0.026 |
| SG0439; GI:85058417; GeneID:3867414; hypothetical protein | 1.00 | 0.022 |
| SG0345; GI:85058323; GeneID:3868124; 30S ribosomal protein S6 | 1.00 | 0.022 |
| SG1114; GI:85059092; GeneID:3868724; mannose-1-phosphate guanylyltransferase | 1.00 | 0.014 |
| GI:125470048; GI:125470048; gp33; integrase-like protein; similar to gp12 of pSG3 and | 1.00 | 0.026 |
| SG0852; GI:85058830; GeneID:3867810; putative phage regulatory protein | 1.00 | 0.005 |
| SG2419; GI:85060397; GeneID:3868418; putative phosphate ABC transporter permease | 1.00 | 0.006 |
| SG0751; GI:85058729; GeneID:3867728; hypothetical protein | 1.00 | 0.008 |
| SG0251; GI:85058229; GeneID:3868303; RNA polymerase sigma-70 factor RpoD | 1.00 | 0.012 |
| SG0561; GI:85058539; GeneID:3867613; type III secretion apparatus InvG | 1.00 | 0.005 |
| SG1995; GI:85059973; GeneID:3867076; hypothetical protein | 1.00 | 0.042 |
| SG2017; GI:85059995; GeneID:3868796; agmatinase | 1.00 | 0.001 |
| SG0892; GI:85058870; GeneID:3868340; hypothetical protein | 1.00 | 0.011 |
| SG1510; GI:85059488; GeneID:3868236; peptide ABC transporter permease component | 1.00 | 0.001 |
| SG2285; GI:85060263; GeneID:3866427; 30S ribosomal protein S7 | 1.00 | 0.017 |
| SG0325; GI:85058303; GeneID:3867397; hypothetical protein | 1.00 | 0.011 |
| SG1537; GI:85059515; GeneID:3867236; putative two-component sensor protein | 1.00 | 0.008 |
| SG2034; GI:85060012; GeneID:3866745; putative methyltransferase | 1.00 | 0.000 |
| SG1775; GI:85059753; GeneID:3866519; serine hydroxymethyltransferase | 1.00 | 0.000 |
| SG0112; GI:85058090; GeneID:3867985; sec-independent protein translocase protein | 1.00 | 0.005 |
| SG0198; GI:85058176; GeneID:3866637; putative sigma-54 modulation protein | -1.00 | 0.021 |
| SG2042; GI:85060020; GeneID:3866753; putative PTS system IIB component | -1.00 | 0.027 |
| SG1379; GI:85059357; GeneID:3867254; hypothetical protein | -1.00 | 0.008 |
| SG1016; GI:85058994; GeneID:3866380; nicotinate phosphoribosyltransferase | -1.00 | 0.001 |
| SG2206; GI:85060184; GeneID:3868751; formamidopyrimidine-DNA glycosylase | -1.00 | 0.009 |
| SG0215; GI:85058193; GeneID:3868086; hypothetical protein | -1.00 | 0.004 |
| SG2036; GI:85060014; GeneID:3866747; hypothetical protein | -1.00 | 0.003 |
| SG0351; GI:85058329; GeneID:3868130; hypothetical protein | -1.00 | 0.061 |
| GI:125470019; GI:125470019; gp03; possible portal protein; similar to HkbQ of HK620 | -1.00 | 0.021 |
| SG2105; GI:85060083; GeneID:3866246; hypothetical protein | -1.00 | 0.012 |
| SG0160; GI:85058138; GeneID:3868487; hypothetical protein | -1.00 | 0.006 |
| SG1020; GI:85058998; GeneID:3867206; hypothetical protein | -1.00 | 0.010 |
| SG0805; GI:85058783; GeneID:3868014; glutamate-aspartate ABC transporter ATP-binding | -1.00 | 0.018 |
| SG1384; GI:85059362; GeneID:3867259; hypothetical protein | -1.00 | 0.012 |
| SG2418; GI:85060396; GeneID:3868417; putative phosphate ABC transporter periplasmic | -1.00 | 0.022 |
| SG1494; GI:85059472; GeneID:3868181; hypothetical protein | -1.00 | 0.028 |
| SG1512; GI:85059490; GeneID:3868238; peptide ABC transporter ATP-binding component | -1.00 | 0.017 |
| SG2310; GI:85060288; GeneID:3866316; hypothetical protein | -1.00 | 0.010 |
| SG2275; GI:85060253; GeneID:3866300; 50S ribosomal protein L2 | -1.00 | 0.004 |
| SG0597; GI:85058575; GeneID:3866665; aminoacyl-histidine dipeptidase | -1.00 | 0.025 |
| SG1817; GI:85059795; GeneID:3866644; hypothetical protein | -1.00 | 0.013 |
| SG1535; GI:85059513; GeneID:3867234; putative transport protein | -1.00 | 0.043 |
| GI:125470021; GI:125470021; gp05; possible capsid protein; similar to HkbS of HK620 | -1.00 | 0.031 |
| SG1429; GI:85059407; GeneID:3866932; phosphoenolpyruvate synthase | -1.00 | 0.044 |
| SG0262; GI:85058240; GeneID:3867902; hypothetical protein | -1.00 | 0.030 |
| SG0054; GI:85058032; GeneID:3867439; flagellar hook-basal body complex protein FliE | -1.00 | 0.036 |
| SG1578; GI:85059556; GeneID:3866691; outer membrane protein | -1.00 | 0.037 |
| SG0771; GI:85058749; GeneID:3868038; suppressor for copper-sensitivity D | -1.00 | 0.023 |
| GI:125470029; GI:125470029; gp13; possible tail spike protein; head binding domain | -1.00 | 0.041 |
| SG0402; GI:85058380; GeneID:3867571; right origin-binding protein | -1.00 | 0.032 |
| SG0519; GI:85058497; GeneID:3868220; putative alkaline phosphatase isozyme conversion | -1.00 | 0.024 |
| SG2023; GI:85060001; GeneID:3868802; hypothetical protein | -1.00 | 0.028 |
| SG1157; GI:85059135; GeneID:3866227; hypothetical protein | -1.00 | 0.029 |
| SG2035; GI:85060013; GeneID:3866746; adenine glycosylase | -1.00 | 0.030 |
| SG0802; GI:85058780; GeneID:3868485; rare lipoprotein B precursor | -1.00 | 0.031 |
| SG0393; GI:85058371; GeneID:3867640; hypothetical protein | -1.00 | 0.061 |
| GI:125470033; GI:125470033; gp17; hypothetical protein | -1.00 | 0.042 |
| SG0817; GI:85058795; GeneID:3868026; hypothetical protein | -1.00 | 0.049 |
| SG1895; GI:85059873; GeneID:3867768; hypothetical protein | -1.00 | 0.046 |
| SGP2_0006; GI:85060472; GeneID:3866607; RNA one modulator protein | -1.00 | 0.057 |
| SG1932; GI:85059910; GeneID:3867844; (3R)-hydroxymyristoyl-(acyl-carrier-protein) | -1.00 | 0.034 |
| SG1955; GI:85059933; GeneID:3866213; hypothetical protein | -1.00 | 0.028 |
| SG0650; GI:85058628; GeneID:3867996; hypothetical protein | -1.00 | 0.053 |
| SG1956; GI:85059934; GeneID:3866214; transcriptional regulator | -1.00 | 0.038 |
| SGP1_0006; GI:85060417; GeneID:3866545; hypothetical protein | -1.00 | 0.023 |
| SG2110; GI:85060088; GeneID:3866251; DNA polymerase III chi subunit | -1.00 | 0.037 |
| SG2039; GI:85060017; GeneID:3866750; hypothetical protein | -1.00 | 0.001 |
| SGP2_0015; GI:85060481; GeneID:3866593; hypothetical protein | -1.00 | 0.058 |
| SG0619; GI:85058597; GeneID:3867593; hypothetical protein | -1.00 | 0.026 |
| SG2059; GI:85060037; GeneID:3866511; putative flagellar hook formation protein FlgD | -1.00 | 0.042 |
| SG2384; GI:85060362; GeneID:3867355; putative lipopolysaccharide biosynthesis | -1.00 | 0.036 |
| SG0316; GI:85058294; GeneID:3867388; hypothetical protein | -1.00 | 0.036 |
| SG1177; GI:85059155; GeneID:3868548; hypothetical protein | -1.00 | 0.083 |
| SG1264; GI:85059242; GeneID:3867871; hypothetical protein | -1.00 | 0.039 |
| SG2242; GI:85060220; GeneID:3866719; shikimate 5-dehydrogenase | -1.00 | 0.056 |
| SG0799; GI:85058777; GeneID:3868482; hypothetical protein | -1.00 | 0.040 |
| SG2132; GI:85060110; GeneID:3867293; replicative DNA helicase | -1.00 | 0.041 |
| SGP2_0016; GI:85060482; GeneID:3866594; hypothetical protein | -1.00 | 0.052 |
| SG2146; GI:85060124; GeneID:3868255; glucose-6-phosphate isomerase Pgi | -1.00 | 0.033 |
| GI:125470065; GI:125470065; gp53; possible lysozyme; similar to gp31 of pSOG3 and to | -1.00 | 0.063 |
| SG1753; GI:85059731; GeneID:3866912; putative lactose transport protein | -1.00 | 0.026 |
| SG2255; GI:85060233; GeneID:3866732; 30S ribosomal protein S11 | -1.00 | 0.036 |
| GI:125470063; GI:125470063; gp51; hypothetical protein | -1.00 | 0.018 |
| SG0032; GI:85058010; GeneID:3867456; flagellar basal-body rod modification protein | -1.01 | 0.046 |
| SG1551; GI:85059529; GeneID:3867191; hypothetical protein | -1.01 | 0.066 |
| SG1993; GI:85059971; GeneID:3867074; phage integrase | -1.01 | 0.061 |
| SG0773; GI:85058751; GeneID:3868040; transposase | -1.01 | 0.039 |
| GI:125470028; GI:125470028; gp12; possible DNA injection protein; similar to HkcA of | -1.01 | 0.081 |
| SG0109; GI:85058087; GeneID:3867982; ubiquinone/menaquinone biosynthesis | -1.01 | 0.063 |
| SG1838; GI:85059816; GeneID:3868631; putative mannonate dehydratase | -1.01 | 0.077 |
| SG1749; GI:85059727; GeneID:3866494; inosine-5'-monophosphate dehydrogenase | -1.01 | 0.044 |
| SG2072; GI:85060050; GeneID:3868103; hypothetical protein | -1.01 | 0.058 |
| SG1363; GI:85059341; GeneID:3867335; exodeoxyribonuclease III | -1.01 | 0.055 |
| SG0210; GI:85058188; GeneID:3868081; hypothetical protein | -1.01 | 0.063 |
| SG1207; GI:85059185; GeneID:3866846; hypothetical protein | -1.01 | 0.053 |
| SG2005; GI:85059983; GeneID:3867086; proline aminopeptidase II | -1.01 | 0.065 |
| SG0423; GI:85058401; GeneID:3867534; hypothetical protein | -1.01 | 0.070 |
| SG0520; GI:85058498; GeneID:3868221; siroheme synthase | -1.01 | 0.089 |
| SG0532; GI:85058510; GeneID:3868648; hypothetical protein | -1.01 | 0.050 |
| GI:125470026; GI:125470026; gp10; possible DNA injection protein; similar to gp07 of | -1.01 | 0.073 |
| SG1224; GI:85059202; GeneID:3868677; hypothetical protein | -1.01 | 0.081 |
| SG1377; GI:85059355; GeneID:3867252; oligopeptide ABC transporter ATP-binding | -1.01 | 0.072 |
| SG2257; GI:85060235; GeneID:3866734; 50S ribosomal protein L36 | -1.01 | 0.048 |
| SG0891; GI:85058869; GeneID:3868339; putative cation transport protein | -1.01 | 0.063 |
| SG2085; GI:85060063; GeneID:3866460; type III secretion apparatus SpaO | -1.01 | 0.066 |
| SG2119; GI:85060097; GeneID:3866260; putative glucuronyl hydrolase | -1.01 | 0.096 |
| SG2062; GI:85060040; GeneID:3866514; putative flagella synthesis protein FlgN | -1.01 | 0.083 |
| SG0929; GI:85058907; GeneID:3868377; phage integrase | -1.01 | 0.072 |
| SG2352; GI:85060330; GeneID:3867180; phage assembly protein | -1.01 | 0.062 |
| SG1945; GI:85059923; GeneID:3867857; protein-PII uridylyltransferase | -1.01 | 0.067 |
| SG0123; GI:85058101; GeneID:3867957; hypothetical protein | -1.01 | 0.080 |
| SG0529; GI:85058507; GeneID:3868230; protein-L-isoaspartate O-methyltransferase | -1.01 | 0.089 |
| GI:125470027; GI:125470027; gp11; possible DNA injection protein; similar to HkbZ of | -1.01 | 0.108 |
| SGP1_0009; GI:85060420; GeneID:3866548; hypothetical protein | -1.01 | 0.101 |
| SG1014; GI:85058992; GeneID:3866378; hypothetical protein | -1.01 | 0.057 |
| SG0963; GI:85058941; GeneID:3867090; galactoside ABC transporter periplasmic | -1.01 | 0.076 |
| SG0996; GI:85058974; GeneID:3866889; tetraacyldisaccharide 4'-kinase | -1.01 | 0.066 |
| SG1877; GI:85059855; GeneID:3866351; glutamyl-tRNA reductase | -1.01 | 0.073 |
| SG1091; GI:85059069; GeneID:3868168; hypothetical protein | -1.01 | 0.109 |
| SG0812; GI:85058790; GeneID:3868021; putative 2-octaprenyl-3-methyl-6-methoxy-1,4- | -1.01 | 0.098 |
| SG1913; GI:85059891; GeneID:3867646; putative PTS system IIBC component | -1.01 | 0.081 |
| SG0913; GI:85058891; GeneID:3868361; putative ATP-dependent RNA helicase | -1.01 | 0.087 |
| SG1238; GI:85059216; GeneID:3866761; hypothetical protein | -1.01 | 0.066 |
| SG0860; GI:85058838; GeneID:3867818; glutaminyl-tRNA synthase | -1.01 | 0.081 |
| SG1239; GI:85059217; GeneID:3866762; colanic acid capsular biosynthesis activation | -1.01 | 0.085 |
| SG0830; GI:85058808; GeneID:3868772; hypothetical protein | -1.01 | 0.121 |
| SG1579; GI:85059557; GeneID:3866692; putative two-component sensor protein | -1.01 | 0.067 |
| SG1415; GI:85059393; GeneID:3866976; L-ribulose-5-phosphate 4-epimerase | -1.01 | 0.080 |
| SG1351; GI:85059329; GeneID:3867323; hypothetical protein | -1.01 | 0.081 |
| SG1041; GI:85059019; GeneID:3866953; hypothetical protein | -1.01 | 0.062 |
| SG1620; GI:85059598; GeneID:3867798; putative semialdehyde dehydrogenase | -1.01 | 0.096 |
| SG1779; GI:85059757; GeneID:3866523; hypothetical protein | -1.01 | 0.097 |
| SG0736; GI:85058714; GeneID:3867713; hypothetical protein | -1.01 | 0.131 |
| SG0489; GI:85058467; GeneID:3868703; 7,8-dihydro-6-hydroxymethylpterin- | -1.01 | 0.090 |
| SG0514; GI:85058492; GeneID:3868215; hypothetical protein | -1.01 | 0.090 |
| SG0275; GI:85058253; GeneID:3867915; DNA topoisomerase IV subunit A | -1.01 | 0.095 |
| SG1971; GI:85059949; GeneID:3866779; hypothetical protein | -1.01 | 0.080 |
| SG1633; GI:85059611; GeneID:3867156; putative cytochrome c-type biogenesis protein | -1.01 | 0.094 |
| SG1733; GI:85059711; GeneID:3866478; hypothetical protein | -1.01 | 0.148 |
| SG1759; GI:85059737; GeneID:3866918; histidyl-tRNA synthase | -1.01 | 0.097 |
| SG2353; GI:85060331; GeneID:3867181; hypothetical protein | -1.01 | 0.115 |
| SGP1_0032; GI:85060443; GeneID:3866571; hypothetical protein | -1.01 | 0.086 |
| SG0733; GI:85058711; GeneID:3867710; hypothetical protein | -1.01 | 0.115 |
| SG0559; GI:85058537; GeneID:3867611; type III secretion apparatus | -1.01 | 0.132 |
| SG0117; GI:85058095; GeneID:3867990; flavin reductase | -1.01 | 0.100 |
| GI:125470067; GI:125470067; gp55; integrase-like protein; similar to gp33 of pSOG3 | -1.01 | 0.139 |
| SG2279; GI:85060257; GeneID:3866421; 30S ribosomal protein S10 | -1.01 | 0.066 |
| SG1528; GI:85059506; GeneID:3867227; hypothetical protein | -1.01 | 0.109 |
| SG0797; GI:85058775; GeneID:3868480; penicillin-binding protein 2 | -1.01 | 0.102 |
| SG0678; GI:85058656; GeneID:3868145; ABC transporter ATP-binding component | -1.01 | 0.108 |
| SG0788; GI:85058766; GeneID:3868471; putative phage tail protein | -1.01 | 0.098 |
| SG1922; GI:85059900; GeneID:3867655; hypothetical protein | -1.01 | 0.114 |
| SG0072; GI:85058050; GeneID:3867496; hypothetical protein | -1.01 | 0.045 |
| SG0833; GI:85058811; GeneID:3868775; hypothetical protein | -1.01 | 0.160 |
| SG1471; GI:85059449; GeneID:3866896; putative guanine deaminase | -1.01 | 0.114 |
| SG1564; GI:85059542; GeneID:3866677; phage baseplate assembly protein | -1.01 | 0.169 |
| SG0853; GI:85058831; GeneID:3867811; putative phage baseplate protein | -1.01 | 0.151 |
| SG1912; GI:85059890; GeneID:3867645; putative N-acetylmannosamine-6-phosphate | -1.01 | 0.117 |
| SG1163; GI:85059141; GeneID:3866233; hypothetical protein | -1.01 | 0.108 |
| SG0510; GI:85058488; GeneID:3868289; GTP pyrophosphokinase | -1.01 | 0.113 |
| SG1127; GI:85059105; GeneID:3867555; histidinol-phosphatase and | -1.01 | 0.106 |
| SG0758; GI:85058736; GeneID:3867735; hypothetical protein | -1.01 | 0.119 |
| SG1229; GI:85059207; GeneID:3868682; phage antitermination protein | -1.01 | 0.125 |
| SG0966; GI:85058944; GeneID:3867093; vancomycin resistance protein | -1.01 | 0.138 |
| SG2021; GI:85059999; GeneID:3868800; hypothetical protein | -1.01 | 0.119 |
| SG0092; GI:85058070; GeneID:3867752; tetrahydropteroyltriglutamate methyltransferase | -1.01 | 0.141 |
| SG0601; GI:85058579; GeneID:3866669; gamma-glutamyl phosphate reductase | -1.01 | 0.122 |
| SG0791; GI:85058769; GeneID:3868474; putative phage DNA adenine methylase | -1.01 | 0.129 |
| SG1818; GI:85059796; GeneID:3866645; hypothetical protein | -1.01 | 0.142 |
| SG0026; GI:85058004; GeneID:3867450; flagellar protein FlhA | -1.01 | 0.130 |
| SG1182; GI:85059160; GeneID:3868553; hypothetical protein | -1.01 | 0.103 |
| SG0180; GI:85058158; GeneID:3866619; L-fuculokinase | -1.01 | 0.151 |
| SG1423; GI:85059401; GeneID:3866984; phenylalanyl-tRNA synthase beta subunit | -1.01 | 0.132 |
| SG0118; GI:85058096; GeneID:3867991; proline dipeptidase | -1.01 | 0.140 |
| SG1181; GI:85059159; GeneID:3868552; hypothetical protein | -1.01 | 0.121 |
| SG1577; GI:85059555; GeneID:3866690; hypothetical protein | -1.01 | 0.140 |
| SG2109; GI:85060087; GeneID:3866250; cytosol aminopeptidase | -1.01 | 0.116 |
| SG0647; GI:85058625; GeneID:3868466; preprotein translocase YajC subunit | -1.01 | 0.116 |
| SG1663; GI:85059641; GeneID:3868527; hypothetical protein | -1.01 | 0.145 |
| SG0946; GI:85058924; GeneID:3868433; hypothetical protein | -1.01 | 0.137 |
| SG1153; GI:85059131; GeneID:3867144; hypothetical protein | -1.01 | 0.127 |
| SG0432; GI:85058410; GeneID:3867543; thiamine ABC transporter permease component | -1.01 | 0.128 |
| SG0091; GI:85058069; GeneID:3867751; lysR-family transcriptional regulatory protein | -1.01 | 0.111 |
| SG0206; GI:85058184; GeneID:3868077; hypothetical protein | -1.01 | 0.148 |
| SG0368; GI:85058346; GeneID:3867480; transcription elongation factor | -1.01 | 0.145 |
| SG0120; GI:85058098; GeneID:3867993; potassium transport protein TrkH | -1.01 | 0.122 |
| SG0134; GI:85058112; GeneID:3867968; RNA polymerase beta subunit | -1.01 | 0.112 |
| SG1503; GI:85059481; GeneID:3868190; phage shock protein operon transcriptional | -1.02 | 0.184 |
| SG0633; GI:85058611; GeneID:3868452; putative efflux transport protein | -1.02 | 0.136 |
| SG0046; GI:85058024; GeneID:3867431; flagellar motor switch protein FliM | -1.02 | 0.145 |
| SG1390; GI:85059368; GeneID:3867030; hypothetical protein | -1.02 | 0.160 |
| SGP2_0003; GI:85060469; GeneID:3866604; DNA-binding protein | -1.02 | 0.154 |
| SG2368; GI:85060346; GeneID:3866825; uroporphyrinogen III methylase | -1.02 | 0.131 |
| SG2382; GI:85060360; GeneID:3867353; UDP-N-acetyl-D-mannosaminuronic acid | -1.02 | 0.157 |
| SG0861; GI:85058839; GeneID:3867819; ferric uptake regulation protein | -1.02 | 0.145 |
| SG0042; GI:85058020; GeneID:3867466; flagellar protein FliQ | -1.02 | 0.169 |
| SG2129; GI:85060107; GeneID:3867290; excinuclease ABC subunit A | -1.02 | 0.162 |
| SG0066; GI:85058044; GeneID:3867490; hypothetical protein | -1.02 | 0.153 |
| SGP2_0005; GI:85060471; GeneID:3866606; hypothetical protein | -1.02 | 0.147 |
| SG0450; GI:85058428; GeneID:3867425; UDP-N-acetylmuramate-alanine ligase | -1.02 | 0.133 |
| SG2001; GI:85059979; GeneID:3867082; glycine cleavage system H protein | -1.02 | 0.138 |
| SG2420; GI:85060398; GeneID:3868419; putative phosphate ABC transporter ATP-binding | -1.02 | 0.178 |
| SG0189; GI:85058167; GeneID:3866628; hypothetical protein | -1.02 | 0.129 |
| GI:125470023; GI:125470023; gp07; possible DNA stabilization protein; similar to gp10 | -1.02 | 0.211 |
| SG1095; GI:85059073; GeneID:3868172; arginine ABC transporter periplasmic component | -1.02 | 0.135 |
| SG2330; GI:85060308; GeneID:3867315; aspartate semialdehyde dehydrogenase Asd | -1.02 | 0.145 |
| SG2032; GI:85060010; GeneID:3866743; putative coproporphyrinogen III oxidase | -1.02 | 0.127 |
| SG1533; GI:85059511; GeneID:3867232; putative transposase | -1.02 | 0.152 |
| SG0053; GI:85058031; GeneID:3867438; flagellar basal-body M-ring protein FliF | -1.02 | 0.187 |
| SG0544; GI:85058522; GeneID:3868660; hypothetical protein | -1.02 | 0.145 |
| SG1587; GI:85059565; GeneID:3867882; hypothetical protein | -1.02 | 0.212 |
| GI:125470024; GI:125470024; gp08; possible DNA stabilization protein; similar to gp26 | -1.02 | 0.207 |
| SG2363; GI:85060341; GeneID:3866820; hypothetical protein | -1.02 | 0.187 |
| SG0437; GI:85058415; GeneID:3867412; 2-isopropylmalate synthase | -1.02 | 0.133 |
| SG1545; GI:85059523; GeneID:3867185; hypothetical protein | -1.02 | 0.176 |
| SG1447; GI:85059425; GeneID:3867264; pyridoxamine 5'-phosphate oxidase | -1.02 | 0.167 |
| SG0157; GI:85058135; GeneID:3868523; rod shape-determining protein MreC | -1.02 | 0.167 |
| SG0456; GI:85058434; GeneID:3868196; hypothetical protein | -1.02 | 0.170 |
| SG0257; GI:85058235; GeneID:3868309; bacitracin resistance protein | -1.02 | 0.152 |
| SG0543; GI:85058521; GeneID:3868659; hypothetical protein | -1.02 | 0.186 |
| SG1367; GI:85059345; GeneID:3867339; UTP-glucose-1-phosphate uridylyltransferase | -1.02 | 0.124 |
| SG0089; GI:85058067; GeneID:3867749; glycerol-3-phosphate ABC transporter permease | -1.02 | 0.182 |
| SG0582; GI:85058560; GeneID:3867696; putative lipoprotein | -1.02 | 0.162 |
| SG0594; GI:85058572; GeneID:3866662; putative amidohydrolase | -1.02 | 0.220 |
| SG0138; GI:85058116; GeneID:3867972; uroporphyrinogen decarboxylase | -1.02 | 0.170 |
| SG0212; GI:85058190; GeneID:3868083; serine protease | -1.02 | 0.170 |
| SG2126; GI:85060104; GeneID:3867287; putative dehydrogenase | -1.02 | 0.191 |
| SG1203; GI:85059181; GeneID:3866842; putative phage tail protein | -1.02 | 0.202 |
| SG1502; GI:85059480; GeneID:3868189; putative phage shock protein A | -1.02 | 0.141 |
| SG0034; GI:85058012; GeneID:3867458; flagellar basal-body rod protein FlgF | -1.02 | 0.224 |
| SG1454; GI:85059432; GeneID:3867271; putative iron-sulfur binding protein | -1.02 | 0.170 |
| SG0990; GI:85058968; GeneID:3866883; phosphoserine aminotransferase | -1.02 | 0.195 |
| SG0779; GI:85058757; GeneID:3868046; hypothetical protein | -1.02 | 0.227 |
| SG0560; GI:85058538; GeneID:3867612; putative type III secretion apparatus | -1.02 | 0.237 |
| SG0321; GI:85058299; GeneID:3867393; hypothetical protein | -1.02 | 0.229 |
| SGP2_0002; GI:85060468; GeneID:3866603; single stranded DNA-binding protein | -1.02 | 0.186 |
| SG0819; GI:85058797; GeneID:3868028; hypothetical protein | -1.02 | 0.242 |
| SG1481; GI:85059459; GeneID:3866906; transposase | -1.02 | 0.156 |
| SG1399; GI:85059377; GeneID:3867039; anthranilate isomerase TrpCF | -1.02 | 0.154 |
| SG1957; GI:85059935; GeneID:3866215; hypothetical protein | -1.02 | 0.152 |
| SG2159; GI:85060137; GeneID:3868268; argininosuccinate lyase | -1.02 | 0.180 |
| SG2428; GI:85060406; GeneID:3866324; thiophene and furan oxidation protein | -1.02 | 0.177 |
| SG0303; GI:85058281; GeneID:3868571; putative aspartate ammonia-lyase | -1.02 | 0.186 |
| SG2145; GI:85060123; GeneID:3868254; hypothetical protein | -1.02 | 0.226 |
| SG0769; GI:85058747; GeneID:3868036; suppressor for copper-sensitivity B | -1.02 | 0.180 |
| GI:125470039; GI:125470039; gp24; hypothetical protein; similar to gp22 of pSG3 | -1.02 | 0.244 |
| SG1960; GI:85059938; GeneID:3866218; hypothetical protein | -1.02 | 0.135 |
| SG0772; GI:85058750; GeneID:3868039; putative transposase | -1.02 | 0.161 |
| SG2364; GI:85060342; GeneID:3866821; hypothetical protein | -1.02 | 0.158 |
| SG1046; GI:85059024; GeneID:3866958; glycosyltransferase | -1.02 | 0.184 |
| SG0936; GI:85058914; GeneID:3868384; hypothetical protein | -1.02 | 0.169 |
| SG1825; GI:85059803; GeneID:3866652; putative phage replication protein | -1.02 | 0.182 |
| SG0836; GI:85058814; GeneID:3868778; hypothetical protein | -1.02 | 0.293 |
| SG0401; GI:85058379; GeneID:3867570; tryptophan operon repressor | -1.02 | 0.189 |
| SG1642; GI:85059620; GeneID:3867820; putative phage tail assembly protein | -1.02 | 0.180 |
| SG1047; GI:85059025; GeneID:3866959; lipid A biosynthesis lauroyl acyltransferase | -1.02 | 0.138 |
| SG1315; GI:85059293; GeneID:3867510; hypothetical protein | -1.02 | 0.187 |
| SG2155; GI:85060133; GeneID:3868264; hypothetical protein | -1.02 | 0.201 |
| SG0280; GI:85058258; GeneID:3868587; cystathionine beta-lyase | -1.02 | 0.207 |
| SG0651; GI:85058629; GeneID:3867997; riboflavin biosynthesis protein RibD | -1.02 | 0.182 |
| SG1304; GI:85059282; GeneID:3867119; putative type III secretion apparatus | -1.02 | 0.191 |
| SG1419; GI:85059397; GeneID:3866980; threonyl-tRNA synthase | -1.02 | 0.172 |
| SG1133; GI:85059111; GeneID:3867561; hypothetical protein | -1.02 | 0.219 |
| SG1671; GI:85059649; GeneID:3868535; phage lysozyme lysis protein | -1.02 | 0.205 |
| SG2052; GI:85060030; GeneID:3866504; flagellar protein FliL | -1.02 | 0.231 |
| SG1090; GI:85059068; GeneID:3868167; putative phage tail assembly protein | -1.02 | 0.182 |
| SG1952; GI:85059930; GeneID:3867346; exodeoxyribonuclease IX | -1.02 | 0.159 |
| SG0479; GI:85058457; GeneID:3868693; spermidine synthase | -1.02 | 0.213 |
| SG0606; GI:85058584; GeneID:3866674; putative transcriptional regulator | -1.02 | 0.180 |
| SG0246; GI:85058224; GeneID:3868298; hypothetical protein | -1.02 | 0.274 |
| SG1412; GI:85059390; GeneID:3866973; hypothetical protein | -1.02 | 0.203 |
| SG0256; GI:85058234; GeneID:3868308; dihydroneopterin aldolase | -1.02 | 0.213 |
| SG2328; GI:85060306; GeneID:3867313; glycerol-3-phosphate regulon protein GlpE | -1.02 | 0.238 |
| SG0116; GI:85058094; GeneID:3867989; hypothetical protein | -1.02 | 0.209 |
| SG1936; GI:85059914; GeneID:3867848; putative metalloprotease | -1.02 | 0.158 |
| SG1784; GI:85059762; GeneID:3866528; pyridoxal phosphate biosynthetic protein | -1.02 | 0.230 |
| SG0849; GI:85058827; GeneID:3867807; hypothetical protein | -1.02 | 0.311 |
| SG1408; GI:85059386; GeneID:3866969; DNA topoisomerase I | -1.02 | 0.203 |
| SG1459; GI:85059437; GeneID:3867276; putative ABC transporter ATP-binding component | -1.02 | 0.203 |
| SG1007; GI:85058985; GeneID:3866371; outer membrane protein F | -1.02 | 0.156 |
| SG0950; GI:85058928; GeneID:3868437; putative ABC transporter permease component | -1.02 | 0.203 |
| SG1221; GI:85059199; GeneID:3868674; putative phage integrase | -1.02 | 0.275 |
| SG2071; GI:85060049; GeneID:3868102; hypothetical protein | -1.02 | 0.246 |
| SG0556; GI:85058534; GeneID:3867608; putative type III secretion apparatus | -1.02 | 0.279 |
| SG2016; GI:85059994; GeneID:3868795; hypothetical protein | -1.02 | 0.290 |
| SG2265; GI:85060243; GeneID:3866290; 30S ribosomal protein S14 | -1.02 | 0.146 |
| SG2426; GI:85060404; GeneID:3868425; putative PTS system IIB component | -1.02 | 0.283 |
| SG0037; GI:85058015; GeneID:3867461; flagellar P-ring protein precursor FlgI | -1.02 | 0.268 |
| SG0475; GI:85058453; GeneID:3868689; hypothetical protein | -1.02 | 0.242 |
| SG2219; GI:85060197; GeneID:3868764; polyketide synthase | -1.02 | 0.247 |
| SG1760; GI:85059738; GeneID:3866919; 4-hydroxy-3-methylbut-2-en-1-yl diphosphate | -1.02 | 0.208 |
| SG1250; GI:85059228; GeneID:3866773; putative aminotransferase | -1.02 | 0.224 |
| SG2174; GI:85060152; GeneID:3867948; ferredoxin-NADP reductase | -1.02 | 0.221 |
| SG0834; GI:85058812; GeneID:3868776; hypothetical protein | -1.02 | 0.344 |
| SG1855; GI:85059833; GeneID:3867053; putative transcriptional regulator | -1.02 | 0.276 |
| SG0194; GI:85058172; GeneID:3866633; hypothetical protein | -1.02 | 0.318 |
| SG2102; GI:85060080; GeneID:3866477; hypothetical protein | -1.02 | 0.290 |
| SG0329; GI:85058307; GeneID:3867401; hypothetical protein | -1.02 | 0.247 |
| SG1318; GI:85059296; GeneID:3867513; putative transcriptional regulator | -1.02 | 0.221 |
| SG1313; GI:85059291; GeneID:3867508; hypothetical protein | -1.02 | 0.197 |
| SG0562; GI:85058540; GeneID:3867614; type III secretion apparatus InvE | -1.03 | 0.200 |
| SG1004; GI:85058982; GeneID:3866368; hypothetical protein | -1.03 | 0.199 |
| SG1080; GI:85059058; GeneID:3868157; hypothetical protein | -1.03 | 0.230 |
| SG0208; GI:85058186; GeneID:3868079; hypothetical protein | -1.03 | 0.250 |
| SG2247; GI:85060225; GeneID:3866724; methionyl-tRNA formyltransferase | -1.03 | 0.239 |
| SG0831; GI:85058809; GeneID:3868773; putative lipoprotein | -1.03 | 0.372 |
| SG1438; GI:85059416; GeneID:3866941; pyruvate kinase I | -1.03 | 0.190 |
| SG2225; GI:85060203; GeneID:3868809; hypothetical protein | -1.03 | 0.221 |
| SG2066; GI:85060044; GeneID:3868097; chemotaxis protein CheW | -1.03 | 0.237 |
| SG2248; GI:85060226; GeneID:3866725; putative rRNA methylase | -1.03 | 0.248 |
| SG2011; GI:85059989; GeneID:3868790; chromosome initiation inhibitor | -1.03 | 0.250 |
| SG0924; GI:85058902; GeneID:3868372; ribosomal protein S6 modification protein | -1.03 | 0.245 |
| SG0808; GI:85058786; GeneID:3868017; putative transport protein | -1.03 | 0.240 |
| SG1301; GI:85059279; GeneID:3867116; putative type III secretion ATP synthase | -1.03 | 0.247 |
| SG0683; GI:85058661; GeneID:3868150; 50S ribosomal protein L36 | -1.03 | 0.236 |
| SG0232; GI:85058210; GeneID:3868064; hypothetical protein | -1.03 | 0.333 |
| SG1078; GI:85059056; GeneID:3868155; lipoprotein releasing system transmembrane | -1.03 | 0.245 |
| SG2060; GI:85060038; GeneID:3866512; flagellar basal-body rod protein FlgB | -1.03 | 0.175 |
| SG1964; GI:85059942; GeneID:3866222; hypothetical protein | -1.03 | 0.266 |
| SG1998; GI:85059976; GeneID:3867079; putative hemolysin | -1.03 | 0.228 |
| SG2177; GI:85060155; GeneID:3867951; sulfate ABC transporter periplasmic component | -1.03 | 0.203 |
| SG1905; GI:85059883; GeneID:3867778; glycine betaine/L-proline ABC transporter | -1.03 | 0.333 |
| SG0675; GI:85058653; GeneID:3868142; peptidyl-prolyl cis-trans isomerase D | -1.03 | 0.264 |
| SG0030; GI:85058008; GeneID:3867454; flagellar basal-body rod protein FlgB | -1.03 | 0.279 |
| SG0509; GI:85058487; GeneID:3868288; putative RNA methyltransferase | -1.03 | 0.266 |
| SG0495; GI:85058473; GeneID:3868274; putative tetR-family transcriptional regulator | -1.03 | 0.256 |
| SG2402; GI:85060380; GeneID:3868401; low-affinity potassium transport protein | -1.03 | 0.254 |
| SG0548; GI:85058526; GeneID:3868664; tRNA (guanine-N1)-methyltransferase | -1.03 | 0.213 |
| SG1242; GI:85059220; GeneID:3866765; hypothetical protein | -1.03 | 0.275 |
| SG0965; GI:85058943; GeneID:3867092; galactoside ABC transporter permease component | -1.03 | 0.228 |
| SG2181; GI:85060159; GeneID:3866989; two-component sensor kinase | -1.03 | 0.270 |
| SG1893; GI:85059871; GeneID:3867766; hypothetical protein | -1.03 | 0.295 |
| SG2326; GI:85060304; GeneID:3867311; glycerol-3-phosphate regulon repressor GlpR | -1.03 | 0.243 |
| SG1382; GI:85059360; GeneID:3867257; hypothetical protein | -1.03 | 0.219 |
| SG0367; GI:85058345; GeneID:3867479; hypothetical protein | -1.03 | 0.248 |
| SG0341; GI:85058319; GeneID:3868120; hypothetical protein | -1.03 | 0.240 |
| SG1542; GI:85059520; GeneID:3867241; putative nitroreductase | -1.03 | 0.305 |
| SG1748; GI:85059726; GeneID:3866493; GMP synthase | -1.03 | 0.187 |
| SG0433; GI:85058411; GeneID:3867408; thiamine ABC transporter periplasmic component | -1.03 | 0.289 |
| SG1800; GI:85059778; GeneID:3866803; DNA repair protein RecN | -1.03 | 0.228 |
| SG2006; GI:85059984; GeneID:3868785; hypothetical protein | -1.03 | 0.267 |
| SG0857; GI:85058835; GeneID:3867815; phage tail fiber protein | -1.03 | 0.384 |
| SG1342; GI:85059320; GeneID:3866413; alanine racemase | -1.03 | 0.269 |
| SG2423; GI:85060401; GeneID:3868422; hypothetical protein | -1.03 | 0.269 |
| SG0781; GI:85058759; GeneID:3868048; hypothetical protein | -1.03 | 0.316 |
| SG0886; GI:85058864; GeneID:3868334; colicin import protein TolB | -1.03 | 0.272 |
| SG0575; GI:85058553; GeneID:3867689; hypothetical protein | -1.03 | 0.252 |
| SG1366; GI:85059344; GeneID:3867338; putative regulator | -1.03 | 0.247 |
| SG2157; GI:85060135; GeneID:3868266; soluble pyridine nucleotide transhydrogenase | -1.03 | 0.248 |
| SGP1_0002; GI:85060413; GeneID:3866541; DNA-binding protein | -1.03 | 0.194 |
| SG2336; GI:85060314; GeneID:3867321; threonine efflux transport protein | -1.03 | 0.291 |
| SG0225; GI:85058204; GeneID:3868057; hypothetical protein | -1.03 | 0.292 |
| SG1930; GI:85059908; GeneID:3867842; lipid-A-disaccharide synthase | -1.03 | 0.271 |
| SG2362; GI:85060340; GeneID:3866819; hypothetical protein | -1.03 | 0.308 |
| SGP1_0025; GI:85060436; GeneID:3866564; hypothetical protein | -1.03 | 0.228 |
| SG1670; GI:85059648; GeneID:3868534; hypothetical protein | -1.03 | 0.415 |
| SG0025; GI:85058003; GeneID:3867449; flagellar biosynthetic protein FlhB | -1.03 | 0.270 |
| SG0332; GI:85058310; GeneID:3867404; hypothetical protein | -1.03 | 0.269 |
| SG2360; GI:85060338; GeneID:3866817; phage lysozyme lysis protein | -1.03 | 0.310 |
| SG2142; GI:85060120; GeneID:3868251; glycerol-3-phosphate acyltransferase | -1.03 | 0.306 |
| SG0400; GI:85058378; GeneID:3867569; putative ABC transporter ATP-binding component | -1.03 | 0.269 |
| SG1958; GI:85059936; GeneID:3866216; hypothetical protein | -1.03 | 0.281 |
| GI:125470040; GI:125470040; gp25; hypothetical protein; similar to gp21 of pSG3, gp2 | -1.03 | 0.214 |
| SG1929; GI:85059907; GeneID:3867841; ribonuclease HII | -1.03 | 0.297 |
| SG2020; GI:85059998; GeneID:3868799; hypothetical protein | -1.03 | 0.276 |
| SG1111; GI:85059089; GeneID:3868721; seryl-tRNA synthase | -1.03 | 0.262 |
| SG0007; GI:85057985; GeneID:3867664; putative transposase | -1.03 | 0.275 |
| SG0563; GI:85058541; GeneID:3867615; type III secretion apparatus InvA | -1.03 | 0.455 |
| SG2111; GI:85060089; GeneID:3866252; valyl-tRNA synthase | -1.03 | 0.297 |
| SG0061; GI:85058039; GeneID:3867446; dipeptide ABC transporter permease component | -1.03 | 0.207 |
| SG0451; GI:85058429; GeneID:3867426; cell division protein FtsQ | -1.03 | 0.270 |
| SG1277; GI:85059255; GeneID:3866387; putative copper resistance protein | -1.03 | 0.290 |
| SG0699; GI:85058677; GeneID:3868616; putative ABC transporter ATP-binding protein | -1.03 | 0.264 |
| SG2033; GI:85060011; GeneID:3866744; hypothetical protein | -1.03 | 0.286 |
| SG2127; GI:85060105; GeneID:3867288; transcriptional regulator | -1.03 | 0.335 |
| SG2137; GI:85060115; GeneID:3867298; putative colanic acid capsular biosynthesis | -1.03 | 0.429 |
| SG0920; GI:85058898; GeneID:3868368; putative molybdopterin biosynthesis protein | -1.03 | 0.269 |
| SG1209; GI:85059187; GeneID:3866848; hypothetical protein | -1.03 | 0.245 |
| SG1647; GI:85059625; GeneID:3867825; hypothetical protein | -1.03 | 0.337 |
| SG0310; GI:85058288; GeneID:3868578; lysyl-tRNA synthase | -1.03 | 0.292 |
| SG0511; GI:85058489; GeneID:3868212; hypothetical protein | -1.03 | 0.321 |
| SG0554; GI:85058532; GeneID:3867606; hypothetical protein | -1.03 | 0.389 |
| SG2296; GI:85060274; GeneID:3866438; putative ABC transporter ATP-binding component | -1.03 | 0.303 |
| SG0488; GI:85058466; GeneID:3868702; 3-methyl-2-oxobutanoate | -1.03 | 0.300 |
| SG1474; GI:85059452; GeneID:3866899; exochitinase | -1.03 | 0.344 |
| SG1378; GI:85059356; GeneID:3867253; cardiolipin synthase | -1.03 | 0.320 |
| SGP1_0008; GI:85060419; GeneID:3866547; TriJ | -1.03 | 0.247 |
| SG1098; GI:85059076; GeneID:3868708; putative L-allo-threonine aldolase | -1.03 | 0.317 |
| SG1608; GI:85059586; GeneID:3867786; hypothetical protein | -1.03 | 0.312 |
| SG0750; GI:85058728; GeneID:3867727; hypothetical protein | -1.03 | 0.375 |
| SG0459; GI:85058437; GeneID:3868199; hypothetical protein | -1.03 | 0.302 |
| SG1611; GI:85059589; GeneID:3867789; putative histidine ABC transporter ATP-binding | -1.03 | 0.343 |
| SG0506; GI:85058484; GeneID:3868285; putative glucarate dehydratase | -1.03 | 0.333 |
| SG2315; GI:85060293; GeneID:3866321; heat shock protein | -1.03 | 0.322 |
| SG0537; GI:85058515; GeneID:3868653; DNA recombinase A RecA | -1.03 | 0.323 |
| SG0169; GI:85058147; GeneID:3868496; hypothetical protein | -1.03 | 0.336 |
| SG1729; GI:85059707; GeneID:3866868; hypothetical protein | -1.03 | 0.265 |
| SG0943; GI:85058921; GeneID:3868430; hypothetical protein | -1.03 | 0.360 |
| SG0816; GI:85058794; GeneID:3868025; putative phage transcriptional regulator | -1.03 | 0.444 |
| SG1805; GI:85059783; GeneID:3866808; hypothetical protein | -1.03 | 0.282 |
| SG2229; GI:85060207; GeneID:3868813; putative GTP-binding elongation factor | -1.03 | 0.305 |
| SG1972; GI:85059950; GeneID:3866780; exodeoxyribonuclease V alpha subunit | -1.03 | 0.309 |
| SG0413; GI:85058391; GeneID:3867582; riboflavin biosynthesis protein | -1.03 | 0.328 |
| SG2003; GI:85059981; GeneID:3867084; putative monooxygenase | -1.03 | 0.350 |
| SG0949; GI:85058927; GeneID:3868436; putative ABC transporter permease component | -1.03 | 0.313 |
| SG1299; GI:85059277; GeneID:3867114; type III secretion apparatus | -1.03 | 0.346 |
| SG0760; GI:85058738; GeneID:3867737; hypothetical protein | -1.03 | 0.340 |
| SG1576; GI:85059554; GeneID:3866689; hypothetical protein | -1.03 | 0.350 |
| SG0992; GI:85058970; GeneID:3866885; cytidylate kinase | -1.04 | 0.316 |
| SG0713; GI:85058691; GeneID:3868733; hypothetical protein | -1.04 | 0.305 |
| SG2004; GI:85059982; GeneID:3867085; 2-octaprenyl-6-methoxyphenol hydroxylase | -1.04 | 0.334 |
| SG0142; GI:85058120; GeneID:3868508; phosphoribosylglycineamide synthase | -1.04 | 0.335 |
| SG1334; GI:85059312; GeneID:3866405; cell division topological specificity factor | -1.04 | 0.366 |
| SG1417; GI:85059395; GeneID:3866978; hypothetical protein | -1.04 | 0.459 |
| SG0158; GI:85058136; GeneID:3868524; rod shape-determining protein MreD | -1.04 | 0.342 |
| SG0607; GI:85058585; GeneID:3866675; hypothetical protein | -1.04 | 0.428 |
| SG1675; GI:85059653; GeneID:3868539; nucleoside transport protein NupC | -1.04 | 0.300 |
| SG1612; GI:85059590; GeneID:3867790; 3-octaprenyl-4-hydroxybenzoate carboxy-lyase | -1.04 | 0.298 |
| SG2202; GI:85060180; GeneID:3868747; putative lipopolysaccharide glycosyltransferase | -1.04 | 0.307 |
| SG2287; GI:85060265; GeneID:3866429; hypothetical protein | -1.04 | 0.323 |
| SG0274; GI:85058252; GeneID:3867914; hypothetical protein | -1.04 | 0.377 |
| SG0697; GI:85058675; GeneID:3868614; hypothetical protein | -1.04 | 0.306 |
| SG0087; GI:85058065; GeneID:3867747; RNA polymerase sigma-32 factor RpoH | -1.04 | 0.248 |
| SG0137; GI:85058115; GeneID:3867971; regulator of sigma-D | -1.04 | 0.356 |
| SG1123; GI:85059101; GeneID:3867551; phosphoribosyl-AMP cyclohydrolase HisIE | -1.04 | 0.319 |
| SG2321; GI:85060299; GeneID:3867306; hypothetical protein | -1.04 | 0.369 |
| SG0002; GI:85057980; GeneID:3867682; DNA polymerase III beta subunit protein DnaN | -1.04 | 0.347 |
| SG0276; GI:85058254; GeneID:3867916; 1-acyl-glycerol-3-phosphate acyltransferase | -1.04 | 0.326 |
| SG1851; GI:85059829; GeneID:3867049; hypothetical protein | -1.04 | 0.331 |
| SG0004; GI:85057982; GeneID:3867661; DNA gyrase subunit B GyrB | -1.04 | 0.311 |
| SG1002; GI:85058980; GeneID:3866366; hypothetical protein | -1.04 | 0.408 |
| SG1442; GI:85059420; GeneID:3866945; hypothetical protein | -1.04 | 0.343 |
| SG1661; GI:85059639; GeneID:3867839; hypothetical protein | -1.04 | 0.394 |
| GI:125470060; GI:125470060; gp48; possible serine protease | -1.04 | 0.545 |
| SG0035; GI:85058013; GeneID:3867459; flagellar basal-body rod protein FlgG | -1.04 | 0.451 |
| SG1394; GI:85059372; GeneID:3867034; hypothetical protein | -1.04 | 0.329 |
| SG1527; GI:85059505; GeneID:3867226; hypothetical protein | -1.04 | 0.357 |
| SG0249; GI:85058227; GeneID:3868301; hypothetical protein | -1.04 | 0.350 |
| SG0411; GI:85058389; GeneID:3867580; sodium-hydrogen antiporter | -1.04 | 0.394 |
| SG1372; GI:85059350; GeneID:3867247; aldehyde-alcohol dehydrogenase | -1.04 | 0.334 |
| SG0655; GI:85058633; GeneID:3868001; phosphatidylglycerophosphatase A | -1.04 | 0.356 |
| SG1462; GI:85059440; GeneID:3867279; fumarate hydratase class II | -1.04 | 0.282 |
| SG0273; GI:85058251; GeneID:3867913; putative dioxygenase | -1.04 | 0.366 |
| SG1385; GI:85059363; GeneID:3867260; hypothetical protein | -1.04 | 0.468 |
| SG2345; GI:85060323; GeneID:3867173; hypothetical protein | -1.04 | 0.313 |
| SG1312; GI:85059290; GeneID:3867507; amino acid ABC transporter periplasmic | -1.04 | 0.423 |
| SG0551; GI:85058529; GeneID:3867603; putative invasion protein | -1.04 | 0.320 |
| SG1472; GI:85059450; GeneID:3866897; hypothetical protein | -1.04 | 0.515 |
| SG1764; GI:85059742; GeneID:3866923; nucleoside diphosphate kinase | -1.04 | 0.357 |
| SG1368; GI:85059346; GeneID:3867340; putative UDP-glucose dehydrogenase | -1.04 | 0.350 |
| SG0718; GI:85058696; GeneID:3868738; hypothetical protein | -1.04 | 0.507 |
| SG0625; GI:85058603; GeneID:3867599; putative dehydrogenase | -1.04 | 0.478 |
| SG2425; GI:85060403; GeneID:3868424; putative PTS system IIC component | -1.04 | 0.382 |
| SG1836; GI:85059814; GeneID:3868629; hypothetical protein | -1.04 | 0.340 |
| SG2184; GI:85060162; GeneID:3866992; glycerol-3-phosphate dehydrogenase | -1.04 | 0.360 |
| SGP1_0029; GI:85060440; GeneID:3866568; hypothetical protein | -1.04 | 0.336 |
| SG1601; GI:85059579; GeneID:3867896; NADH dehydrogenase I subunit A | -1.04 | 0.382 |
| GI:125470059; GI:125470059; gp47; hypothetical protein | -1.04 | 0.557 |
| SG0663; GI:85058641; GeneID:3868009; cytochrome o ubiquinol oxidase C subunit | -1.04 | 0.331 |
| SG0599; GI:85058577; GeneID:3866667; hypothetical protein | -1.04 | 0.355 |
| SG0288; GI:85058266; GeneID:3868595; putative branched-chain amino acid ABC | -1.04 | 0.554 |
| SG2377; GI:85060355; GeneID:3867348; putative lipopolysaccharide biosynthesis | -1.04 | 0.430 |
| SG1693; GI:85059671; GeneID:3866276; hypothetical protein | -1.04 | 0.381 |
| SG0154; GI:85058132; GeneID:3868520; 3-dehydroquinate dehydratase II | -1.04 | 0.398 |
| SG1497; GI:85059475; GeneID:3868184; putative periplasmic murein peptide-binding | -1.04 | 0.447 |
| SG0662; GI:85058640; GeneID:3868008; putative transport protein | -1.04 | 0.425 |
| SG1648; GI:85059626; GeneID:3867826; hypothetical protein | -1.04 | 0.422 |
| SG0383; GI:85058361; GeneID:3867630; inducible ATP-independent RNA helicase | -1.04 | 0.335 |
| SG1063; GI:85059041; GeneID:3866703; 4-amino-4-deoxychorismate lyase | -1.04 | 0.353 |
| SG0149; GI:85058127; GeneID:3868515; hypothetical protein | -1.04 | 0.512 |
| SG1719; GI:85059697; GeneID:3866858; putative carboxypeptidase | -1.04 | 0.414 |
| SG0060; GI:85058038; GeneID:3867445; dipeptide ABC transporter periplasmic component | -1.04 | 0.386 |
| GI:125470035; GI:125470035; gp19; RecT-family protein; similar to proteins from | -1.04 | 0.554 |
| SG0851; GI:85058829; GeneID:3867809; putative phage tail fiber protein | -1.04 | 0.454 |
| SGP2_0014; GI:85060480; GeneID:3866592; hypothetical protein | -1.04 | 0.291 |
| SG1588; GI:85059566; GeneID:3867883; hypothetical protein | -1.04 | 0.391 |
| SG0616; GI:85058594; GeneID:3867590; putative transcriptional regulator | -1.04 | 0.416 |
| SG1309; GI:85059287; GeneID:3867124; hypothetical protein | -1.04 | 0.430 |
| SG1532; GI:85059510; GeneID:3867231; hypothetical protein | -1.04 | 0.344 |
| SG1547; GI:85059525; GeneID:3867187; putative fumarate reductase iron-sulfur protein | -1.04 | 0.576 |
| SG0449; GI:85058427; GeneID:3867424; UDP-N-acetylglucosamine:N-acetylmuramyl- | -1.04 | 0.375 |
| SG1629; GI:85059607; GeneID:3867152; hypothetical protein | -1.04 | 0.394 |
| GI:125470018; GI:125470018; gp02; possible TerL subunit; similar to gp02 of P22 | -1.04 | 0.670 |
| SG0768; GI:85058746; GeneID:3868035; transposase | -1.04 | 0.398 |
| SG2097; GI:85060075; GeneID:3866472; hypothetical protein | -1.04 | 0.386 |
| SG0862; GI:85058840; GeneID:3868310; flavodoxin 1 | -1.04 | 0.413 |
| SG0688; GI:85058666; GeneID:3868605; adenine phosphoribosyltransferase | -1.04 | 0.430 |
| SG0083; GI:85058061; GeneID:3867743; hypothetical protein | -1.04 | 0.425 |
| SG1831; GI:85059809; GeneID:3868624; hypothetical protein | -1.04 | 0.526 |
| SG2147; GI:85060125; GeneID:3868256; aspartokinase III LysC | -1.04 | 0.452 |
| SG0494; GI:85058472; GeneID:3868273; penicillin-binding protein 1B | -1.04 | 0.388 |
| SG2354; GI:85060332; GeneID:3867182; hypothetical protein | -1.04 | 0.418 |
| SG1260; GI:85059238; GeneID:3867867; crossover junction endodeoxyribonuclease | -1.04 | 0.413 |
| SG2416; GI:85060394; GeneID:3868415; UDP-N-acetylglucosamine pyrophosphorylase | -1.04 | 0.482 |
| SG0478; GI:85058456; GeneID:3868692; S-adenosylmethionine decarboxylase proenzyme | -1.04 | 0.370 |
| SG1001; GI:85058979; GeneID:3866365; killing factor KicB | -1.04 | 0.349 |
| SG1314; GI:85059292; GeneID:3867509; hypothetical protein | -1.04 | 0.441 |
| SG2346; GI:85060324; GeneID:3867174; diaminopimelate epimerase | -1.04 | 0.394 |
| SG0835; GI:85058813; GeneID:3868777; hypothetical protein | -1.04 | 0.607 |
| SG0125; GI:85058103; GeneID:3867959; bifunctional protein biotin operon repressor and | -1.05 | 0.499 |
| SG0063; GI:85058041; GeneID:3867487; dipeptide ABC transporter ATP-binding component | -1.05 | 0.396 |
| SG0798; GI:85058776; GeneID:3868481; hypothetical protein | -1.05 | 0.434 |
| SG2215; GI:85060193; GeneID:3868760; hypothetical protein | -1.05 | 0.421 |
| SG2118; GI:85060096; GeneID:3866259; hypothetical protein | -1.05 | 0.530 |
| SG1742; GI:85059720; GeneID:3866487; hypothetical protein | -1.05 | 0.441 |
| SG2009; GI:85059987; GeneID:3868788; D-3-phosphoglycerate dehydrogenase | -1.05 | 0.429 |
| SG1131; GI:85059109; GeneID:3867559; hypothetical protein | -1.05 | 0.478 |
| SG0362; GI:85058340; GeneID:3867474; octaprenyl-diphosphate synthase | -1.05 | 0.417 |
| SGP3_0007; GI:85060497; GeneID:3866618; hypothetical protein | -1.05 | 0.408 |
| SG1033; GI:85059011; GeneID:3867219; hypothetical protein | -1.05 | 0.413 |
| SG1672; GI:85059650; GeneID:3868536; GroEL supressor protein SugE | -1.05 | 0.345 |
| SG2233; GI:85060211; GeneID:3868817; hypothetical protein | -1.05 | 0.449 |
| SG1722; GI:85059700; GeneID:3866861; hypothetical protein | -1.05 | 0.497 |
| SG1371; GI:85059349; GeneID:3867246; putative thymidine kinase | -1.05 | 0.507 |
| SG0502; GI:85058480; GeneID:3868281; hypothetical protein | -1.05 | 0.528 |
| SG1702; GI:85059680; GeneID:3866448; PEP-protein phosphotransferase enzyme I | -1.05 | 0.430 |
| SG1124; GI:85059102; GeneID:3867552; imidazole glycerol phosphate synthase subunit | -1.05 | 0.451 |
| SG1032; GI:85059010; GeneID:3867218; methylglyoxal synthase | -1.05 | 0.438 |
| SG2334; GI:85060312; GeneID:3867319; hypothetical protein | -1.05 | 0.482 |
| GI:125470031; GI:125470031; gp15; possible YhgA-like transposase; similar to | -1.05 | 0.451 |
| SG0542; GI:85058520; GeneID:3868658; autoinducer-2 production protein | -1.05 | 0.452 |
| SG1226; GI:85059204; GeneID:3868679; putative phage anti-termination protein | -1.05 | 0.417 |
| SG0549; GI:85058527; GeneID:3868665; 50S ribosomal protein L19 | -1.05 | 0.346 |
| SG1615; GI:85059593; GeneID:3867793; putative lipoprotein | -1.05 | 0.461 |
| SG2372; GI:85060350; GeneID:3866829; transposase | -1.05 | 0.381 |
| SG1592; GI:85059570; GeneID:3867887; NADH dehydrogenase I subunit K | -1.05 | 0.442 |
| SG0824; GI:85058802; GeneID:3868766; hypothetical protein | -1.05 | 0.719 |
| SG1757; GI:85059735; GeneID:3866916; putative lipoprotein | -1.05 | 0.442 |
| SG1606; GI:85059584; GeneID:3867784; acetate kinase | -1.05 | 0.398 |
| SG2401; GI:85060379; GeneID:3868400; putative sugar ABC transporter permease | -1.05 | 0.494 |
| SG1234; GI:85059212; GeneID:3866757; hypothetical protein | -1.05 | 0.468 |
| SG0618; GI:85058596; GeneID:3867592; hypothetical protein | -1.05 | 0.508 |
| SG2106; GI:85060084; GeneID:3866247; putative transcriptional regulator | -1.05 | 0.390 |
| SG1959; GI:85059937; GeneID:3866217; hypothetical protein | -1.05 | 0.478 |
| SG2217; GI:85060195; GeneID:3868762; hypothetical protein | -1.05 | 0.485 |
| SG1021; GI:85058999; GeneID:3867207; ABC transporter ATP-binding component | -1.05 | 0.452 |
| SG0261; GI:85058239; GeneID:3867901; ADP-heptose synthase | -1.05 | 0.532 |
| GI:rep_origin; GI:rep_origin; contains four repetitions of 17 nucleotide sequence ACCGCTTTTTGGTCGTC | -1.05 | 0.594 |
| SG2282; GI:85060260; GeneID:3866424; putative transport protein | -1.05 | 0.530 |
| SG0828; GI:85058806; GeneID:3868770; putative methyltransferase | -1.05 | 0.607 |
| SG1257; GI:85059235; GeneID:3867864; aspartyl-tRNA synthase | -1.05 | 0.509 |
| SG1335; GI:85059313; GeneID:3866406; cell division inhibitor | -1.05 | 0.496 |
| SG0200; GI:85058178; GeneID:3868071; putative ABC transporter ATP-binding component | -1.05 | 0.497 |
| SG0568; GI:85058546; GeneID:3867620; type III secretion apparatus SpaO | -1.05 | 0.492 |
| SG0801; GI:85058779; GeneID:3868484; DNA polymerase III delta subunit | -1.05 | 0.531 |
| SG0698; GI:85058676; GeneID:3868615; hypothetical protein | -1.05 | 0.452 |
| SG0576; GI:85058554; GeneID:3867690; hypothetical protein | -1.05 | 0.517 |
| SG0764; GI:85058742; GeneID:3867741; hypothetical protein | -1.05 | 0.567 |
| SG1652; GI:85059630; GeneID:3867830; hypothetical protein | -1.05 | 0.520 |
| SG2008; GI:85059986; GeneID:3868787; hypothetical protein | -1.05 | 0.483 |
| SG0443; GI:85058421; GeneID:3867418; penicillin-binding protein 3 | -1.05 | 0.390 |
| SG1019; GI:85058997; GeneID:3867205; putative iron-sulfur binding protein | -1.05 | 0.506 |
| SG1176; GI:85059154; GeneID:3868547; hypothetical protein | -1.05 | 0.333 |
| SG0926; GI:85058904; GeneID:3868374; putrescine ABC transporter ATP-binding | -1.05 | 0.499 |
| SG0110; GI:85058088; GeneID:3867983; hypothetical protein | -1.05 | 0.484 |
| SG0167; GI:85058145; GeneID:3868494; hypothetical protein | -1.05 | 0.505 |
| SG1084; GI:85059062; GeneID:3868161; hypothetical protein | -1.05 | 0.470 |
| SG1773; GI:85059751; GeneID:3866517; putative gluconate 5-dehydrogenase | -1.05 | 0.518 |
| SG0381; GI:85058359; GeneID:3867628; polyribonucleotide nucleotidyltransferase | -1.05 | 0.408 |
| SG2136; GI:85060114; GeneID:3867297; conserced hypothetical protein | -1.05 | 0.564 |
| SG1457; GI:85059435; GeneID:3867274; hypothetical protein | -1.05 | 0.481 |
| SG0233; GI:85058211; GeneID:3868065; putative exu regulon transcriptional regulator | -1.05 | 0.671 |
| SG0387; GI:85058365; GeneID:3867634; hypothetical protein | -1.05 | 0.539 |
| SGP1_0036; GI:85060447; GeneID:3866575; achromobactin ABC transporter permease component | -1.05 | 0.502 |
| SG1364; GI:85059342; GeneID:3867336; formyltetrahydrofolate deformylase | -1.05 | 0.519 |
| GI:125470051; GI:125470051; gp37; possible phage-related protein; similar to gp07 of | -1.05 | 0.541 |
| SG0508; GI:85058486; GeneID:3868287; two-component regulatory protein | -1.05 | 0.588 |
| GI:125470030; GI:125470030; gp14; possible tail filament assembly protein; similar to | -1.05 | 0.719 |
| SG0311; GI:85058289; GeneID:3868579; putative periplasmic binding protein | -1.05 | 0.591 |
| SG0113; GI:85058091; GeneID:3867986; sec-independent protein translocase protein | -1.05 | 0.514 |
| SG0153; GI:85058131; GeneID:3868519; biotin carboxyl carrier protein | -1.05 | 0.563 |
| SG0995; GI:85058973; GeneID:3866888; ABC transporter ATP-binding component | -1.05 | 0.525 |
| SG1103; GI:85059081; GeneID:3868713; translation initiation factor IF-1 | -1.05 | 0.450 |
| SG0934; GI:85058912; GeneID:3868382; hypothetical protein | -1.05 | 0.406 |
| SGP1_0007; GI:85060418; GeneID:3866546; hypothetical protein | -1.05 | 0.715 |
| SG1436; GI:85059414; GeneID:3866939; hypothetical protein | -1.05 | 0.495 |
| SG0086; GI:85058064; GeneID:3867746; cell division protein | -1.05 | 0.437 |
| SG1135; GI:85059113; GeneID:3867126; putative transport protein | -1.05 | 0.515 |
| SG1005; GI:85058983; GeneID:3866369; hypothetical protein | -1.05 | 0.508 |
| SG0917; GI:85058895; GeneID:3868365; putative DNA-binding protein | -1.05 | 0.356 |
| SG0727; GI:85058705; GeneID:3867704; phage methyltransferase | -1.05 | 0.651 |
| SG1003; GI:85058981; GeneID:3866367; cell division protein | -1.05 | 0.610 |
| SG2086; GI:85060064; GeneID:3866461; putative type III secretion apparatus | -1.06 | 0.534 |
| SG1526; GI:85059504; GeneID:3867225; hypothetical protein | -1.06 | 0.548 |
| SG0229; GI:85058207; GeneID:3868061; putative transcriptional regulator | -1.06 | 0.408 |
| SG2371; GI:85060349; GeneID:3866828; transcriptional regulator | -1.06 | 0.633 |
| SG0821; GI:85058799; GeneID:3868030; hypothetical protein | -1.06 | 0.648 |
| SG0446; GI:85058424; GeneID:3867421; phospho-N-acetylmuramoyl-pentapeptide- | -1.06 | 0.486 |
| SG0579; GI:85058557; GeneID:3867693; chorismate mutase T/prephenate dehydrogenase | -1.06 | 0.525 |
| SG0889; GI:85058867; GeneID:3868337; quinolinate synthase A | -1.06 | 0.532 |
| SG2213; GI:85060191; GeneID:3868758; ribonuclease PH | -1.06 | 0.599 |
| SG1539; GI:85059517; GeneID:3867238; hemin ABC transporter permease component | -1.06 | 0.516 |
| SG0624; GI:85058602; GeneID:3867598; putative transcriptional regulator | -1.06 | 0.531 |
| SG0545; GI:85058523; GeneID:3868661; signal recognition particle protein | -1.06 | 0.555 |
| SG0049; GI:85058027; GeneID:3867434; flagellar protein FliJ | -1.06 | 0.723 |
| SG2226; GI:85060204; GeneID:3868810; ribonuclease BN | -1.06 | 0.528 |
| SG2187; GI:85060165; GeneID:3866995; hypothetical protein | -1.06 | 0.616 |
| SG0128; GI:85058106; GeneID:3867962; preprotein translocase SecE subunit | -1.06 | 0.448 |
| SG1718; GI:85059696; GeneID:3866857; succinyl-diaminopimelate desuccinylase | -1.06 | 0.626 |
| SG2050; GI:85060028; GeneID:3866502; hypothetical protein | -1.06 | 0.733 |
| SG0553; GI:85058531; GeneID:3867605; hypothetical protein | -1.06 | 0.638 |
| SG1752; GI:85059730; GeneID:3866911; putative lactose operon repressor | -1.06 | 0.534 |
| SG1483; GI:85059461; GeneID:3866908; hypothetical protein | -1.06 | 0.629 |
| SGtRNA07; GeneID:3867009; tRNA-Thr | -1.06 | 0.400 |
| SG2408; GI:85060386; GeneID:3868407; ATP synthase subunit A | -1.06 | 0.534 |
| SG1269; GI:85059247; GeneID:3867876; hex regulon repressor | -1.06 | 0.569 |
| SG0573; GI:85058551; GeneID:3867687; putative type III secretion chaperone | -1.06 | 0.653 |
| SG1761; GI:85059739; GeneID:3866920; hypothetical protein | -1.06 | 0.579 |
| SG1777; GI:85059755; GeneID:3866521; putative two-component transcriptional | -1.06 | 0.517 |
| SG2261; GI:85060239; GeneID:3866286; 30S ribosomal protein S5 | -1.06 | 0.417 |
| SGP3_0005; GI:85060495; GeneID:3866616; hypothetical protein | -1.06 | 0.600 |
| SG1477; GI:85059455; GeneID:3866902; hypothetical protein | -1.06 | 0.733 |
| SG2313; GI:85060291; GeneID:3866319; putative dehydrogenase | -1.06 | 0.599 |
| SG1840; GI:85059818; GeneID:3868633; hypothetical protein | -1.06 | 0.502 |
| SG0976; GI:85058954; GeneID:3867103; amylovoran biosynthesis protein AmsL | -1.06 | 0.662 |
| SG2055; GI:85060033; GeneID:3866507; flagellar biosynthesis protein FliO | -1.06 | 0.608 |
| SG0239; GI:85058217; GeneID:3868291; putative heptosyl transferase | -1.06 | 0.468 |
| SG1580; GI:85059558; GeneID:3866693; capsular synthesis regulator component B | -1.06 | 0.576 |
| SG2359; GI:85060337; GeneID:3866816; hypothetical protein | -1.06 | 0.583 |
| SG0640; GI:85058618; GeneID:3868459; branched-chain amino acid transport system II | -1.06 | 0.600 |
| SG0948; GI:85058926; GeneID:3868435; putative ABC transporter ATP-binding component | -1.06 | 0.572 |
| SG2204; GI:85060182; GeneID:3868749; lipopolysaccharide glycosyltransferase | -1.06 | 0.555 |
| SG1009; GI:85058987; GeneID:3866373; hypothetical protein | -1.06 | 0.630 |
| SGP3_0001; GI:85060491; GeneID:3866612; hypothetical protein | -1.06 | 0.483 |
| SG2414; GI:85060392; GeneID:3868413; ATP synthase beta subunit | -1.06 | 0.470 |
| SG1455; GI:85059433; GeneID:3867272; hypothetical protein | -1.06 | 0.539 |
| SG1450; GI:85059428; GeneID:3867267; hypothetical protein | -1.06 | 0.581 |
| SG2299; GI:85060277; GeneID:3866305; phosphoribulokinase PrkB | -1.06 | 0.529 |
| SG0227; GI:85058205; GeneID:3868059; hypothetical protein | -1.06 | 0.454 |
| SG1022; GI:85059000; GeneID:3867208; paraquat-inducible protein A | -1.06 | 0.668 |
| SG0605; GI:85058583; GeneID:3866673; transcriptional regulator | -1.06 | 0.537 |
| SG1082; GI:85059060; GeneID:3868159; two-component response regulator protein | -1.06 | 0.621 |
| SG0111; GI:85058089; GeneID:3867984; ubiquinone biosynthesis protein | -1.06 | 0.639 |
| SG0499; GI:85058477; GeneID:3868278; hypothetical protein | -1.06 | 0.546 |
| SG0343; GI:85058321; GeneID:3868122; ribonuclease R (RNase R) | -1.06 | 0.595 |
| SG1081; GI:85059059; GeneID:3868158; two-component sensor kinase | -1.06 | 0.677 |
| SG1994; GI:85059972; GeneID:3867075; flavodoxin II | -1.06 | 0.597 |
| SG0418; GI:85058396; GeneID:3867529; dihydrodipicolinate reductase | -1.06 | 0.611 |
| SG0238; GI:85058216; GeneID:3868290; hypothetical protein | -1.06 | 0.523 |
| SG0397; GI:85058375; GeneID:3867566; hypothetical protein | -1.06 | 0.628 |
| SG0790; GI:85058768; GeneID:3868473; transposase | -1.06 | 0.510 |
| SG2030; GI:85060008; GeneID:3866741; hypothetical protein | -1.06 | 0.555 |
| SG1690; GI:85059668; GeneID:3866273; hypothetical protein | -1.06 | 0.408 |
| SG1786; GI:85059764; GeneID:3866530; GTP-binding protein | -1.06 | 0.586 |
| SG1247; GI:85059225; GeneID:3866770; putative virulence factor | -1.06 | 0.647 |
| SG1849; GI:85059827; GeneID:3868642; putative transcriptional regulator | -1.07 | 0.610 |
| SG1414; GI:85059392; GeneID:3866975; orotidine 5'-phosphate decarboxylase | -1.07 | 0.670 |
| SG0365; GI:85058343; GeneID:3867477; hypothetical protein | -1.07 | 0.537 |
| SG1165; GI:85059143; GeneID:3866235; hypothetical protein | -1.07 | 0.628 |
| SG1772; GI:85059750; GeneID:3866516; inositol monophosphatase | -1.07 | 0.667 |
| SG1984; GI:85059962; GeneID:3866792; PTS system cellobiose-specific IIA component | -1.07 | 0.576 |
| SG2063; GI:85060041; GeneID:3866515; putative flagellar biosynthetic protein FlhB | -1.07 | 0.786 |
| SG0796; GI:85058774; GeneID:3868479; rod shape-determining protein RodA | -1.07 | 0.542 |
| SG2303; GI:85060281; GeneID:3866309; peptidyl-prolyl cis-trans isomerase A | -1.07 | 0.547 |
| SG2195; GI:85060173; GeneID:3867003; lipopolysaccharide heptosyltransferase I | -1.07 | 0.577 |
| SG2164; GI:85060142; GeneID:3867938; methionine regulon repressor | -1.07 | 0.546 |
| SG0415; GI:85058393; GeneID:3867526; prolipoprotein signal peptidase (SPase II) | -1.07 | 0.659 |
| SG0419; GI:85058397; GeneID:3867530; carbamoyl-phosphate synthase small subunit | -1.07 | 0.550 |
| SG1369; GI:85059347; GeneID:3867244; putative nucleotide sugar epimerase | -1.07 | 0.746 |
| SG1717; GI:85059695; GeneID:3866856; hypothetical protein | -1.07 | 0.660 |
| SG1245; GI:85059223; GeneID:3866768; hypothetical protein | -1.07 | 0.679 |
| SG1300; GI:85059278; GeneID:3867115; putative type III secretion apparatus | -1.07 | 0.705 |
| SG2253; GI:85060231; GeneID:3866730; RNA polymerase alpha chain | -1.07 | 0.477 |
| SG2379; GI:85060357; GeneID:3867350; putative lipopolysaccharide biosynthesis | -1.07 | 0.660 |
| SG0119; GI:85058097; GeneID:3867992; hypothetical protein | -1.07 | 0.619 |
| SG1096; GI:85059074; GeneID:3868706; arginine ABC transporter ATP-binding component | -1.07 | 0.653 |
| SG1651; GI:85059629; GeneID:3867829; hypothetical protein | -1.07 | 0.635 |
| SG0295; GI:85058273; GeneID:3868602; putative diacylglycerol kinase | -1.07 | 0.627 |
| SG0435; GI:85058413; GeneID:3867410; 3-isopropylmalate dehydratase large subunit | -1.07 | 0.561 |
| SG1641; GI:85059619; GeneID:3867164; hypothetical protein | -1.07 | 0.631 |
| SG0928; GI:85058906; GeneID:3868376; putrescine ABC transporter permease component | -1.07 | 0.618 |
| SG1053; GI:85059031; GeneID:3866965; ribosomal large subunit pseudouridine synthase | -1.07 | 0.647 |
| SG0546; GI:85058524; GeneID:3868662; 30S ribosomal protein S16 | -1.07 | 0.608 |
| SG1453; GI:85059431; GeneID:3867270; putative iron-sulfur binding NADH dehydrogenase | -1.07 | 0.651 |
| SG0883; GI:85058861; GeneID:3868331; colicin import protein TolQ | -1.07 | 0.653 |
| SG0366; GI:85058344; GeneID:3867478; putative GTP-binding protein | -1.07 | 0.692 |
| SG0219; GI:85058197; GeneID:3868051; stringent starvation protein B | -1.07 | 0.593 |
| SG2300; GI:85060278; GeneID:3866306; hypothetical protein | -1.07 | 0.688 |
| SG0015; GI:85057993; GeneID:3867672; mannitol-1-phosphate 5-dehydrogenase | -1.07 | 0.632 |
| SG0525; GI:85058503; GeneID:3868226; hypothetical protein | -1.07 | 0.615 |
| SG1040; GI:85059018; GeneID:3866952; transposase | -1.07 | 0.636 |
| SG0977; GI:85058955; GeneID:3867104; amylovoran biosynthesis protein AmsJ | -1.07 | 0.724 |
| SG1476; GI:85059454; GeneID:3866901; hypothetical protein | -1.07 | 0.854 |
| SGP1_0030; GI:85060441; GeneID:3866569; hypothetical protein | -1.07 | 0.571 |
| SG1328; GI:85059306; GeneID:3867523; L-serine deaminase | -1.07 | 0.585 |
| SG1500; GI:85059478; GeneID:3868187; hypothetical protein | -1.07 | 0.606 |
| SG2089; GI:85060067; GeneID:3866464; type III secretion apparatus InvB | -1.07 | 0.712 |
| SG0557; GI:85058535; GeneID:3867609; type III secretion apparatus | -1.07 | 0.749 |
| SG2128; GI:85060106; GeneID:3867289; single-strand binding protein | -1.07 | 0.693 |
| SG1272; GI:85059250; GeneID:3866382; phosphoribosylglycinamide formyltransferase | -1.07 | 0.688 |
| SG0417; GI:85058395; GeneID:3867528; penicillin tolerance protein (lytB) | -1.07 | 0.656 |
| SG2172; GI:85060150; GeneID:3867946; glycerol kinase | -1.07 | 0.568 |
| SG2188; GI:85060166; GeneID:3866996; hypothetical protein | -1.07 | 0.686 |
| SG2289; GI:85060267; GeneID:3866431; hypothetical protein | -1.07 | 0.728 |
| SG2429; GI:85060407; GeneID:3866325; putative preprotein translocase subunit | -1.07 | 0.590 |
| SG1766; GI:85059744; GeneID:3866925; hypothetical protein | -1.07 | 0.593 |
| SG1027; GI:85059005; GeneID:3867213; putative ATP-dependent protease | -1.07 | 0.707 |
| SG2342; GI:85060320; GeneID:3867170; DNA helicase II | -1.07 | 0.729 |
| SG1432; GI:85059410; GeneID:3866935; hypothetical protein | -1.07 | 0.675 |
| SG1692; GI:85059670; GeneID:3866275; phage holiday-junction resolvase | -1.07 | 0.602 |
| SGP3_0002; GI:85060492; GeneID:3866613; hypothetical protein | -1.07 | 0.691 |
| SG2223; GI:85060201; GeneID:3868807; guanosine-3',5'-bisbis(diphosphate) | -1.07 | 0.686 |
| SG0391; GI:85058369; GeneID:3867638; ribosomal-protein-alanine acetyltransferase | -1.07 | 0.697 |
| SG0644; GI:85058622; GeneID:3868463; hypothetical protein | -1.07 | 0.777 |
| SG1380; GI:85059358; GeneID:3867255; hypothetical protein | -1.07 | 0.679 |
| GI:125470055; GI:125470055; gp43; possible regulatory LuxR-family protein | -1.07 | 0.611 |
| SG1919; GI:85059897; GeneID:3867652; hypothetical protein | -1.07 | 0.732 |
| SG0627; GI:85058605; GeneID:3867601; hypothetical protein | -1.07 | 0.780 |
| SG0344; GI:85058322; GeneID:3868123; hypothetical protein | -1.07 | 0.679 |
| SG2393; GI:85060371; GeneID:3868092; threonine deaminase | -1.07 | 0.737 |
| SG0925; GI:85058903; GeneID:3868373; putrescine ABC transporter periplasmic | -1.08 | 0.741 |
| SG2349; GI:85060327; GeneID:3867177; hypothetical protein | -1.08 | 0.729 |
| SG1115; GI:85059093; GeneID:3868725; phosphomannomutase | -1.08 | 0.718 |
| SG0399; GI:85058377; GeneID:3867568; putative ATP-dependent protease | -1.08 | 0.771 |
| SG0069; GI:85058047; GeneID:3867493; hypothetical protein | -1.08 | 0.693 |
| SG1801; GI:85059779; GeneID:3866804; small protein A | -1.08 | 0.742 |
| SG0296; GI:85058274; GeneID:3868603; transposase | -1.08 | 0.569 |
| SG0132; GI:85058110; GeneID:3867966; 50S ribosomal protein L10 | -1.08 | 0.566 |
| SG0395; GI:85058373; GeneID:3867564; thymidine phosphorylase | -1.08 | 0.718 |
| SG2381; GI:85060359; GeneID:3867352; dTDP-D-glucose-4,6-dehydratase | -1.08 | 0.738 |
| SG1973; GI:85059951; GeneID:3866781; exodeoxyribonuclease V beta subunit | -1.08 | 0.700 |
| SG0335; GI:85058313; GeneID:3867407; DNA mismatch repair protein | -1.08 | 0.701 |
| SG0460; GI:85058438; GeneID:3868200; hypothetical protein | -1.08 | 0.738 |
| SG1874; GI:85059852; GeneID:3866348; hypothetical protein | -1.08 | 0.794 |
| SG2302; GI:85060280; GeneID:3866308; para-aminobenzoate synthase component II | -1.08 | 0.708 |
| SG1649; GI:85059627; GeneID:3867827; hypothetical protein | -1.08 | 0.620 |
| SG1897; GI:85059875; GeneID:3867770; putative transcriptional regulator | -1.08 | 0.702 |
| SGP1_0031; GI:85060442; GeneID:3866570; hypothetical protein | -1.08 | 0.704 |
| SG0885; GI:85058863; GeneID:3868333; colicin import protein TolA | -1.08 | 0.755 |
| SG2405; GI:85060383; GeneID:3868404; glucose inhibited division protein A | -1.08 | 0.661 |
| SG0534; GI:85058512; GeneID:3868650; hypothetical protein | -1.08 | 0.677 |
| SG1529; GI:85059507; GeneID:3867228; delta-aminolevulinic acid dehydratase | -1.08 | 0.744 |
| SG0588; GI:85058566; GeneID:3867702; membrane-bound lytic murein transglycosylase D | -1.08 | 0.644 |
| SG1000; GI:85058978; GeneID:3866364; putative S-adenosylmethionine-dependent | -1.08 | 0.681 |
| SG2297; GI:85060275; GeneID:3866439; hypothetical protein | -1.08 | 0.717 |
| SG0912; GI:85058890; GeneID:3868360; putative ABC transporter ATP-binding component | -1.08 | 0.704 |
| SG0869; GI:85058847; GeneID:3868317; hypothetical protein | -1.08 | 0.827 |
| SG0641; GI:85058619; GeneID:3868460; putative proline transport protein | -1.08 | 0.796 |
| SG0643; GI:85058621; GeneID:3868462; putative N-acetylmuramoyl-L-alanine amidase | -1.08 | 0.722 |
| SG0124; GI:85058102; GeneID:3867958; UDP-N-acetylenolpyruvoylglucosamine reductase | -1.08 | 0.782 |
| SG0870; GI:85058848; GeneID:3868318; putative carboxylase | -1.08 | 0.831 |
| SG2304; GI:85060282; GeneID:3866310; tryptophanyl-tRNA synthase | -1.08 | 0.804 |
| SG1569; GI:85059547; GeneID:3866682; bicyclomycin resistance protein | -1.08 | 0.745 |
| SG0832; GI:85058810; GeneID:3868774; hypothetical protein | -1.08 | 0.769 |
| SG0375; GI:85058353; GeneID:3867622; hypothetical protein | -1.08 | 0.783 |
| SG0923; GI:85058901; GeneID:3868371; glutaredoxin 1 | -1.08 | 0.813 |
| SG0914; GI:85058892; GeneID:3868362; hypothetical protein | -1.08 | 0.887 |
| SG0656; GI:85058634; GeneID:3868002; 1-deoxy-D-xylulose-5-phosphate synthase | -1.08 | 0.646 |
| SG2305; GI:85060283; GeneID:3866311; ribulose-phosphate 3-epimerase | -1.08 | 0.768 |
| SG0076; GI:85058054; GeneID:3867500; putative salicylate hydroxylase | -1.08 | 0.729 |
| SG1350; GI:85059328; GeneID:3867322; protease IV | -1.08 | 0.796 |
| SG2413; GI:85060391; GeneID:3868412; ATP synthase gamma subunit | -1.08 | 0.691 |
| SG1842; GI:85059820; GeneID:3868635; hypothetical protein | -1.08 | 0.670 |
| SG2124; GI:85060102; GeneID:3867285; putative lysR-family transcriptional regulator | -1.08 | 0.641 |
| SG1824; GI:85059802; GeneID:3866651; hypothetical protein | -1.08 | 0.738 |
| SG2082; GI:85060060; GeneID:3868113; type III secretion apparatus SpaR | -1.08 | 0.420 |
| SG1830; GI:85059808; GeneID:3868623; hypothetical protein | -1.08 | 0.678 |
| SG1543; GI:85059521; GeneID:3867242; hypothetical protein | -1.08 | 0.791 |
| SG1841; GI:85059819; GeneID:3868634; hypothetical protein | -1.08 | 0.714 |
| SG0716; GI:85058694; GeneID:3868736; phage tail/DNA circulation protein | -1.08 | 0.789 |
| SG2200; GI:85060178; GeneID:3867008; putative lipopolysaccharide glycosyltransferase | -1.08 | 0.806 |
| SG0059; GI:85058037; GeneID:3867444; RNA polymerase sigma factor for flagellar operon | -1.08 | 0.805 |
| SG0490; GI:85058468; GeneID:3868704; poly(A) polymerase I | -1.08 | 0.758 |
| SG1954; GI:85059932; GeneID:3866212; hypothetical protein | -1.08 | 0.795 |
| SG0628; GI:85058606; GeneID:3868447; magnesium transport protein | -1.08 | 0.794 |
| SG0055; GI:85058033; GeneID:3867440; flagellar protein FliT | -1.08 | 0.587 |
| SG0654; GI:85058632; GeneID:3868000; thiamin-monophosphate kinase ThiL | -1.08 | 0.782 |
| SG0308; GI:85058286; GeneID:3868576; hypothetical protein | -1.08 | 0.770 |
| SG1322; GI:85059300; GeneID:3867517; putative methyltransferase | -1.09 | 0.769 |
| SG1108; GI:85059086; GeneID:3868718; cell division protein | -1.09 | 0.821 |
| SG1026; GI:85059004; GeneID:3867212; 3-hydroxydecanoyl-(acyl-carrier-protein) | -1.09 | 0.876 |
| SG0919; GI:85058897; GeneID:3868367; hypothetical protein | -1.09 | 0.727 |
| SG2152; GI:85060130; GeneID:3868261; homoserine O-succinyltransferase | -1.09 | 0.852 |
| SG1721; GI:85059699; GeneID:3866860; putative acetyltransferase | -1.09 | 0.771 |
| SG1785; GI:85059763; GeneID:3866529; DNA repair protein RecO | -1.09 | 0.886 |
| SG0695; GI:85058673; GeneID:3868612; hypothetical protein | -1.09 | 0.828 |
| SG2386; GI:85060364; GeneID:3867357; transcription termination factor Rho | -1.09 | 0.696 |
| SG1028; GI:85059006; GeneID:3867214; hypothetical protein | -1.09 | 0.818 |
| SG2421; GI:85060399; GeneID:3868420; putative hydrolase | -1.09 | 0.773 |
| SG1052; GI:85059030; GeneID:3866964; ribonuclease E | -1.09 | 0.759 |
| SG0637; GI:85058615; GeneID:3868456; recombination associated protein RdgC | -1.09 | 0.861 |
| SG2290; GI:85060268; GeneID:3866432; hypothetical protein | -1.09 | 0.801 |
| SG1989; GI:85059967; GeneID:3867070; lysyl-tRNA synthase | -1.09 | 0.747 |
| SG1614; GI:85059592; GeneID:3867792; colicin V production protein | -1.09 | 0.786 |
| SG0583; GI:85058561; GeneID:3867697; ribosomal large subunit pseudouridine synthase | -1.09 | 0.799 |
| SG2015; GI:85059993; GeneID:3868794; phosphoglycerate kinase | -1.09 | 0.604 |
| SG1626; GI:85059604; GeneID:3867149; hypothetical protein | -1.09 | 0.844 |
| SG0254; GI:85058232; GeneID:3868306; putative O-sialoglycoprotein endopeptidase | -1.09 | 0.751 |
| SGP2_0022; GI:85060487; GeneID:3866600; hypothetical protein | -1.09 | 0.802 |
| SG0269; GI:85058247; GeneID:3867909; hypothetical protein | -1.09 | 0.794 |
| SG1424; GI:85059402; GeneID:3866985; integration host factor alpha subunit | -1.09 | 0.782 |
| SG0033; GI:85058011; GeneID:3867457; flagellar hook protein FlgE | -1.09 | 0.902 |
| SG0653; GI:85058631; GeneID:3867999; N utilization substance protein B | -1.09 | 0.791 |
| SG0379; GI:85058357; GeneID:3867626; tRNA pseudouridine synthase B | -1.09 | 0.807 |
| SG2163; GI:85060141; GeneID:3867937; cystathionine gamma-synthase | -1.09 | 0.895 |
| SG0552; GI:85058530; GeneID:3867604; hypothetical protein | -1.09 | 0.927 |
| SG1365; GI:85059343; GeneID:3867337; hypothetical protein | -1.09 | 0.805 |
| SG1465; GI:85059443; GeneID:3867282; putative transcriptional regulator | -1.09 | 0.824 |
| SG0001; GI:85057979; GeneID:3867681; chromosomal replication initiator protein DnaA | -1.09 | 0.810 |
| SG1899; GI:85059877; GeneID:3867772; spermidine N1-acetyltransferase | -1.09 | 0.758 |
| SG1225; GI:85059203; GeneID:3868678; hypothetical protein | -1.09 | 0.746 |
| SG0163; GI:85058141; GeneID:3868490; hypothetical protein | -1.09 | 0.799 |
| SG1607; GI:85059585; GeneID:3867785; phosphate acetyltransferase | -1.09 | 0.647 |
| SG0527; GI:85058505; GeneID:3868228; 2-C-methyl-D-erythritol 2,4-cyclodiphosphate | -1.09 | 0.870 |
| SG1443; GI:85059421; GeneID:3866946; transcriptional regulator | -1.09 | 0.745 |
| SG1418; GI:85059396; GeneID:3866979; hypothetical protein | -1.09 | 0.876 |
| SG2088; GI:85060066; GeneID:3866463; type III secretion apparatus SpaL/InvC | -1.09 | 0.804 |
| SG1118; GI:85059096; GeneID:3867546; hypothetical protein | -1.09 | 0.852 |
| SG1413; GI:85059391; GeneID:3866974; hypothetical protein | -1.09 | 0.865 |
| SG0933; GI:85058911; GeneID:3868381; hypothetical protein | -1.09 | 0.848 |
| SG0634; GI:85058612; GeneID:3868453; shikimate kinase II | -1.09 | 0.908 |
| GI:125470034; GI:125470034; gp18; possible DNA sensory transduction DNA-binding | -1.09 | 0.953 |
| SG2278; GI:85060256; GeneID:3866303; 50S ribosomal protein L3 | -1.09 | 0.697 |
| SG1259; GI:85059237; GeneID:3867866; hypothetical protein | -1.09 | 0.849 |
| SG1107; GI:85059085; GeneID:3868717; leucine-responsive regulatory protein | -1.09 | 0.786 |
| SG0263; GI:85058241; GeneID:3867903; 3,4-dihydroxy-2-butanone 4-phosphate synthase | -1.09 | 0.854 |
| SGP1_0034; GI:85060445; GeneID:3866573; hypothetical protein | -1.09 | 0.850 |
| SG0431; GI:85058409; GeneID:3867542; thiamine ABC transporter ATP-binding component | -1.09 | 0.793 |
| SG1246; GI:85059224; GeneID:3866769; ribosomal-protein-alanine acetyltransferase | -1.10 | 0.821 |
| SG1625; GI:85059603; GeneID:3867148; hypothetical protein | -1.10 | 0.870 |
| SG1425; GI:85059403; GeneID:3866986; lipoprotein | -1.10 | 0.867 |
| SG0166; GI:85058144; GeneID:3868493; soluble cytochrome b562 | -1.10 | 0.844 |
| SG0890; GI:85058868; GeneID:3868338; hypothetical protein | -1.10 | 0.789 |
| SG2288; GI:85060266; GeneID:3866430; hypothetical protein | -1.10 | 0.768 |
| SG1076; GI:85059054; GeneID:3866716; lipoprotein releasing system transmembrane | -1.10 | 0.811 |
| SG1256; GI:85059234; GeneID:3867863; putative isochorismatase | -1.10 | 0.861 |
| SG2104; GI:85060082; GeneID:3866245; putative phage integrase | -1.10 | 0.823 |
| SG0491; GI:85058469; GeneID:3868705; putative glutamyl-tRNA synthase | -1.10 | 0.901 |
| SG0911; GI:85058889; GeneID:3868359; putative 1-aminocyclopropane-1-carboxylate | -1.10 | 0.861 |
| SG2182; GI:85060160; GeneID:3866990; putative RNA methyltransferase | -1.10 | 0.887 |
| SG2237; GI:85060215; GeneID:3867365; hypothetical protein | -1.10 | 0.869 |
| SG1961; GI:85059939; GeneID:3866219; N-acetylmuramoyl-L-alanine amidase | -1.10 | 0.886 |
| SG1540; GI:85059518; GeneID:3867239; hemin ABC transporter ATP-binding component | -1.10 | 0.840 |
| SG2366; GI:85060344; GeneID:3866823; porphobilinogen deaminase | -1.10 | 0.876 |
| SG1575; GI:85059553; GeneID:3866688; hypothetical protein | -1.10 | 0.889 |
| SG0114; GI:85058092; GeneID:3867987; sec-independent protein translocase protein | -1.10 | 0.876 |
| SG0717; GI:85058695; GeneID:3868737; hypothetical protein | -1.10 | 0.801 |
| SG2203; GI:85060181; GeneID:3868748; 3-deoxy-manno-octulosonic acid transferase | -1.10 | 0.902 |
| SG0218; GI:85058196; GeneID:3868089; stringent starvation protein A | -1.10 | 0.907 |
| SG1086; GI:85059064; GeneID:3868163; hypothetical protein | -1.10 | 0.883 |
| SG2221; GI:85060199; GeneID:3868805; guanylate kinase | -1.10 | 0.770 |
| SG1613; GI:85059591; GeneID:3867791; amidophosphoribosyltransferase | -1.10 | 0.875 |
| SG1942; GI:85059920; GeneID:3867854; elongation factor EF-Ts | -1.10 | 0.771 |
| SG0131; GI:85058109; GeneID:3867965; 50S ribosomal protein L1 | -1.10 | 0.740 |
| SG1180; GI:85059158; GeneID:3868551; hypothetical protein | -1.10 | 0.701 |
| SG1823; GI:85059801; GeneID:3866650; hypothetical protein | -1.10 | 0.964 |
| SG2130; GI:85060108; GeneID:3867291; hypothetical protein | -1.10 | 0.904 |
| SG1992; GI:85059970; GeneID:3867073; thiol:disulfide interchange protein | -1.10 | 0.872 |
| SG0859; GI:85058837; GeneID:3867817; PTS system N-acetylglucosamine-specific IIABC | -1.10 | 0.875 |
| SG2087; GI:85060065; GeneID:3866462; type III secretion apparatus SpaM | -1.10 | 0.849 |
| SG1113; GI:85059091; GeneID:3868723; UTP-glucose-1-phosphate uridylyltransferase | -1.10 | 0.871 |
| SG0309; GI:85058287; GeneID:3868577; elongation factor P | -1.10 | 0.845 |
| SG1541; GI:85059519; GeneID:3867240; hypothetical protein | -1.10 | 0.876 |
| SG1441; GI:85059419; GeneID:3866944; ribonuclease T | -1.10 | 0.907 |
| SG1875; GI:85059853; GeneID:3866349; putative protoporphyrinogen oxidase | -1.10 | 0.864 |
| SG2306; GI:85060284; GeneID:3866312; DNA adenine methylase Dam | -1.10 | 0.962 |
| SG1616; GI:85059594; GeneID:3867794; folylpolyglutamate synthase | -1.10 | 0.946 |
| SG1778; GI:85059756; GeneID:3866522; phosphoribosylformyl-glycinamidine synthase | -1.10 | 0.842 |
| SG0348; GI:85058326; GeneID:3868127; 50S ribosomal protein L9 | -1.10 | 0.656 |
| SG1396; GI:85059374; GeneID:3867036; hypothetical protein | -1.10 | 0.885 |
| SG0429; GI:85058407; GeneID:3867540; ribosomal large subunit pseudouridine synthase | -1.10 | 0.911 |
| SG0270; GI:85058248; GeneID:3867910; hypothetical protein | -1.10 | 0.836 |
| SG0135; GI:85058113; GeneID:3867969; RNA polymerase beta prime subunit | -1.10 | 0.745 |
| SG0168; GI:85058146; GeneID:3868495; hypothetical protein | -1.10 | 0.860 |
| SG1787; GI:85059765; GeneID:3866531; ribonuclease III | -1.10 | 0.839 |
| SG2294; GI:85060272; GeneID:3866436; hypothetical protein | -1.10 | 0.773 |
| SG1134; GI:85059112; GeneID:3867562; hypothetical protein | -1.10 | 0.690 |
| SG1469; GI:85059447; GeneID:3866894; hypothetical protein | -1.10 | 0.868 |
| SG2235; GI:85060213; GeneID:3867363; DNA polymerase I | -1.10 | 0.875 |
| SG1064; GI:85059042; GeneID:3866704; hypothetical protein | -1.10 | 0.834 |
| SG0378; GI:85058356; GeneID:3867625; ribosome-binding factor A | -1.10 | 0.846 |
| SG0440; GI:85058418; GeneID:3867415; hypothetical protein | -1.10 | 0.761 |
| SG0277; GI:85058255; GeneID:3868584; putative cell division protein | -1.10 | 0.886 |
| SGP2_0013; GI:85060479; GeneID:3866591; hypothetical protein | -1.10 | 0.730 |
| SG1255; GI:85059233; GeneID:3867862; hypothetical protein | -1.10 | 0.915 |
| SG0301; GI:85058279; GeneID:3868569; thiol-disulfide interchange protein | -1.10 | 0.867 |
| SG1230; GI:85059208; GeneID:3868683; hypothetical protein | -1.10 | 0.934 |
| SG2108; GI:85060086; GeneID:3866249; putative transport protein | -1.10 | 0.929 |
| SG1751; GI:85059729; GeneID:3866496; malate:quinone oxidoreductase | -1.10 | 0.844 |
| SG0183; GI:85058161; GeneID:3866622; L-fuculose-1-phosphate aldolase | -1.10 | 0.848 |
| SG0272; GI:85058250; GeneID:3867912; putative sugar aldolase | -1.10 | 0.883 |
| SG0574; GI:85058552; GeneID:3867688; putative translocation machinery component | -1.11 | 0.904 |
| SG2208; GI:85060186; GeneID:3868753; 50S ribosomal protein L28 | -1.11 | 0.815 |
| SG1741; GI:85059719; GeneID:3866486; hypothetical protein | -1.11 | 0.855 |
| SG0867; GI:85058845; GeneID:3868315; hypothetical protein | -1.11 | 0.919 |
| SG1403; GI:85059381; GeneID:3867043; hypothetical protein | -1.11 | 0.880 |
| SG1705; GI:85059683; GeneID:3866451; cysteine synthase B | -1.11 | 0.856 |
| SG0455; GI:85058433; GeneID:3868195; hypothetical protein | -1.11 | 0.781 |
| SG2338; GI:85060316; GeneID:3867166; outer membrane phospholipase A | -1.11 | 0.917 |
| SG2201; GI:85060179; GeneID:3868746; putative lipopolysaccharide glucosyltransferase | -1.11 | 0.911 |
| SG0121; GI:85058099; GeneID:3867955; protoporphyrinogen oxidase | -1.11 | 0.903 |
| SG1276; GI:85059254; GeneID:3866386; putative copper resistance protein | -1.11 | 0.907 |
| SG2019; GI:85059997; GeneID:3868798; S-adenosylmethionine synthase | -1.11 | 0.853 |
| SG0079; GI:85058057; GeneID:3867503; hypothetical protein | -1.11 | 0.864 |
| SG0863; GI:85058841; GeneID:3868311; hypothetical protein | -1.11 | 0.908 |
| SG0813; GI:85058791; GeneID:3868022; asparagine synthase B | -1.11 | 0.916 |
| SG0998; GI:85058976; GeneID:3866891; 3-deoxy-manno-octulosonate cytidylyltransferase | -1.11 | 0.939 |
| SG0139; GI:85058117; GeneID:3867973; endonuclease V (deoxyinosine 3'endoduclease) | -1.11 | 0.897 |
| SG0972; GI:85058950; GeneID:3867099; hypothetical protein | -1.11 | 0.881 |
| SG2337; GI:85060315; GeneID:3867165; ATP-dependent DNA helicase RecQ | -1.11 | 0.920 |
| SG2327; GI:85060305; GeneID:3867312; glycerol-3-phosphate regulon protein GlpG | -1.11 | 0.939 |
| SGP2_0012; GI:85060478; GeneID:3866590; replication associated protein RepA1 | -1.11 | 0.740 |
| SG0591; GI:85058569; GeneID:3866659; ribonuclease H | -1.11 | 0.875 |
| SG1630; GI:85059608; GeneID:3867153; phosphohistidine phosphatase | -1.11 | 0.866 |
| SG2096; GI:85060074; GeneID:3866471; hypothetical protein | -1.11 | 0.906 |
| SG0353; GI:85058331; GeneID:3868132; hypothetical protein | -1.11 | 0.852 |
| SG1119; GI:85059097; GeneID:3867547; mannosyltransferase A | -1.11 | 0.892 |
| SG2218; GI:85060196; GeneID:3868763; putative phospholipase A | -1.11 | 0.823 |
| SG1781; GI:85059759; GeneID:3866525; hypothetical protein | -1.11 | 0.875 |
| SG0702; GI:85058680; GeneID:3868619; phosphoribosylaminoimidazole carboxylase | -1.11 | 0.946 |
| SG2383; GI:85060361; GeneID:3867354; UDP-N-acetylglucosamine 2-epimerase | -1.11 | 0.957 |
| SG0810; GI:85058788; GeneID:3868019; putative ATP-binding protein in pho regulon | -1.11 | 0.898 |
| SGP1_0035; GI:85060446; GeneID:3866574; achromobactin ABC transporter ATP-binding | -1.11 | 0.871 |
| SG0152; GI:85058130; GeneID:3868518; biotin carboxylase | -1.11 | 0.922 |
| SG1261; GI:85059239; GeneID:3867868; holliday junction DNA helicase RuvA | -1.11 | 0.928 |
| SG1409; GI:85059387; GeneID:3866970; cysteine regulon transcriptional activator | -1.11 | 0.947 |
| SG1439; GI:85059417; GeneID:3866942; riboflavin synthase alpha chain | -1.11 | 0.845 |
| SG1915; GI:85059893; GeneID:3867648; ABC transporter ATP-binding component | -1.11 | 0.856 |
| SG1921; GI:85059899; GeneID:3867654; hypothetical protein | -1.11 | 0.910 |
| SG1974; GI:85059952; GeneID:3866782; protease III precursor | -1.11 | 0.943 |
| SG0377; GI:85058355; GeneID:3867624; translation initiation factor IF-2 | -1.11 | 0.904 |
| SG2245; GI:85060223; GeneID:3866722; hypothetical protein | -1.11 | 0.876 |
| SG0941; GI:85058919; GeneID:3868428; hypothetical protein | -1.11 | 0.869 |
| SG1184; GI:85059162; GeneID:3868555; hypothetical protein | -1.11 | 0.800 |
| SG0088; GI:85058066; GeneID:3867748; hypothetical protein | -1.11 | 0.887 |
| SG2272; GI:85060250; GeneID:3866297; 30S ribosomal protein S3 | -1.11 | 0.783 |
| SG1252; GI:85059230; GeneID:3867859; hypothetical protein | -1.11 | 0.892 |
| SG1121; GI:85059099; GeneID:3867549; glycosyltransferase | -1.11 | 0.911 |
| SG0285; GI:85058263; GeneID:3868592; transcriptional activator protein | -1.11 | 0.860 |
| SG1339; GI:85059317; GeneID:3866410; hypothetical protein | -1.11 | 0.921 |
| SG0170; GI:85058148; GeneID:3868497; hypothetical protein | -1.12 | 0.981 |
| SG0956; GI:85058934; GeneID:3868443; putative esterase | -1.12 | 0.925 |
| SG0369; GI:85058347; GeneID:3867481; hypothetical protein | -1.12 | 0.955 |
| SG1353; GI:85059331; GeneID:3867325; hypothetical protein | -1.12 | 0.931 |
| SG2264; GI:85060242; GeneID:3866289; 30S ribosomal protein S8 | -1.12 | 0.778 |
| SGP1_0028; GI:85060439; GeneID:3866567; hypothetical protein | -1.12 | 0.810 |
| SG0065; GI:85058043; GeneID:3867489; hypothetical protein | -1.12 | 0.836 |
| SG1581; GI:85059559; GeneID:3866694; capsular synthesis sensor protein RcsC | -1.12 | 0.924 |
| SG0260; GI:85058238; GeneID:3867900; glutamate-ammonia-ligase adenylyltransferase | -1.12 | 0.965 |
| SG2293; GI:85060271; GeneID:3866435; FKBP-type peptidyl-prolyl cis-trans isomerase | -1.12 | 0.920 |
| SG1411; GI:85059389; GeneID:3866972; phosphatidylglycerophosphatase B | -1.12 | 0.910 |
| SG1274; GI:85059252; GeneID:3866384; DNA polymerase III theta subunit | -1.12 | 0.899 |
| SGtRNA69; GeneID:3868646; tRNA-Met | -1.12 | 0.744 |
| SG0881; GI:85058859; GeneID:3868329; cytochrome d ubiquinol oxidase subunit II | -1.12 | 0.867 |
| SG2141; GI:85060119; GeneID:3867302; lexA repressor | -1.12 | 0.845 |
| SG0590; GI:85058568; GeneID:3866658; hypothetical protein | -1.12 | 0.817 |
| SGP1_0033; GI:85060444; GeneID:3866572; putative demethylmenaquinone methyltransferase | -1.12 | 0.922 |
| SG1632; GI:85059610; GeneID:3867155; lipoprotein precursor | -1.12 | 0.919 |
| SG1933; GI:85059911; GeneID:3867845; UDP-3-O-(3-hydroxymyristoyl) glucosamine | -1.12 | 0.905 |
| SG0130; GI:85058108; GeneID:3867964; 50S ribosomal protein L11 | -1.12 | 0.877 |
| SG0235; GI:85058213; GeneID:3868067; putative transport protein | -1.12 | 0.954 |
| SG2048; GI:85060026; GeneID:3866500; hypothetical protein | -1.12 | 0.904 |
| SG1978; GI:85059956; GeneID:3866786; prolipoprotein diacylglyceryl transferase | -1.12 | 0.928 |
| SG1720; GI:85059698; GeneID:3866859; hypothetical protein | -1.12 | 0.960 |
| SG0141; GI:85058119; GeneID:3868507; DNA-binding protein HU-alpha (HU-2) | -1.12 | 0.745 |
| SG1336; GI:85059314; GeneID:3866407; cell division inhibitor | -1.12 | 0.942 |
| SG0744; GI:85058722; GeneID:3867721; phage DNA methyltransferase | -1.12 | 0.812 |
| SG0202; GI:85058180; GeneID:3868073; hypothetical protein | -1.12 | 0.938 |
| SG0165; GI:85058143; GeneID:3868492; hypothetical protein | -1.12 | 0.949 |
| SG1273; GI:85059251; GeneID:3866383; protease II | -1.13 | 0.971 |
| SG1062; GI:85059040; GeneID:3866702; 3-oxoacyl-(acyl-carrier-protein) synthase II | -1.13 | 0.936 |
| SG1938; GI:85059916; GeneID:3867850; undecaprenyl pyrophosphate synthase | -1.13 | 0.944 |
| SG0403; GI:85058381; GeneID:3867572; aerobic respiration control protein | -1.13 | 0.913 |
| SG1940; GI:85059918; GeneID:3867852; ribosome recycling factor | -1.13 | 0.928 |
| SG1485; GI:85059463; GeneID:3866910; hypothetical protein | -1.13 | 0.960 |
| SG0739; GI:85058717; GeneID:3867716; hypothetical protein | -1.13 | 0.922 |
| SG1452; GI:85059430; GeneID:3867269; hypothetical protein | -1.13 | 0.947 |
| SG2046; GI:85060024; GeneID:3866498; hypothetical protein | -1.13 | 0.972 |
| SG0010; GI:85057988; GeneID:3867667; hypothetical protein | -1.13 | 0.963 |
| SG2170; GI:85060148; GeneID:3867944; menaquinone biosynthesis protein | -1.13 | 0.946 |
| SG1980; GI:85059958; GeneID:3866788; hypothetical protein | -1.13 | 0.943 |
| SG0278; GI:85058256; GeneID:3868585; putative 2,5-diketo-D-gluconate reductase A | -1.13 | 0.937 |
| SG0918; GI:85058896; GeneID:3868366; putative ABC transporter ATP-binding component | -1.13 | 0.935 |
| SG0448; GI:85058426; GeneID:3867423; cell division protein FtsW | -1.13 | 0.948 |
| SG1894; GI:85059872; GeneID:3867767; hypothetical protein | -1.13 | 0.944 |
| SG0220; GI:85058198; GeneID:3868052; aerobic respiration sensor-response protein | -1.13 | 0.976 |
| SG0162; GI:85058140; GeneID:3868489; putative transcriptional regulator | -1.13 | 0.971 |
| SG1172; GI:85059150; GeneID:3866242; phosphatidylglycerophosphate synthase | -1.13 | 0.955 |
| SG1513; GI:85059491; GeneID:3868239; enoyl-(acyl-carrier-protein) reductase (NADH) | -1.13 | 0.952 |
| SG0907; GI:85058885; GeneID:3868355; excision nuclease subunit B | -1.13 | 0.967 |
| SG0174; GI:85058152; GeneID:3868501; gamma-aminobutyrate transport protein | -1.13 | 0.824 |
| SG2171; GI:85060149; GeneID:3867945; hypothetical protein | -1.13 | 0.762 |
| SG0252; GI:85058230; GeneID:3868304; DNA primase | -1.13 | 0.979 |
| SG0526; GI:85058504; GeneID:3868227; 2-C-methyl-D-erythritol 4-phosphate | -1.13 | 0.983 |
| SG2243; GI:85060221; GeneID:3866720; hypothetical protein | -1.13 | 0.962 |
| SG2369; GI:85060347; GeneID:3866826; hypothetical protein | -1.13 | 0.988 |
| SG2380; GI:85060358; GeneID:3867351; glucose-1-phosphate thymidylyltransferase | -1.13 | 0.969 |
| SG2250; GI:85060228; GeneID:3866727; large-conductance mechanosensitive channel | -1.13 | 0.970 |
| SG0175; GI:85058153; GeneID:3868502; glutamate dehydrogenase | -1.13 | 0.939 |
| SG0313; GI:85058291; GeneID:3868581; hypothetical protein | -1.13 | 0.984 |
| SG0596; GI:85058574; GeneID:3866664; hypothetical protein | -1.13 | 0.958 |
| SG0955; GI:85058933; GeneID:3868442; lysine transport protein | -1.13 | 0.986 |
| SG2259; GI:85060237; GeneID:3866284; 50S ribosomal protein L15 | -1.13 | 0.847 |
| SG1617; GI:85059595; GeneID:3867795; acetyl-CoA carboxylase beta subunit | -1.13 | 0.959 |
| SG1066; GI:85059044; GeneID:3866706; DNA polymerase III delta prime subunit | -1.13 | 0.979 |
| SG0442; GI:85058420; GeneID:3867417; cell division protein FtsL | -1.13 | 0.889 |
| SG0424; GI:85058402; GeneID:3867535; dimethyladenosine transferase | -1.13 | 0.978 |
| SG0150; GI:85058128; GeneID:3868516; DNA-binding protein Fis | -1.13 | 0.970 |
| SG1756; GI:85059734; GeneID:3866915; putative GTP-binding protein | -1.14 | 0.983 |
| SG0416; GI:85058394; GeneID:3867527; FKBP-type peptidyl-prolyl cis-trans isomerase | -1.14 | 0.971 |
| SG0565; GI:85058543; GeneID:3867617; type III secretion apparatus SpaL/InvC | -1.14 | 0.980 |
| SG2228; GI:85060206; GeneID:3868812; deoxyribose operon repressor | -1.14 | 0.980 |
| SG0161; GI:85058139; GeneID:3868488; putative modulator of DNA gyrase TldD | -1.14 | 0.972 |
| SG0677; GI:85058655; GeneID:3868144; ABC transporter ATP-binding component | -1.14 | 0.971 |
| SG1624; GI:85059602; GeneID:3867147; hypothetical protein | -1.14 | 0.984 |
| SG2378; GI:85060356; GeneID:3867349; putative lipopolysaccharide biosynthesis | -1.14 | 0.980 |
| SG1711; GI:85059689; GeneID:3866457; putative acetyltransferase | -1.14 | 0.963 |
| SG2160; GI:85060138; GeneID:3868269; acetylglutamate kinase | -1.14 | 0.980 |
| SG0352; GI:85058330; GeneID:3868131; hypothetical protein | -1.14 | 0.984 |
| SG2374; GI:85060352; GeneID:3866831; putative UDP-N-acetyl-D-mannosaminuronic acid | -1.14 | 0.993 |
| SG1694; GI:85059672; GeneID:3866277; hypothetical protein | -1.14 | 0.866 |
| SG1410; GI:85059388; GeneID:3866971; GTP cyclohydrolase II | -1.14 | 0.970 |
| SG1943; GI:85059921; GeneID:3867855; 30S ribosomal protein S2 | -1.14 | 0.945 |
| SG1678; GI:85059656; GeneID:3868542; DNA ligase | -1.14 | 0.992 |
| SG2396; GI:85060374; GeneID:3868388; acetolactate synthase isozyme II small subunit | -1.14 | 0.916 |
| SG1928; GI:85059906; GeneID:3867840; DNA polymerase III alpha subunit | -1.14 | 0.985 |
| SG1045; GI:85059023; GeneID:3866957; glucans biosynthesis protein | -1.14 | 0.980 |
| SG0958; GI:85058936; GeneID:3868445; hypothetical protein | -1.14 | 0.908 |
| SG0267; GI:85058245; GeneID:3867907; putative ADP-ribose pyrophosphatase | -1.14 | 0.983 |
| SG1101; GI:85059079; GeneID:3868711; hypothetical protein | -1.14 | 0.925 |
| SG2143; GI:85060121; GeneID:3868252; 4-hydroxybenzoate octaprenyltransferase | -1.14 | 0.967 |
| SG1185; GI:85059163; GeneID:3868556; hypothetical protein | -1.14 | 0.818 |
| SG2332; GI:85060310; GeneID:3867317; high-affinity gluconate transport protein | -1.14 | 0.987 |
| SG1451; GI:85059429; GeneID:3867268; hypothetical protein | -1.14 | 0.978 |
| SG2040; GI:85060018; GeneID:3866751; hypothetical protein | -1.14 | 0.980 |
| SG1254; GI:85059232; GeneID:3867861; hypothetical protein | -1.14 | 0.971 |
| SG1275; GI:85059253; GeneID:3866385; ferritin | -1.14 | 0.914 |
| SG0908; GI:85058886; GeneID:3868356; hypothetical protein | -1.14 | 0.984 |
| SGP2_0019; GI:85060485; GeneID:3866597; putative twitching motility protein PilT | -1.14 | 0.963 |
| SG1520; GI:85059498; GeneID:3868246; hypothetical protein | -1.14 | 0.989 |
| SG1437; GI:85059415; GeneID:3866940; major outer membrane lipoprotein | -1.14 | 0.780 |
| SG0336; GI:85058314; GeneID:3868115; tRNA delta(2)-isopentenylpyrophosphate | -1.14 | 0.946 |
| SG2210; GI:85060188; GeneID:3868755; deoxyuridine 5'-triphosphate | -1.15 | 0.963 |
| SG1055; GI:85059033; GeneID:3866967; hypothetical protein | -1.15 | 0.968 |
| SG2173; GI:85060151; GeneID:3867947; multidrug efflux transport protein D | -1.15 | 0.949 |
| SG1173; GI:85059151; GeneID:3866243; phage integrase | -1.15 | 0.873 |
| SG1491; GI:85059469; GeneID:3868178; ATP-dependent helicase | -1.15 | 0.983 |
| SG0016; GI:85057994; GeneID:3867673; mannitol operon repressor | -1.15 | 0.960 |
| SG0385; GI:85058363; GeneID:3867632; hypothetical protein | -1.15 | 0.974 |
| SG2144; GI:85060122; GeneID:3868253; putative chorismate lyase | -1.15 | 0.973 |
| SG1085; GI:85059063; GeneID:3868162; tRNA (5-methylaminomethyl-2-thiouridylate)- | -1.15 | 0.976 |
| SG1094; GI:85059072; GeneID:3868171; arginine ABC transporter permease component | -1.15 | 0.982 |
| SG1054; GI:85059032; GeneID:3866966; hypothetical protein | -1.15 | 0.976 |
| SG2012; GI:85059990; GeneID:3868791; hypothetical protein | -1.15 | 0.966 |
| SG1747; GI:85059725; GeneID:3866492; hypothetical protein | -1.15 | 0.719 |
| SG0394; GI:85058372; GeneID:3867563; deoxyribose-phosphate aldolase | -1.15 | 0.989 |
| SG2390; GI:85060368; GeneID:3867361; ATP-dependent DNA helicase Rep | -1.15 | 0.990 |
| SG1679; GI:85059657; GeneID:3868543; putative cell division protein | -1.15 | 0.971 |
| SG0652; GI:85058630; GeneID:3867998; riboflavin synthase | -1.15 | 0.979 |
| SG1267; GI:85059245; GeneID:3867874; hypothetical protein | -1.15 | 0.993 |
| SG0363; GI:85058341; GeneID:3867475; 50S ribosomal protein L21 | -1.15 | 0.945 |
| SG1120; GI:85059098; GeneID:3867548; mannosyltransferase B | -1.15 | 0.982 |
| SG2107; GI:85060085; GeneID:3866248; putative transport protein | -1.15 | 0.988 |
| SG1782; GI:85059760; GeneID:3866526; putative ferredoxin | -1.15 | 0.990 |
| SG2318; GI:85060296; GeneID:3867303; two-component sensor kinase | -1.15 | 0.970 |
| SG1058; GI:85059036; GeneID:3866698; 3-oxoacyl-(acyl-carrier-protein) synthase III | -1.15 | 0.976 |
| SG0382; GI:85058360; GeneID:3867629; hypothetical protein | -1.15 | 0.925 |
| SG0961; GI:85058939; GeneID:3867088; hypothetical protein | -1.15 | 0.977 |
| SG1780; GI:85059758; GeneID:3866524; hypothetical protein | -1.15 | 0.994 |
| SG1696; GI:85059674; GeneID:3866279; hypothetical protein | -1.15 | 0.981 |
| SG1253; GI:85059231; GeneID:3867860; hypothetical protein | -1.15 | 0.977 |
| SG0441; GI:85058419; GeneID:3867416; hypothetical protein | -1.15 | 0.983 |
| SG2222; GI:85060200; GeneID:3868806; RNA polymerase omega subunit | -1.15 | 0.944 |
| SG0587; GI:85058565; GeneID:3867701; hypothetical protein | -1.15 | 0.978 |
| SG0217; GI:85058195; GeneID:3868088; 30S ribosomal protein S9 | -1.16 | 0.964 |
| SG0676; GI:85058654; GeneID:3868143; hypothetical protein | -1.16 | 0.990 |
| SG1370; GI:85059348; GeneID:3867245; histone-like protein | -1.16 | 0.926 |
| SG2121; GI:85060099; GeneID:3866262; proton/sodium-glutamate/aspartate transport | -1.16 | 0.982 |
| SG1901; GI:85059879; GeneID:3867774; hypothetical protein | -1.16 | 0.978 |
| SG0669; GI:85058647; GeneID:3868136; cell division protein BolA | -1.16 | 0.977 |
| SG2295; GI:85060273; GeneID:3866437; potassium-hydrogen antiporter | -1.16 | 0.988 |
| SG0253; GI:85058231; GeneID:3868305; 30S ribosomal protein S21 | -1.16 | 0.966 |
| SG2407; GI:85060385; GeneID:3868406; ATP synthase subunit I | -1.16 | 0.956 |
| SG1346; GI:85059324; GeneID:3866417; hypothetical protein | -1.16 | 0.960 |
| GI:125470043; GI:125470043; gp28; probable HU-like DNA-binding protein; similar to | -1.16 | 0.989 |
| SG0292; GI:85058270; GeneID:3868599; putative sulfate/molybdate ABC transporter | -1.16 | 0.956 |
| SG1074; GI:85059052; GeneID:3866714; hypothetical protein | -1.16 | 0.977 |
| SG2422; GI:85060400; GeneID:3868421; PTS system mannose/fructose/sorbose-specific IIB | -1.16 | 0.923 |
| SG0945; GI:85058923; GeneID:3868432; hypothetical protein | -1.16 | 0.988 |
| SG2260; GI:85060238; GeneID:3866285; 50S ribosomal protein L30 | -1.16 | 0.935 |
| SG0364; GI:85058342; GeneID:3867476; 50S ribosomal protein L27 | -1.17 | 0.974 |
| SG2367; GI:85060345; GeneID:3866824; uroporphyrinogen III synthase | -1.17 | 0.985 |
| SG1262; GI:85059240; GeneID:3867869; holliday junction DNA helicase RuvB | -1.17 | 0.994 |
| SGtRNA01; GeneID:3866337; tRNA-Pro | -1.17 | 0.972 |
| SG1731; GI:85059709; GeneID:3866870; uracil phosphoribosyltransferase | -1.17 | 0.987 |
| SG0558; GI:85058536; GeneID:3867610; type III secretion apparatus | -1.17 | 0.994 |
| SG0294; GI:85058272; GeneID:3868601; hypothetical protein | -1.17 | 0.991 |
| SG1645; GI:85059623; GeneID:3867823; putative phage antiterminator Q protein | -1.17 | 0.981 |
| SG2212; GI:85060190; GeneID:3868757; orotate phosphoribosyltransferase | -1.17 | 0.990 |
| SG0458; GI:85058436; GeneID:3868198; mutator protein MutT | -1.17 | 0.985 |
| SG0100; GI:85058078; GeneID:3867760; putative N-acetylmuramoyl-L-alanine amidase | -1.17 | 0.985 |
| SG0645; GI:85058623; GeneID:3868464; S-adenosylmethionine:tRNA | -1.17 | 0.983 |
| SG2002; GI:85059980; GeneID:3867083; aminomethyltransferase | -1.17 | 0.997 |
| SG2211; GI:85060189; GeneID:3868756; tetR-family transcriptional regulator | -1.17 | 0.980 |
| SG2274; GI:85060252; GeneID:3866299; 30S ribosomal protein S19 | -1.17 | 0.937 |
| SG1189; GI:85059167; GeneID:3868560; hypothetical protein | -1.17 | 0.917 |
| SG2340; GI:85060318; GeneID:3867168; chloramphenicol-sensitive protein RarD | -1.17 | 0.989 |
| SG0084; GI:85058062; GeneID:3867744; cell division protein | -1.17 | 0.998 |
| SG1059; GI:85059037; GeneID:3866699; malonyl CoA-(acyl-carrier-protein) transacylase | -1.17 | 0.991 |
| SG1393; GI:85059371; GeneID:3867033; hypothetical protein | -1.17 | 0.992 |
| SG0480; GI:85058458; GeneID:3868694; hypothetical protein | -1.17 | 0.995 |
| SG0649; GI:85058627; GeneID:3867995; protein-export membrane protein SecF | -1.17 | 0.996 |
| SG1116; GI:85059094; GeneID:3867544; ABC transporter permease component | -1.18 | 0.984 |
| SG1073; GI:85059051; GeneID:3866713; NADH dehydrogenase | -1.18 | 0.969 |
| SG1060; GI:85059038; GeneID:3866700; 3-oxoacyl-(acyl-carrier-protein) reductase | -1.18 | 0.986 |
| SG2430; GI:85060408; GeneID:3866326; hypothetical protein | -1.18 | 0.986 |
| SGP1_0001; GI:85060412; GeneID:3866540; putative regulatory protein | -1.18 | 0.924 |
| SG0994; GI:85058972; GeneID:3866887; integration host factor beta-subunit | -1.18 | 0.988 |
| SG2395; GI:85060373; GeneID:3868094; branched-chain amino-acid aminotransferase | -1.18 | 0.996 |
| SG1976; GI:85059954; GeneID:3866784; hypothetical protein | -1.18 | 0.984 |
| SG0140; GI:85058118; GeneID:3867974; hypothetical protein | -1.18 | 0.998 |
| SG1682; GI:85059660; GeneID:3866265; phage lysis protein S | -1.18 | 0.961 |
| SG0973; GI:85058951; GeneID:3867100; uridine kinase | -1.18 | 0.996 |
| SG2431; GI:85060409; GeneID:3866327; ribonuclease P protein | -1.18 | 0.943 |
| SG0019; GI:85057997; GeneID:3867676; putative transport protein | -1.18 | 0.995 |
| SG2148; GI:85060126; GeneID:3868257; putative transport protein | -1.18 | 0.992 |
| SG2415; GI:85060393; GeneID:3868414; ATP synthase epsilon subunit | -1.18 | 0.985 |
| SG1793; GI:85059771; GeneID:3866796; RNA polymerase sigma-E factor | -1.18 | 0.976 |
| SG0396; GI:85058374; GeneID:3867565; phosphopentomutase | -1.18 | 0.997 |
| SG0428; GI:85058406; GeneID:3867539; DnaJ-like protein | -1.18 | 0.996 |
| SG1677; GI:85059655; GeneID:3868541; putative transposase | -1.18 | 0.995 |
| SG2404; GI:85060382; GeneID:3868403; MioC (initiation of chromosome replication) | -1.18 | 0.998 |
| SG0322; GI:85058300; GeneID:3867394; hypothetical protein | -1.19 | 0.990 |
| SG0064; GI:85058042; GeneID:3867488; dipeptide ABC transporter ATP-binding component | -1.19 | 0.994 |
| SG1927; GI:85059905; GeneID:3867660; acetyl-coenzyme A carboxylase carboxyl | -1.19 | 0.986 |
| SG0129; GI:85058107; GeneID:3867963; transcription antitermination protein | -1.19 | 0.969 |
| SGP2_0018; GI:85060484; GeneID:3866596; hypothetical protein | -1.19 | 0.991 |
| SG2397; GI:85060375; GeneID:3868389; acetolactate synthase isozyme II large subunit | -1.19 | 0.999 |
| SG0916; GI:85058894; GeneID:3868364; putative ATP-dependent helicase | -1.19 | 0.998 |
| SG1186; GI:85059164; GeneID:3868557; hypothetical protein | -1.19 | 0.932 |
| SG0107; GI:85058085; GeneID:3867980; uridine phosphorylase | -1.19 | 0.993 |
| SG0954; GI:85058932; GeneID:3868441; 1-phosphofructokinase | -1.19 | 0.947 |
| SG2406; GI:85060384; GeneID:3868405; glucose inhibited division protein B | -1.19 | 0.997 |
| SG0658; GI:85058636; GeneID:3868004; exodeoxyribonuclease VII small subunit | -1.19 | 0.999 |
| SG0354; GI:85058332; GeneID:3868133; hypothetical protein | -1.19 | 0.983 |
| SG0783; GI:85058761; GeneID:3868050; hypothetical protein | -1.19 | 0.999 |
| SG1688; GI:85059666; GeneID:3866271; hypothetical protein | -1.19 | 0.931 |
| SG2258; GI:85060236; GeneID:3866735; preprotein translocase SecY subunit | -1.19 | 0.972 |
| SG1730; GI:85059708; GeneID:3866869; hypothetical protein | -1.19 | 0.999 |
| SG0333; GI:85058311; GeneID:3867405; hypothetical protein | -1.20 | 0.999 |
| SG1910; GI:85059888; GeneID:3867643; hypothetical protein | -1.20 | 0.993 |
| SG0630; GI:85058608; GeneID:3868449; hypothetical protein | -1.20 | 0.997 |
| SG2271; GI:85060249; GeneID:3866296; 50S ribosomal protein L16 | -1.20 | 0.968 |
| SG1917; GI:85059895; GeneID:3867650; putative lipoprotein | -1.20 | 0.997 |
| SG2341; GI:85060319; GeneID:3867169; magnesium transport protein | -1.20 | 0.998 |
| SG1065; GI:85059043; GeneID:3866705; thymidylate kinase | -1.20 | 0.999 |
| SG1979; GI:85059957; GeneID:3866787; phosphoenolpyruvate-protein phosphotransferase | -1.20 | 0.999 |
| SG1988; GI:85059966; GeneID:3867069; diaminopimelate decarboxylase | -1.20 | 0.998 |
| SG2273; GI:85060251; GeneID:3866298; 50S ribosomal protein L22 | -1.21 | 0.981 |
| SG1695; GI:85059673; GeneID:3866278; hypothetical protein | -1.21 | 0.968 |
| SG1570; GI:85059548; GeneID:3866683; ribosomal small subunit pseudouridine synthase | -1.21 | 0.998 |
| GI:125470032; GI:125470032; gp16; hypothetical protein | -1.21 | 0.991 |
| SG0334; GI:85058312; GeneID:3867406; N-acetylmuramoyl-l-alanine amidase II | -1.21 | 0.999 |
| SG1192; GI:85059170; GeneID:3868563; hypothetical protein | -1.21 | 0.956 |
| SG1193; GI:85059171; GeneID:3868564; hypothetical protein | -1.21 | 0.970 |
| SG1937; GI:85059915; GeneID:3867849; phosphatidate cytidylyltransferase | -1.21 | 0.999 |
| SG1839; GI:85059817; GeneID:3868632; hypothetical protein | -1.21 | 0.999 |
| SGtRNA63; GeneID:3867385; tRNA-Gln | -1.21 | 0.991 |
| SG0108; GI:85058086; GeneID:3867981; hypothetical protein | -1.22 | 0.999 |
| SG2252; GI:85060230; GeneID:3866729; 50S ribosomal protein L17 | -1.22 | 0.995 |
| SG0629; GI:85058607; GeneID:3868448; hypothetical protein | -1.22 | 0.999 |
| SG1196; GI:85059174; GeneID:3866835; hypothetical protein | -1.22 | 0.984 |
| SG1187; GI:85059165; GeneID:3868558; hypothetical protein | -1.22 | 0.960 |
| SG1941; GI:85059919; GeneID:3867853; uridylate kinase | -1.22 | 0.991 |
| SG1198; GI:85059176; GeneID:3866837; putative phage lysis protein | -1.23 | 0.987 |
| SG2339; GI:85060317; GeneID:3867167; hypothetical protein | -1.23 | 0.995 |
| SG0581; GI:85058559; GeneID:3867695; putative sigma-54 modulation protein | -1.23 | 0.963 |
| SG1433; GI:85059411; GeneID:3866936; putative ABC transporter ATP-binding component | -1.23 | 0.998 |
| SG2424; GI:85060402; GeneID:3868423; putative transcriptional regulator | -1.23 | 0.998 |
| SGtRNA33; GeneID:3867923; tRNA-Val | -1.23 | 0.994 |
| SG0940; GI:85058918; GeneID:3868427; putative phage DNA injection protein | -1.23 | 0.999 |
| SG0133; GI:85058111; GeneID:3867967; 50S ribosomal subunit protein L7/L12 | -1.23 | 0.988 |
| SG1916; GI:85059894; GeneID:3867649; ABC transporter permease component | -1.23 | 1.000 |
| SG0279; GI:85058257; GeneID:3868586; conserved hypothetical protei | -1.24 | 0.999 |
| GI:125470049; GI:125470049; gp34; hypothetical protein | -1.24 | 0.998 |
| SG2131; GI:85060109; GeneID:3867292; aromatic-amino-acid aminotransferase | -1.24 | 1.000 |
| SG0775; GI:85058753; GeneID:3868042; putative transcriptional regulator | -1.24 | 1.000 |
| SG1043; GI:85059021; GeneID:3866955; hypothetical protein | -1.24 | 1.000 |
| SG1199; GI:85059177; GeneID:3866838; phage lysozyme lysis protein | -1.24 | 0.986 |
| SG1975; GI:85059953; GeneID:3866783; exodeoxyribonuclease V gamma subunit | -1.24 | 0.999 |
| SG1703; GI:85059681; GeneID:3866449; PTS system glucose-specific IIA component Crr | -1.24 | 0.999 |
| SG2394; GI:85060372; GeneID:3868093; dihydroxyacid dehydratase | -1.24 | 0.999 |
| SG1190; GI:85059168; GeneID:3868561; hypothetical protein | -1.25 | 0.981 |
| SG0436; GI:85058414; GeneID:3867411; 3-isopropylmalate dehydrogenase | -1.25 | 0.999 |
| SG0987; GI:85058965; GeneID:3866880; pyruvate formate-lyase 1 activating enzyme | -1.25 | 0.958 |
| SG2391; GI:85060369; GeneID:3868090; ketol-acid reductoisomerase | -1.25 | 0.999 |
| SG2269; GI:85060247; GeneID:3866294; 30S ribosomal protein S17 | -1.25 | 0.992 |
| SG1674; GI:85059652; GeneID:3868538; putative manganese transport protein MntH | -1.25 | 0.999 |
| SG1197; GI:85059175; GeneID:3866836; hypothetical protein | -1.26 | 0.972 |
| SG2179; GI:85060157; GeneID:3867953; hypothetical protein | -1.26 | 0.992 |
| SG2244; GI:85060222; GeneID:3866721; putative DNA topoisomerase | -1.26 | 1.000 |
| SG1057; GI:85059035; GeneID:3866697; fatty acid/phospholipid synthesis protein | -1.26 | 0.999 |
| SGP1_0012; GI:85060423; GeneID:3866551; replication associated protein RepA1 | -1.27 | 0.998 |
| SG1689; GI:85059667; GeneID:3866272; hypothetical protein | -1.27 | 0.988 |
| SG1490; GI:85059468; GeneID:3868177; hypothetical protein | -1.27 | 1.000 |
| SG1681; GI:85059659; GeneID:3866264; phage lysozyme lysis protein | -1.27 | 0.994 |
| SG1117; GI:85059095; GeneID:3867545; ABC transporter ATP-binding component | -1.27 | 1.000 |
| SG1687; GI:85059665; GeneID:3866270; hypothetical protein | -1.27 | 0.997 |
| SG1430; GI:85059408; GeneID:3866933; hypothetical protein | -1.27 | 1.000 |
| SG0880; GI:85058858; GeneID:3868328; cytochrome d ubiquinol oxidase subunit I | -1.28 | 0.999 |
| SG1188; GI:85059166; GeneID:3868559; hypothetical protein | -1.28 | 0.995 |
| SG0937; GI:85058915; GeneID:3868385; hypothetical protein | -1.28 | 1.000 |
| SG1061; GI:85059039; GeneID:3866701; acyl carrier protein | -1.28 | 0.982 |
| SG1685; GI:85059663; GeneID:3866268; hypothetical protein | -1.28 | 0.997 |
| SG0466; GI:85058444; GeneID:3868206; pyruvate dehydrogenase complex repressor | -1.28 | 1.000 |
| SG1178; GI:85059156; GeneID:3868549; hypothetical protein | -1.29 | 0.998 |
| SG2333; GI:85060311; GeneID:3867318; gluconate utilization operon repressor | -1.29 | 1.000 |
| SG2207; GI:85060185; GeneID:3868752; 50S ribosomal protein L33 | -1.29 | 0.995 |
| SG0461; GI:85058439; GeneID:3868201; dephospho-CoA kinase | -1.29 | 1.000 |
| SG1179; GI:85059157; GeneID:3868550; phage replication protein | -1.29 | 0.998 |
| SG1420; GI:85059398; GeneID:3866981; 50S ribosomal protein L35 | -1.29 | 0.999 |
| SG0240; GI:85058218; GeneID:3868292; putative lipid A core surface polymer ligase | -1.30 | 1.000 |
| SG1572; GI:85059550; GeneID:3866685; 50S ribosomal protein L25 | -1.30 | 0.993 |
| SG0388; GI:85058366; GeneID:3867635; intracellular peptidase, PfpI family | -1.30 | 1.000 |
| SGP2_0023; GI:85060489; GeneID:3866601; hypothetical protein | -1.30 | 0.998 |
| SG1263; GI:85059241; GeneID:3867870; zinc ABC transporter ATP-binding protein | -1.30 | 1.000 |
| SG1191; GI:85059169; GeneID:3868562; hypothetical protein | -1.30 | 0.997 |
| SG1421; GI:85059399; GeneID:3866982; 50S ribosomal protein L20 | -1.31 | 0.992 |
| SG0085; GI:85058063; GeneID:3867745; cell division ATP-binding protein | -1.31 | 1.000 |
| SG2007; GI:85059985; GeneID:3868786; Z-ring associated protein ZapA | -1.31 | 0.999 |
| SGP2_0021; GI:85060488; GeneID:3866599; hypothetical protein | -1.31 | 0.999 |
| SGtRNA55; GeneID:3867377; tRNA-Val | -1.32 | 1.000 |
| SG0193; GI:85058171; GeneID:3866632; hypothetical protein | -1.32 | 1.000 |
| SGP1_0048; GI:85060459; GeneID:3866587; hypothetical protein | -1.32 | 1.000 |
| SG2270; GI:85060248; GeneID:3866295; 50S ribosomal protein L29 | -1.32 | 0.999 |
| SG0374; GI:85058352; GeneID:3867486; protein-export protein SecG | -1.32 | 1.000 |
| SG1323; GI:85059301; GeneID:3867518; hypothetical protein | -1.32 | 1.000 |
| SG0657; GI:85058635; GeneID:3868003; geranyltranstransferase | -1.32 | 1.000 |
| SG0003; GI:85057981; GeneID:3867683; DNA metabolism protein RecF | -1.32 | 1.000 |
| SG1194; GI:85059172; GeneID:3866833; hypothetical protein | -1.32 | 0.999 |
| SG0386; GI:85058364; GeneID:3867633; putative acetyltransferase | -1.33 | 1.000 |
| SG0577; GI:85058555; GeneID:3867691; transcriptional regulator, CadC | -1.33 | 1.000 |
| SG1686; GI:85059664; GeneID:3866269; hypothetical protein | -1.34 | 0.999 |
| SG1908; GI:85059886; GeneID:3867641; tRNA/rRNA methyltransferase, TrmH family (SpoU) | -1.36 | 1.000 |
| SG0412; GI:85058390; GeneID:3867581; 30S ribosomal protein S20 | -1.37 | 1.000 |
| SG1112; GI:85059090; GeneID:3868722; putative succinoglycan biosynthesis ketolase | -1.37 | 1.000 |
| SG1320; GI:85059298; GeneID:3867515; cold shock protein | -1.37 | 0.997 |
| SG1100; GI:85059078; GeneID:3868710; cold shock-like protein | -1.37 | 0.992 |
| SG1684; GI:85059662; GeneID:3866267; hypothetical protein | -1.37 | 0.999 |
| SGtRNA15; GeneID:3867017; tRNA-Met | -1.38 | 0.999 |
| SG1195; GI:85059173; GeneID:3866834; hypothetical protein | -1.38 | 0.999 |
| SG2178; GI:85060156; GeneID:3867952; 6-phosphofructokinase | -1.39 | 1.000 |
| SG1025; GI:85059003; GeneID:3867211; putative ribosome modulation factor | -1.39 | 1.000 |
| SG0988; GI:85058966; GeneID:3866881; formate acetyltransferase 1 | -1.40 | 0.999 |
| SG0230; GI:85058208; GeneID:3868062; hypothetical protein | -1.41 | 1.000 |
| SG0380; GI:85058358; GeneID:3867627; 30S ribosomal protein S15 | -1.43 | 1.000 |
| SG1072; GI:85059050; GeneID:3866712; hypothetical protein | -1.45 | 1.000 |
| SG1796; GI:85059774; GeneID:3866799; autonomous glycyl radical cofactor GrcA | -1.46 | 1.000 |
| SG1631; GI:85059609; GeneID:3867154; hypothetical protein | -1.48 | 1.000 |
| SG1774; GI:85059752; GeneID:3866518; hypothetical protein | -1.50 | 1.000 |
| SG0989; GI:85058967; GeneID:3866882; putative formate transport protein | -1.50 | 1.000 |
| SG0145; GI:85058123; GeneID:3868511; major cold shock protein | -1.51 | 1.000 |
| SG0952; GI:85058930; GeneID:3868439; putative lipoprotein | -1.53 | 1.000 |
| SG0602; GI:85058580; GeneID:3866670; putative invasin | -1.59 | 1.000 |
| SG0186; GI:85058164; GeneID:3866625; metal ion ABC transporter permease component | -1.73 | 1.000 |
| SG0185; GI:85058163; GeneID:3866624; metal ion ABC transporter ATP-binding component | -1.77 | 1.000 |
| SG0603; GI:85058581; GeneID:3866671; hypothetical protein | -1.78 | 1.000 |
| SG0187; GI:85058165; GeneID:3866626; metal ion ABC transporter periplasmic component | -1.87 (-2.74) | 1.000 |
| SG0384; GI:85058362; GeneID:3867631; putative collagenase | -1.95 | 1.000 |
| SG1466; GI:85059444; GeneID:3867283; dethiobiotin synthase | -2.73 | 1.000 |

* PPDE - a posteriori probability of differential gene expression
