# Supplementary material for: Quorum Sensing Primes the Oxidative Stress Response in the Insect Endosymbiont, Sodalis glossinidius
Source: PLoS One. 2008 Oct 28;3(10):e3541. doi: 10.1371/journal.pone.0003541 (PMC2568817; doi:10.1371/journal.pone.0003541)
Supplement: Supplementary Information S1 — (0.03 MB DOC) [file pone.0003541.s003.doc]

**Supplementary Information S3. Accession Numbers.**

The GenBank ([http://www.ncbi.nlm.nih.gov/Genbank](http://ukpmc.ac.uk/redirect3.cgi?&&reftype=extlink&artid=504141&iid=13436&jid=195&FROM=Article|Body&TO=External|Link|URI&article-id=504141&journal-id=195&rendering-type=normal&&http://www.ncbi.nlm.nih.gov/Genbank)) accession numbers for the genes and gene products used for the phylogenetic and evolutionary analyses are: *A. tumefaciens* TraI*,* NP_059761; *A. tumefaciens* TraR, NP_059701; *Burkholderia pseudomallei bpmI*, NZ_ABBI01001951; *B. pseudomallei* PmlR, YP_110896; *E. carotovora* *carA*, U17224; *E. carotovora carR,* AF041840; *E. carotovora expI*, AY507108; *E. carotovora* *expR2*, DQ333187; *E. chrysanthemi expI*, EU142019; *E. chrysanthemi expR*, EU142019; *P. aeruginosa* RhlI, NP_252166; *P. aeruginosa rhlR*, L08962; *Pseudomonas chlororaphis phzI*, AY927995; *P. chlororaphis phzR*, AY927995; *Rhizobium leguminosarum* TraI, ZP_02293701; *Sinorhizobium meliloti* TraR, YP_001965652; *S. glossinidius* CarA, YP_454266; *S. glossinidius* SogI, YP_453964; *S. glossinidius* SogR1, YP_453965; *S. glossinidius* SogR2, YP_455420; SOPE accession numbers to be obtained from GenBank; *V. fischeri* LuxI, ZP_02136616; *V. fischeri* LuxR, ZP_02136615; *Vibrio salmonicida luxI*, AF452135; *Yersinia enterocolitica* YenI, YP_001005892; *Y. enterocolitica* YenR, YP_001005891; *Yersinia pestis* YpeI, NP_669050; *Y. pestis* YpeR NP_669049; *Yersinia pseudotuberculosis* YpsI, YP_001400525; *Y. pseudotuberculosis* *ypsR*, AF079973.
